# Real-space spin-orbit interaction from quarter-wave plate nonuniformity

**DOI:** 10.1038/s41467-026-77581-2

**Published:** 2026-09-09

**Authors:** Wenze Lan, Anton Lögl, Peng Fu, Duy Anh Tran, Meryem Benelajla, Clemens Schäfermeier, Khaled Karrai, Bernhard Urbaszek

**Affiliations:** 1 Institute for Condensed Matter Physics, Technische Universität Darmstadt, Darmstadt, Germany; 2 Laboratoire Photonique Numérique et Nanosciences (LP2N), Université de Bordeaux, Institut d’Optique Graduate School, CNRS, Talence, France; 3 eyeo BV, Eindhoven, Netherlands; 4 attocube systems GmbH, Haar, Germany

**Keywords:** Optical physics, Optical physics

## Abstract

Spin–orbit interactions couple light polarization to its transverse structure. Although widely studied at interfaces, in tilted anisotropic plates, and under tight focusing, their role in paraxial confocal systems remains unclear. We show that a commercial quarter-wave plate between crossed polarizers at nominal normal incidence converts the rejected Gaussian field into a Hermite–Gaussian-like two-lobe mode. Its reduced overlap with the collecting fiber mode enhances the polarization extinction ratio by over two orders of magnitude. For an ideal plate at normal incidence, the leading momentum-space contribution vanishes by symmetry. An effective angular model reproduces the symmetry but not the absolute intensity or the beam-size dependence. We trace this discrepancy to first-order real-space nonuniformities of the plate, amplified by the confocal geometry and isolated through beam-size scaling. Cross-polarization confocal detection thus renders weak spatially varying polarization responses observable as transverse-mode transformations.

## Abstract (web summary)

Spin–orbit interactions of light can couple polarization to spatial beam structure. Here, authors show that quarter-wave plate nonuniformity produces a real-space spin–orbit effect that can dominate angular contributions in confocal geometry.

## Introduction

Spin–orbit interactions (SOI) of light describe the coupling between polarization and the spatial or momentum degrees of freedom of an optical field^1,2^. They arise when the polarization-dependent response experienced by a finite beam varies with transverse wave vector or transverse position, thereby coupling polarization-dependent phase or amplitude variations to the transverse field profile. This framework encompasses spatially varying Pancharatnam–Berry phase effects^3–5^, spin-dependent beam shifts^6,7^, spin-to-orbital angular-momentum conversion^8,9^, polarization singularities^10–12^, and optical skyrmions^13,14^. A prominent example is the spin Hall effect of light^15–20^, in which opposite circular-polarization components acquire opposite transverse shifts upon reflection, transmission, or propagation through a polarization-selective element. At planar interfaces, such transverse shifts are closely related to the Imbert–Fedorov effect^21^, while the same wave-vector expansion also describes longitudinal Goos–Hänchen shifts^22^. Both can be obtained by expanding the Fresnel response to first order in transverse wave vector^7,16,23–25^. The corresponding momentum-space Jones-matrix framework has been extended to uniaxial plates^26^, polymer films^27^, linear polarizers^28–30^, and metamaterials^31^. For an ideal laterally uniform birefringent plate at exact normal incidence, however, the first-order transverse-wave-vector terms vanish by symmetry. Experiments designed to observe these weak corrections therefore typically break the symmetry by tilting the optical element and amplify the resulting response through near-orthogonal pre- and post-selection^7,26,30,32–36^.

Such weak spatial signatures become particularly consequential in high-extinction cross-polarization detection. In confocal and resonance-fluorescence measurements, reflection-induced mode reshaping between crossed polarizers can enhance polarization extinction^37^, improve single-photon contrast^38^, and support the laser-rejection strategies used in coherent single-photon sources^39–42^. Wave plates are routinely inserted into these optical paths for polarization control and compensation^40^. Unlike q-plates and metasurfaces, whose spatially varying optic axes are deliberately engineered to convert polarization into spatial structure^43,44^, a commercial quarter-wave plate (QWP) is conventionally represented as a spatially uniform retarder that modifies polarization without reshaping the transverse beam mode. This approximation is generally adequate in conventional intensity measurements, but its validity has not been established in the high-extinction limit, where weak residual fields and small modal changes can dominate the detected signal.

Here we show that this standard idealization fails in high-extinction confocal detection. Inserting a single commercial QWP between crossed polarizers in a minimal confocal geometry, with a well-collimated beam at nominal normal incidence, increases the measured extinction ratio by more than two orders of magnitude. Spatially resolved measurements reveal that the improvement is modal as well as polarimetric: after cancellation of the mean leakage field, the rejected cross-polarized light is reshaped from an attenuated Gaussian into a first-order Hermite–Gaussian-like two-lobe mode carrying approximately 10^−^^7^ of the co-polarized peak intensity. Its orientation follows the QWP fast axis, while its overlap with the collecting single-mode fiber is strongly suppressed. A symmetry-based modal description captures the measured fields, but does not by itself determine their physical origin. For an ideal laterally uniform plate at normal incidence, the leading angular SOI contribution vanishes by symmetry; when effective symmetry-breaking angular terms are introduced, they reproduce the two-lobe symmetry but not the measured intensity scale or beam-size dependence. We therefore expand the response of the physical QWP to first order in real space, retaining weak transverse gradients of its polarization response. The angular and real-space mechanisms generate the same lowest-order modal symmetry but predict opposite beam-size trends, providing a direct experimental means of distinguishing them. The two-lobe mode is observed across commercial QWPs of different materials, while beam-size measurements show an increasing signal inconsistent with the angular mechanism and identify an effective first-order real-space nonuniformity of the QWP as the dominant contribution. A nominally uniform bulk optical element can therefore act as a weak polarization-controlled spatial-mode converter in a configuration where the leading angular SOI terms are symmetry-forbidden, and high-extinction confocal detection provides a sensitive probe of spatially varying polarization responses that remain hidden in conventional integrated measurements.

## Results

### Enhanced polarization extinction ratio

**Fig. 1:**
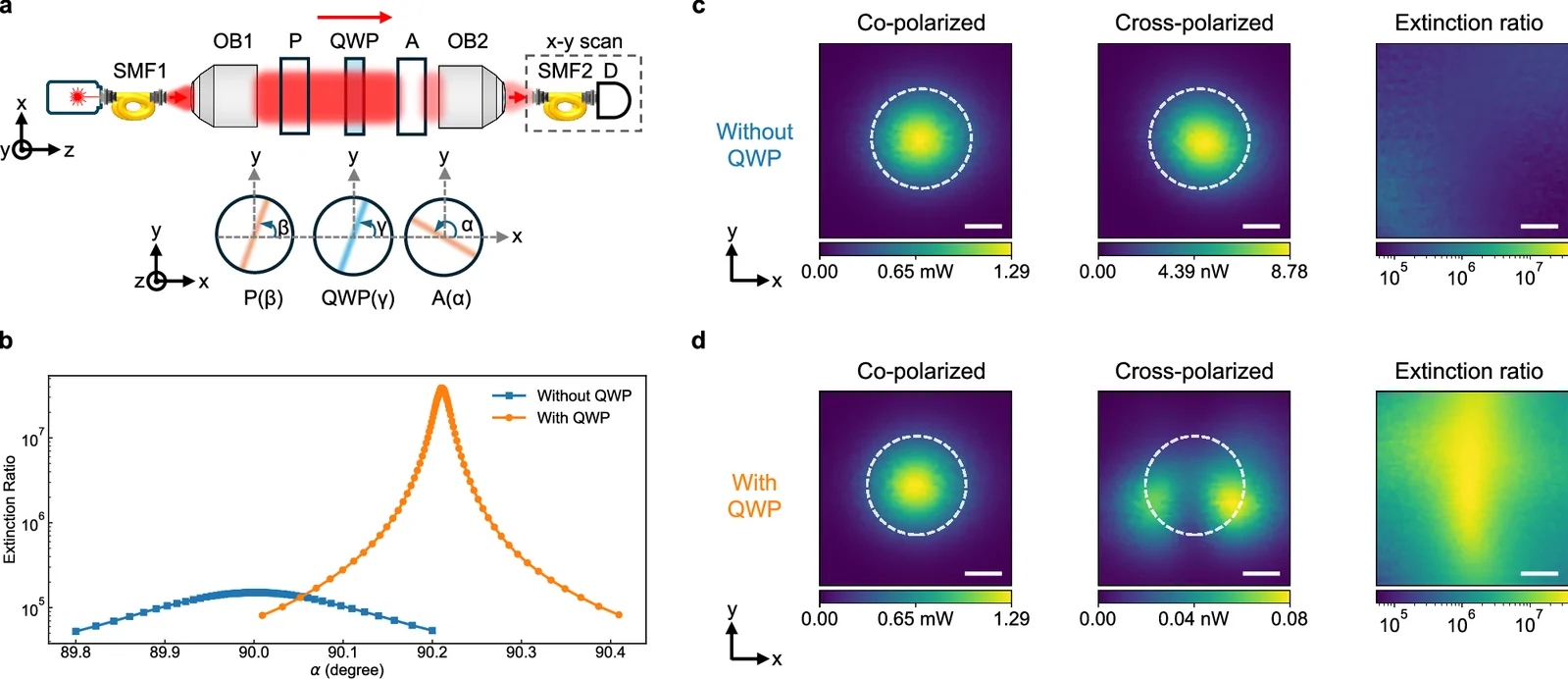
Experimental configuration and polarization-dependent confocal response. **a** Confocal setup. Light from a single-mode fiber (SMF1) is collimated by objective OB1, passes through a polarizer *P* (angle *β*), a quarter-wave plate (QWP, fast-axis angle *γ*) and an analyzer *A* (angle *α*), and is refocused by OB2 into a scanning single-mode fiber (SMF2) and photodiode (*D*). Scanning SMF2 in the focal plane maps the transmitted field. All angles *β*, *γ*, *α* are measured counterclockwise from the laboratory  + *x* axis, viewed against the propagation direction; all elements are at nominal normal incidence. **b** Extinction ratio versus analyzer angle, without (blue) and with (orange) the QWP, starting from *β* = 0°, *γ* = 0°, *α* = 90°. Inserting the QWP raises the optimized extinction ratio from  ~ 10^5^ to  ~ 10^7^ and shifts the extinction maximum by 0.209°. **c**, **d** Co-polarized and cross-polarized confocal maps, and the corresponding extinction-ratio map (co-polarized divided by cross-polarized, pixel by pixel), without (**c**) and with (**d**) the QWP. Without the QWP both channels are Gaussian; with the QWP the cross-polarized field splits into two lobes along *x*, producing an intensity node at the fiber position and raising the local extinction to  ~ 10^7^. The white dashed circle has a diameter equal to the 1/*e*^2^-intensity waist diameter 2*ω*_*f*_ of the unconvolved focal spot at the collecting fiber. Scale bar: 2.5 *μ*m.

We employ a simplified confocal arrangement [Fig. 1a], designed to isolate the essential physics underlying the enhanced laser rejection (see Methods for details). With only the polarizer and analyzer in the beam path, the extinction ratio is limited to  ~ 10^5^ [blue curve, Fig. 1b], set by the intrinsic leakage of the commercial polarizers. Inserting a zero-order quartz QWP between them raises the measured extinction ratio by more than two orders of magnitude, to  ~ 10^7^ [orange curve, Fig. 1b].

We first establish the polarimetric baseline using a plane-wave Jones-matrix description. For the polarizer–analyzer pair, it relates the residual polarizer leakage *b* to a small analyzer offset from the crossed-polarization condition (Suppl. Note 1). Experimentally, the optimal analyzer angle shifts by 0.209° when the QWP is inserted; interpreting this shift as an effective leakage angle gives $b=| \cos (9{0}^{\circ }+0.20{9}^{\circ })| \approx 3.6\times 1{0}^{-3}$, consistent with reported values for high-extinction commercial polarizers^37,38^. The corresponding without-QWP extinction, 1/*b*^2^ ≈ 0.8 × 10^5^, agrees with the measured value in Fig. 1b. The  ~ 10^7^ value is specific to this implementation rather than a limit of the mechanism: in the exact plane-wave limit, the polarizer–QWP–analyzer geometry admits complete cancellation of the mean leakage (Suppl. Note 1), so the achievable extinction is limited in practice by the plate retardance and by the finite angular optimization; a plane-wave Jones-matrix search over representative angular windows and step sizes yields optimized values in the 10^7^–10^8^ range (Suppl. Fig. 1). Consistently, a polymer zero-order QWP reaches an extinction ratio beyond 10^9^ under the same confocal detection scheme; that value is limited by the overall experimental readout noise floor and therefore represents a lower bound (Suppl. Fig. 5a).

The QWP therefore supplies the additional degree of freedom required to cancel the mean, spatially uniform leakage field, which a polarizer–analyzer pair alone cannot achieve (Suppl. Note 1). A half-wave-plate control supports this assignment: for a zero-order HWP no combination of polarizer and analyzer angles cancels the leakage, and the measured extinction remains limited to  ~ 10^6^ with a Gaussian cross-polarized mode (Suppl. Fig. 2). The finite-beam question is then the spatial structure of the residual field after mean-leakage cancellation. Bliokh et al. showed that a tilted birefringent plate can redistribute the cross-polarized field into a two-lobe, Hermite–Gaussian (HG)-like distribution with a central dark point^26^. In our experiment, the rejected field at the extinction optimum is likewise not a uniformly attenuated Gaussian but a first-order transverse mode whose overlap with the collecting single-mode fiber is strongly suppressed. The enhanced laser rejection is thus a two-step mechanism—QWP-enabled polarimetric cancellation of the mean leakage, followed by confocal mode filtering of the reshaped residual—and we now examine the modal transformation directly.

### Modal transformation of a transmitted polarized Gaussian beam

We map the detected intensity by scanning the collecting single-mode fiber (SMF) in the focal plane of the objective. The spatial filtering is set by the Gaussian mode of this SMF, so the detected signal is the overlap between the focal-plane field and the fiber mode. Without the QWP, the co- and cross-polarized confocal maps in Fig. 1c are both HG_00_-like Gaussian modes and the extinction-ratio map reaches only  ~ 10^5^, consistent with the polarizer leakage. Inserting the QWP leaves the co-polarized map Gaussian [Fig. 1d] but reshapes the cross-polarized field into a two-lobe pattern with a minimum at the nominal fiber position. This modal redistribution suppresses the overlap with the collecting fiber and raises the detected extinction to  ~ 10^7^. Direct free-space camera imaging confirms that the two-lobe field exists before the focusing objective and is therefore not an artifact of the scanning-fiber detection (Suppl. Fig. 4).

**Fig. 2:**
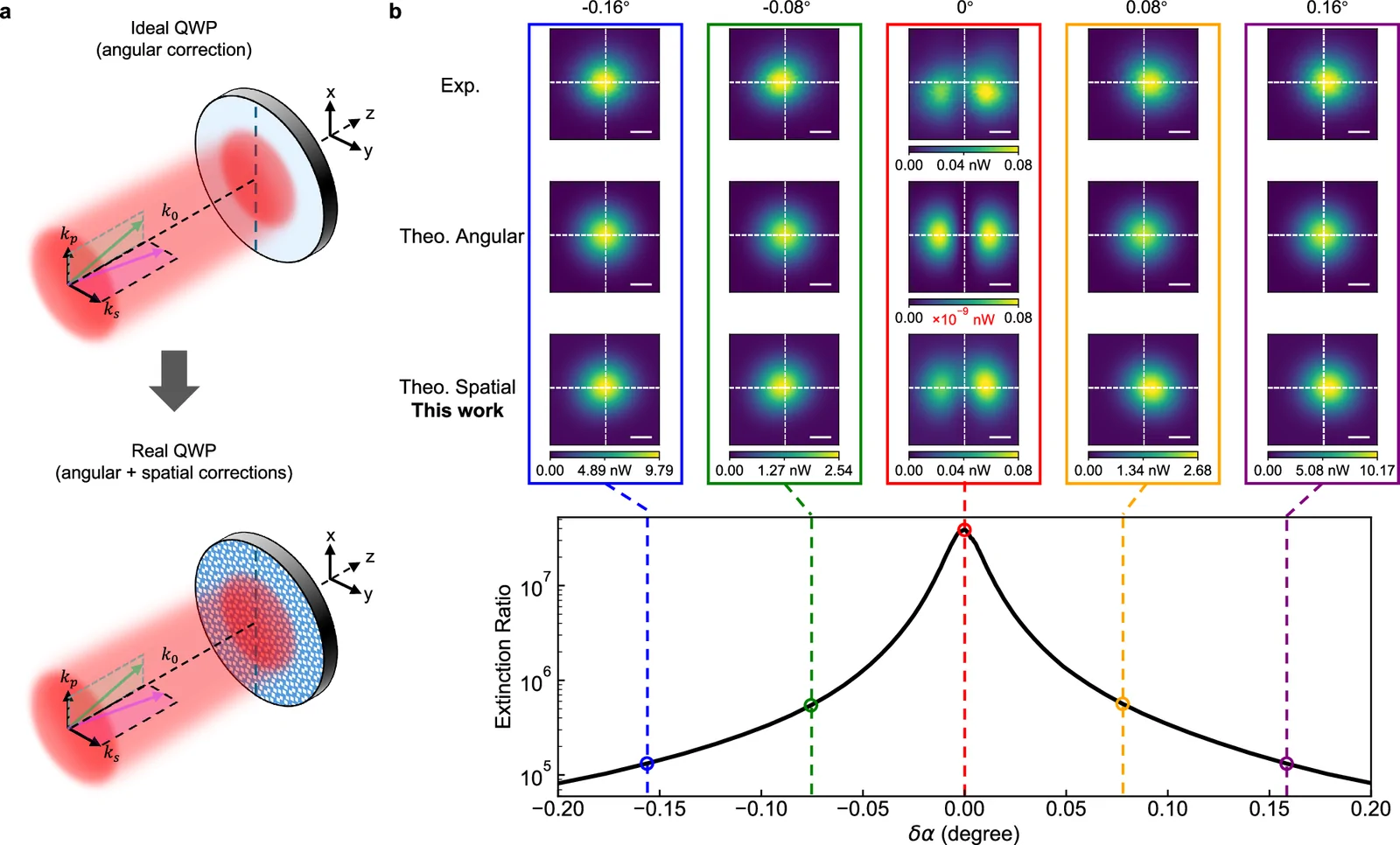
Physical model and analyzer-dependent modal evolution. **a** An ideal laterally uniform quarter-wave plate (QWP) responds only to the transverse momentum components (axes *k*_*x*_, *k*_*y*_ in the figure; the equivalent fast/slow-axis components *k*_*p*_, *k*_*s*_ appear in Eqs. (1) and (5)), whereas a real QWP additionally carries first-order real-space gradients of its local Jones response. **b** Cross-polarized confocal maps for analyzer detunings $\delta \alpha \equiv \alpha -{\alpha }_{\max }$ across the extinction maximum (lower panel; ${\alpha }_{\max }$ is the analyzer angle of maximum extinction), comparing experiment (Exp.), the angular-only theory (Theo. angular) and the spatial theory (Theo. spatial). Away from the extinction point, residual leakage dominates and the angular-only model follows the data. At exact extinction (*δα* = 0) the ideal normal-incidence angular contribution vanishes by symmetry, so the angular-only calculation returns a numerical zero ( < 10^−20^ W, the numerical noise floor of the calculation), whereas the experiment retains a two-lobe mode at the nanowatt scale. Including first-order real-space corrections reproduces both the two-lobe structure and the observed intensity scale. Note the individual intensity scale of each panel. Scale bar: 2.5 *μ*m.

We first compare this observation with the established momentum-domain Jones-matrix framework for anisotropic optical elements^16,26,29,30^. For a laterally uniform QWP (upper panel of Fig. 2a), the Jones matrix can be expanded to first order in the transverse wave-vector components *k*_*p*_ and *k*_*s*_ along the fast and slow axes, respectively. Writing *k*_0_ = 2*π*/*λ*, this expansion takes the form

1 $$\bar{Q}({k}_{p},{k}_{s})\simeq {\bar{Q}}_{0}+\frac{{k}_{p}}{{k}_{0}}{\bar{Q}}_{1}+\frac{{k}_{s}}{{k}_{0}}{\bar{Q}}_{2},$$

where the first-order matrices are

2 $${\bar{Q}}_{1}=\left(\begin{array}{rc}{e}^{-i{{\Phi }}_{0}/2}{{{{\mathcal{X}}}}}_{e}&0\\ 0&{e}^{+i{{{\Phi }}}_{0}/2}{{{{\mathcal{X}}}}}_{o}\end{array}\right),$$

3 $${\bar{Q}}_{2}=\left(\begin{array}{rc}0&{e}^{-i{{{\Phi }}}_{0}/2}{{{{\mathcal{Y}}}}}_{e}\\ -{e}^{+i{{{\Phi }}}_{0}/2}{{{{\mathcal{Y}}}}}_{o}&0\end{array}\right),$$

with

4 $${{{{\mathcal{X}}}}}_{e,o}=\mp \frac{i}{2}\frac{d{{\Phi }}}{d\theta },\qquad {{{{\mathcal{Y}}}}}_{e,o}=\left[1-\exp (\pm i{{{\Phi }}}_{0})\right]\cot \theta .$$

Together, ${\bar{Q}}_{1}$ and ${\bar{Q}}_{2}$ describe the angular spin–orbit response derived in Suppl. Note 2. This is the same class of expansion used for spin-Hall beam shifts at interfaces^7,16,23,24^, tilted plates^26^ and polarization-selective elements^28–30^. Its defining property here is symmetry: for an ideal QWP at exact normal incidence, ${\bar{Q}}_{1}$ and ${\bar{Q}}_{2}$ vanish. This follows because *d*Φ/*dθ* → 0 at normal incidence (*θ* = 90°), so ${{{{\mathcal{X}}}}}_{e,o}\to 0$, and because the $\cot \theta$ factor in ${{{{\mathcal{Y}}}}}_{e,o}$ vanishes at the same point. As a result, the angular mechanism predicts no first-order two-lobe mode. A finite angular response requires symmetry breaking, such as a deliberate tilt, as exploited in weak-measurement experiments^7,26,30,33^. In our nominal-normal-incidence geometry, the residual angular contribution can be estimated: taking the vendor-specified angle-of-incidence retardance curve and an illustrative residual mount tilt of  ~ 1 mrad as the symmetry-breaking scale yields a normalized lobe intensity of  ~ 4 × 10^−^^11^, more than three orders of magnitude below the measured 10^−7^ level (Suppl. Note 3), consistent with the numerically vanishing angular-only prediction of Fig. 2b. The angular response therefore accounts for the modal symmetry but not for the observed intensity scale.

We therefore include the lowest-order real-space response of a physical QWP (lower panel of Fig. 2a). A fabricated wave plate can carry weak transverse variations of retardance, fast-axis orientation, thickness, coating response or stress-induced birefringence across the illuminated aperture. The combined first-order response is expressed as an operator. Expanding about the beam center and the central wave vector yields

5 $$\hat{{{{\mathcal{Q}}}}}\simeq {\bar{Q}}_{0}+\frac{\hat{{k}_{p}}}{{k}_{0}}{\bar{Q}}_{1}+\frac{\hat{{k}_{s}}}{{k}_{0}}{\bar{Q}}_{2}+\hat{p}\,{\bar{Q}}_{3}+\hat{s}\,{\bar{Q}}_{4}.$$

Here, $\hat{p}$ and $\hat{s}$ denote the transverse-coordinate operators in the QWP plane along the fast and slow axes, respectively, while ${\hat{k}}_{p}$ and ${\hat{k}}_{s}$ represent the corresponding transverse-wave-vector operators. The matrices ${\bar{Q}}_{3}={\left.\partial \bar{Q}/\partial p\right\vert }_{0}$ and ${\bar{Q}}_{4}={\left.\partial \bar{Q}/\partial s\right\vert }_{0}$ are the local real-space Jones gradients, with units of inverse length (Suppl. Note 3). Unlike the angular terms, these real-space terms are allowed at normal incidence. After propagation through the confocal system, they enter the first-order modal coefficient with a weight proportional to the Rayleigh length $\ell={k}_{0}{\omega }_{0}^{2}/2$ of the collimated beam, where *ω*_0_ is the collimated beam radius at the plate. Considering propagation through the collecting objective, projection by the analyzer, and overlap with the fiber mode, the collected cross-polarized amplitude takes the compact scalar form

6 $${E}_{\perp }({x}_{0},{y}_{0})\propto \exp \left[-\frac{{x}_{0}^{2}+{y}_{0}^{2}}{{\omega }_{f}^{2}+{\omega }_{m}^{2}}\right]\left[{\eta }_{0}+\Lambda \left({G}_{x}{x}_{0}+{G}_{y}{y}_{0}\right)\right],$$

where (*x*_0_, *y*_0_) is the position of the collecting fiber in the focal plane, *η*_0_ is the residual Gaussian leakage normalized to the co-polarized amplitude, *ω*_*f*_ is the focused field radius, *ω*_*m*_ is the fiber mode-field radius, and $\Lambda={\omega }_{f}^{2}/({\omega }_{f}^{2}+{\omega }_{m}^{2})$ is the Gaussian-overlap centroid factor. The complex coefficients *G*_*x*_, *G*_*y*_ are defined in the laboratory frame before this final overlap factor, so that *ΛG*_*x*_ and *ΛG*_*y*_ are the fitted collected coefficients; they are related to the fast/slow-axis coefficients (*G*_*p*_, *G*_*s*_) of Suppl. Note 3 by a rotation through the QWP fast-axis angle. Each coefficient combines an angular and a real-space contribution. In the fast/slow-axis frame,

7 $${G}_{p}=\frac{1}{f}\,{{{{\bf{a}}}}}^{{{\dagger}} }{\bar{Q}}_{1}{{{{\bf{e}}}}}_{{{{\rm{P}}}}}-i\frac{{k}_{0}{\omega }_{0}^{2}}{2f}\,{{{{\bf{a}}}}}^{{{\dagger}} }{\bar{Q}}_{3}{{{{\bf{e}}}}}_{{{{\rm{P}}}}},$$

and similarly for *G*_*s*_ with ${\bar{Q}}_{2},{\bar{Q}}_{4}$, where **a** is the analyzer transmission vector and ${{{{\bf{e}}}}}_{{{{\rm{P}}}}}=\bar{P}(\beta ){{{{\bf{E}}}}}_{0}$ the field after the polarizer. For our parameters (*λ* = 850 nm, *ω*_0_ ≈ 2.8 mm, *f* = 26 mm), the factor ${k}_{0}{\omega }_{0}^{2}/2f=\ell /f\approx 1.1\times 1{0}^{3}$ is the geometric weighting of the real-space gradient term after propagation through the confocal system. Therefore, weak spatial variations of a physical QWP can generate a first-order modal response exceeding the angular one. Importantly, this is not an amplification of the angular SOI itself: the angular and real-space terms are distinct channels that couple to the same first-order odd transverse mode, while the confocal propagation factor enhances the contribution of the real-space gradient. The phase factor ( − *i*) reflects the Fourier relation between a transverse coordinate perturbation at the QWP plane and the derivative mode detected in the focal plane. The terms proportional to *x*_0_ and *y*_0_ are first derivatives of the Gaussian envelope. They change sign across the beam center, producing an HG-like two-lobe distribution with a node near the optical axis. Their orientation is determined by the relative magnitudes and phase of *G*_*x*_ and *G*_*y*_, while the lobe asymmetry arises from interference with the residual Gaussian term *η*_0_. For the first-order component, the lobe maxima lie on a circle of radius $\sqrt{({\omega }_{f}^{2}+{\omega }_{m}^{2})/2}$ (Suppl. Note 3), which fixes the mode geometry without additional free parameters.

Figure 2b presents the confocal maps as a function of analyzer detuning $\delta \alpha \equiv \alpha -{\alpha }_{\max }$, where ${\alpha }_{\max }$ denotes the analyzer angle corresponding to maximum extinction. At large detuning from maximum extinction, the residual Gaussian term dominates, resulting in a single spot in the cross-polarized map. As *δα* approaches zero, Gaussian leakage is suppressed while the derivative terms persist, causing the field to evolve into an HG_10_-like two-lobe pattern. Adjusting the analyzer at the extinction point alters the relative amplitudes and phases of the residual Gaussian and derivative components, thereby switching the dominant lobe. The complete first-order model described by Eq. (6) accurately reproduces this analyzer-dependent modal evolution and its intensity scale. In contrast, the angular-only calculation aligns with the data only away from extinction and predicts a numerically vanishing field at the extinction point. Suppl. Note 7 summarizes the extracted parameters and residuals. The lobe peak positions exhibit an avoided-crossing-like behavior, consistent with observations for mirror-based confocal extinction (see Supp. Fig. 11 and ref. ^37^).

Since every first-order correction, whether angular or real-space, collapses onto the same residual Gaussian and its two transverse derivatives, Eq. (6) serves as a first-order modal parametrization of the collected field rather than a microscopic model of the plate. Fitting to the measured maps determines the effective coefficients *G*_*x*_ and *G*_*y*_, but does not independently resolve the angular contributions of ${\bar{Q}}_{1}$ and ${\bar{Q}}_{2}$, which are symmetry-forbidden at exact normal incidence, from the real-space contributions of ${\bar{Q}}_{3}$ and ${\bar{Q}}_{4}$, which are permitted at normal incidence; both contribute to the same odd mode. The physical distinction between these two channels is demonstrated experimentally by their opposite beam-size scaling, as discussed in the coming section.

### Beam-size scaling and control experiments

The angular and real-space mechanisms both generate a first-order two-lobe field, but they make opposite predictions for its dependence on the collimated beam size. A larger collimated beam has a narrower angular spectrum, so the collected angular-channel lobe signal decreases after focusing and fiber convolution. A real-space plate gradient, by contrast, enters the first-order coefficient with the Rayleigh-length weight $\ell={k}_{0}{\omega }_{0}^{2}/2$ (Suppl. Note 3), so for a fixed local gradient the collected contribution increases with *ω*_0_ over the measured range.

**Fig. 3:**
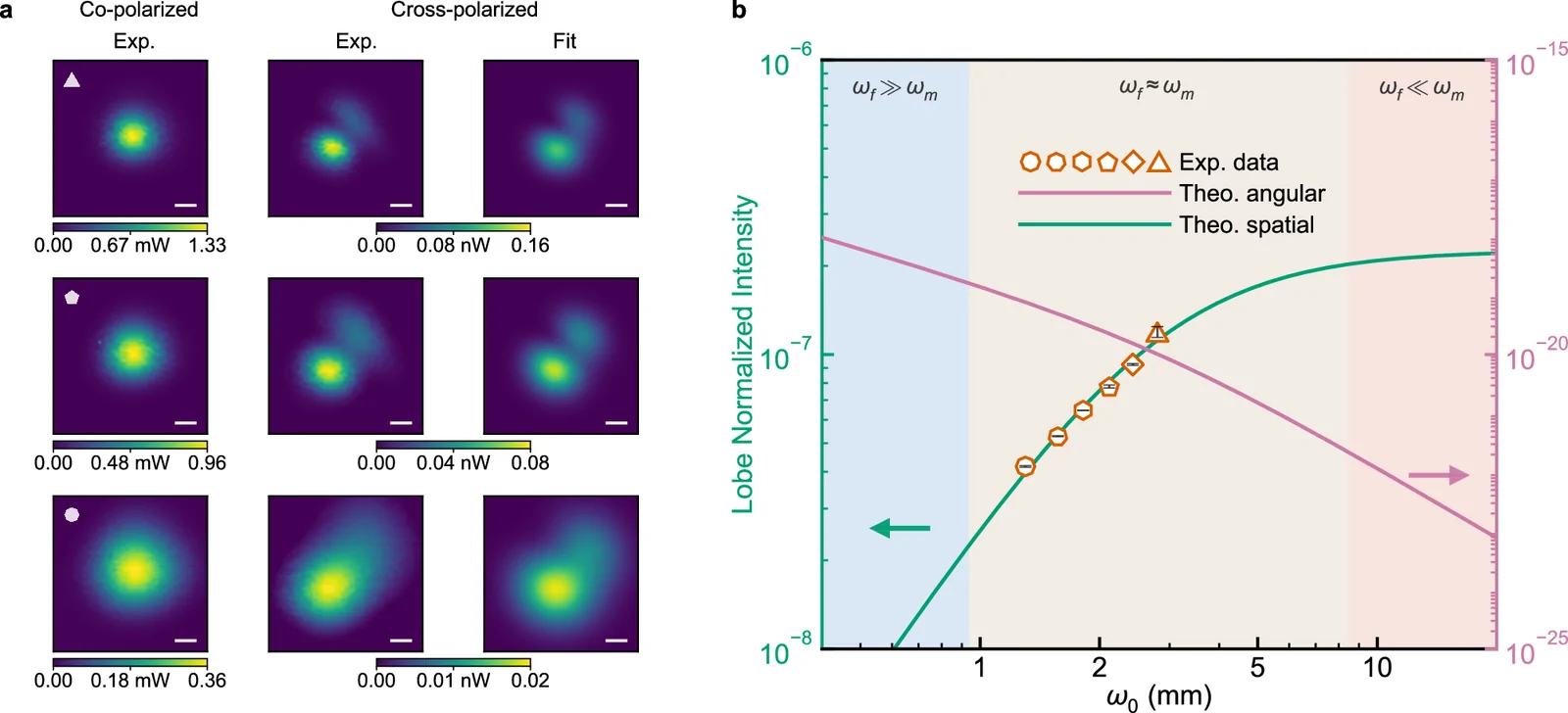
Beam-size scaling discriminates the spatial from the angular mechanism. **a** Confocal maps for three collimated beam radii *ω*_0_ (increasing bottom to top): co-polarized experiment (Exp., left, mW), cross-polarized experiment (Exp., middle, nW) and cross-polarized fit (Fit, right, nW). Each cross-polarized map and its fit share a color scale. The grey symbol in each co-polarized map marks the corresponding point in (**b**). Scale bar: 2.5 *μ*m. **b** Normalized lobe intensity (peak cross-polarized lobe intensity over peak co-polarized intensity) versus *ω*_0_ (log-log); symbols are measured data (Exp. data, shape encodes *ω*_0_, triangle = largest). Error bars: spread between values extracted from forward (trace) and backward (retrace) fiber scans (Methods). Solid curves are the two first-order models, plotted on separate axes because their absolute scales differ by several orders of magnitude: the real-space prediction (Theo. spatial, green, left axis) and the angular-spectrum prediction (Theo. angular, pink, right axis), with arrows indicating each axis. Shaded bands mark the *ω*_*f*_ ≫ *ω*_*m*_, *ω*_*f*_ ≈ *ω*_*m*_ and *ω*_*f*_ ≪ *ω*_*m*_ regimes (*ω*_*f*_: focal-spot radius; *ω*_*m*_: fiber mode-field radius). The measured positive trend follows the spatial curve, identifying real-space QWP nonuniformity as the dominant first-order contribution.

We test this prediction by varying *ω*_0_ and recording the normalized dark-field lobe intensity, defined as the peak cross-polarized lobe intensity divided by the peak co-polarized lobe intensity, measured in the same configuration (see Fig. 3a and Suppl. Fig. 6). The fiber mode radius *ω*_*m*_ is held fixed while the focal-plane radius *ω*_*f*_ = *C*/*ω*_0_ (with *C* = *λf*/*π*) is scanned through *ω*_0_. Inserting the opposite channel scalings into the mode-overlap collection yields the explicit dependence on the scanned variable (Suppl. Note 3),

8 $${R}_{\Theta }^{2}\ \propto \ \frac{1}{{\omega }_{0}^{2}\left({C}^{2}+{\omega }_{m}^{2}{\omega }_{0}^{2}\right)}\quad \,{{\rm{(angular)}}}\,,$$

9 $${R}_{r}^{2}\ \propto \ \frac{{\omega }_{0}^{2}}{{C}^{2}+{\omega }_{m}^{2}{\omega }_{0}^{2}}\quad \,{{\rm{(spatial)}}}\,.$$

Both expressions share the same monotonic overlap denominator, so their opposite behavior in *ω*_0_ is set by the first-order coefficient: ${R}_{\Theta }^{2}$ decreases monotonically while ${R}_{r}^{2}$ increases monotonically. The measured magnitude, of order 10^−7^, corresponds through this scaling to a dimensionless variation of the analyzer-projected Jones response of  ~ 7 × 10^−4^ across one beam radius (Suppl. Note 3). As shown in Fig. 3b, the measured signal increases with *ω*_0_ and follows the real-space prediction of Eq. (9) (green), opposite to the angular prediction of Eq. (8) (pink).

The fitted modal coefficients provide an independent, shape-resolved check. The residual on-axis leakage ∣*η*_0_∣ remains nearly constant over the measured range, whereas the combined fitted magnitude $| {{{{\boldsymbol{G}}}}}^{{{{\rm{fit}}}}}|={(| {G}_{x}^{{{{\rm{fit}}}}}{| }^{2}+| {G}_{y}^{{{{\rm{fit}}}}}{| }^{2})}^{1/2}$, with ${G}_{x,y}^{{{{\rm{fit}}}}}=\Lambda {G}_{x,y}$, grows by a factor  ≈ 2.9. This matches the collected spatial-channel scaling $\Lambda ({\omega }_{0})\,{\omega }_{0}^{2}$ predicted for a fixed plate gradient [factor 2.8; point-by-point agreement within  ≈ 12%], whereas the collected angular channel, which scales as *Λ* alone, would decrease by a factor  ≈ 1.6 over the same range (Suppl. Note 7 and Supplementary Table 4). The positive scaling is reproduced for QWPs of different materials (Suppl. Fig. 7) and is independent of how the beam size is varied: a telescope-based method reproduces the iris result for the same plates (Suppl. Note 4). This plate-dependent positive scaling is the principal quantitative evidence that the observed mode is dominated by a real-space first-order channel absent from the laterally uniform momentum-domain description.

We further perform control measurements to constrain possible alternative origins. The two-lobe mode is observed for quartz, polymer, and MgF_2_ QWPs, for different retardance orders, and at both 850 and 633 nm (Suppl. Fig. 5), excluding a single defective component, a specific material platform, or a laser-source artifact. The observation with MgF_2_ further excludes quartz optical activity as the dominant origin. Deliberately tilting the QWP modifies the lobe orientation and intensity, as expected when angular spin–orbit terms are activated (Suppl. Fig. 10), but the two-lobe mode is already present at nominal normal incidence. An independent Mach–Zehnder measurement (Suppl. Note 6, Suppl. Figs. 15 and 16) quantifies the spatially varying wavefront perturbations that real QWPs imprint on the transmitted beam: after removal of the linear ramp, residual nonuniformities remain at the *λ*/85–*λ*/45 level, numerically below the transmitted-wavefront-error limits quoted by the vendors (Suppl. Notes 6 and 8, Suppl. Tables 1 and 6). This scalar wavefront probe complements the confocal measurement, which is sensitive to the full vectorial response. Finally, a full vectorial Richards–Wolf calculation for an ideal spatially uniform QWP yields symmetry-locked non-paraxial components but no transverse first-order mode after the polarization chain, and therefore cannot reproduce the analyzer- and QWP-dependent two-lobe mode observed experimentally (Suppl. Note 5). Together, these controls identify first-order real-space QWP nonuniformity, amplified by the confocal geometry, as the dominant mechanism behind the observed high-extinction modal conversion.

### Orientation control and the intensity of the rejected mode

**Fig. 4:**
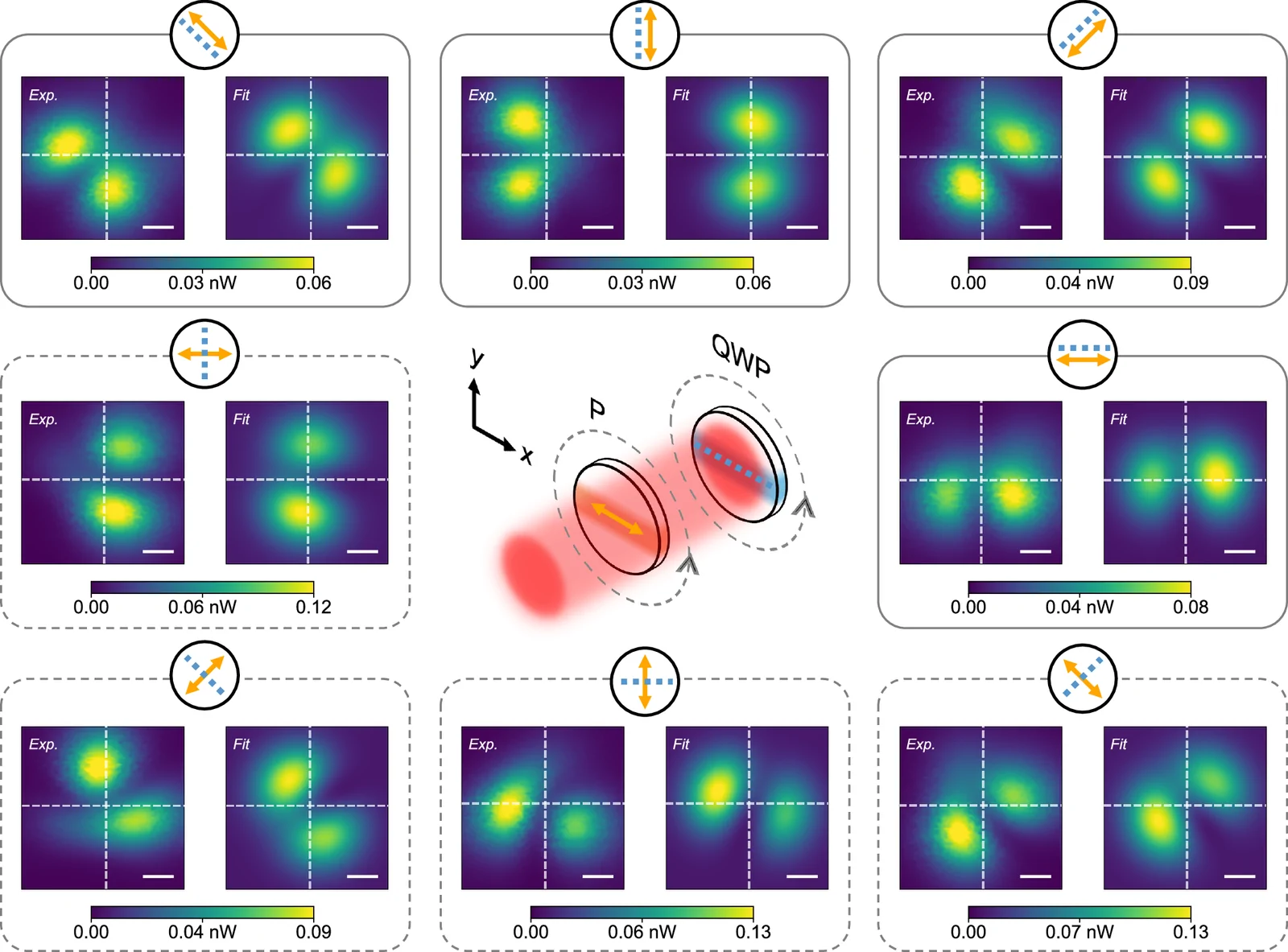
Polarization control of the two-lobe mode orientation. Measured (Exp.) and fitted (Fit) cross-polarized confocal maps for different combinations of incident polarization and quarter-wave plate (QWP) fast-axis orientation; the central schematic defines the polarizer *P* and the QWP in the *x*-*y* frame. In each circular icon the orange arrow is the incident linear polarization and the blue dotted line is the QWP fast axis. Solid frames denote polarization parallel to the fast axis, dashed frames perpendicular to it. Rotating the fast axis rotates the two-lobe pattern continuously, including to  ± 45°, so the orientation is not pinned to the coordinate axes. Dashed white lines mark the co-polarized reference center. Scale bar: 2.5 *μ*m.

The two-lobe mode can be continuously reoriented by rotating the QWP fast axis in the *x*–*y* plane through the angle *γ*, with the input linear polarization parallel (solid frames) or perpendicular (dashed frames) to that axis. As shown in Fig. 4, the lobe orientation follows the fast axis continuously and takes arbitrary orientations in the transverse plane.

This orientation behavior has a simple physical interpretation. Near maximum extinction, the rejected field is dominated by the first transverse derivative of the incident Gaussian taken along a direction fixed by the QWP—a directional derivative ${\partial }_{\hat{{{{\bf{u}}}}}}$ whose axis $\hat{{{{\bf{u}}}}}$ is set by *γ*. The intensity node lies perpendicular to $\hat{{{{\bf{u}}}}}$, so rotating the QWP rotates $\hat{{{{\bf{u}}}}}$ and carries the two-lobe pattern with it. In the scalar model, rotating the QWP redistributes the response between the *x*- and *y*-derivative channels (*G*_*x*_, *G*_*y*_) of Eq. (6), rotating the nodal line accordingly. The orientation control demonstrated here is therefore consistent with the projection rule for near-orthogonal post-selection established in weak-measurement studies of anisotropic elements^30^. The extracted lobe axes and fitted orientation trends are summarized in Supplementary Fig. 12, and a similar reorientation under a deliberate angle of incidence is shown in Supplementary Fig. 10. Both exclude a fixed astigmatism of the setup as the origin of the mode.

What distinguishes the present measurement is the magnitude of the rejected mode. Whereas previous weak-measurement studies characterize this regime primarily through transverse beam shifts^30^, our confocal measurement quantifies the first-order modal-conversion strength through the fitted coefficients (*G*_*x*_, *G*_*y*_), whose magnitude follows the beam-size scaling of the real-space channel identified above.

## Discussion

Our results identify a modal-overlap mechanism for polarization-extinction enhancement in a crossed-polarization confocal geometry. Under high-extinction cross-polarization, a QWP compensates the average polarization leakage and leaves a weak rejected field dominated by a first-order spatial mode. Because this odd mode has small overlap with the Gaussian collecting-fiber mode, the residual leakage is further suppressed by confocal mode filtering. A component usually treated as a spatially uniform retarder can therefore reshape the transverse structure of the rejected light once detection operates close to extinction.

The established momentum-domain SOI formalism accounts for the symmetry of the effect: anisotropic plates generate first-order transverse corrections, and near-orthogonal projection sets the orientation of the two-lobe mode. At nominal normal incidence, however, it does not account for the magnitude, since the ideal angular terms vanish by symmetry and effective angular terms fall short of the measured lobe intensity and beam-size trend. Both are supplied instead by the first-order real-space nonuniformity of the physical QWP, which confocal mode projection amplifies into the dominant contribution to the rejected field. The confocal geometry is not simply a new setting for a known beam shift: it changes the observable from a centroid displacement to a mode-overlap problem. This comparison also suggests why the relative weight of the two channels can differ strongly across precision beam-shift experiments. When a laterally nonuniform polarization element is sampled near a focused waist, the smaller illuminated area suppresses the first-order real-space-gradient contribution according to the beam-size scaling above, whereas the broader angular spectrum and subsequent propagation can enhance the manifestation of angular beam shifts^24^. Near-orthogonal post-selection can further amplify such weak angular signatures^7^. Thus, real- and momentum-space variations should in general be considered together, with their relative importance set by the illumination and detection geometry. In cross-polarized confocal detection, the analyzer-projected real-space gradient appears as a first-order modal amplitude weighted by *ℓ*/*f*. Where confocal mode filtering was previously established for reflection off a mirror^37^, it here exposes the real-space channel of a transmissive element and quantifies it through the intensity of the rejected field.

The fitted coefficients *G*_*x*_ and *G*_*y*_ are effective parameters. They combine retardance and fast-axis gradients, thickness variations, coating response, stress-induced birefringence, and polarization-projection effects accumulated through the confocal system, and they are not a unique microscopic reconstruction of the plate. Separating these contributions calls for a spatially resolved vectorial Jones-matrix measurement, for example, by vectorial ptychography^45^, which the present scheme motivates. The mechanism assignment does not rest on such a decomposition: the angular and real-space channels feed the same odd mode with opposite beam-size trends, and the measured trend is reproduced across plates, materials and wavelengths, consistent with direct imaging, interferometry and vectorial Richards–Wolf simulations. The real-space channel thus complements the momentum-domain spin–orbit framework: corrections normally negligible in polarization optics become observable once a confocal system operates at extinction ratios of 10^7^–10^9^.

In practice, the mechanism offers a simple route to improved polarization rejection in resonance fluorescence and dark-field confocal measurements using standard transmissive optics, without a reflecting surface^37^ or a custom element. High-extinction confocal detection is thereby a sensitive probe of weak spatial-gradient and spin–orbit effects that remain hidden in conventional intensity measurements, and could be developed into a characterization tool for the residual nonuniformity of nominally ideal optical components.

## Methods

### Experimental setup

The compact confocal configuration of Fig. 1a isolates the mechanism responsible for the enhanced laser rejection. A single-mode-fiber-coupled diode laser at *λ* = 850 nm (LDT-850-10G, TOPAG Lasertechnik; angled 4° flat-polished output; mode-field radius *ω*_*m*_ = 2.5 *μ*m at the 1/*e*^2^ intensity level) is collimated by a diffraction-limited microscope objective (NA = 0.25, *f* = 26 mm) into a Gaussian beam of radius *ω*_0_ ≈ 2.8 mm at the QWP (consistent with the fiber-limited value *λf*/(*πω*_*m*_) = 2.81 mm). The collimated beam passes through a high-extinction nanoparticle thin-film linear polarizer (LPVIS050, Thorlabs), a zero-order quartz QWP (WPQ05M-850, Thorlabs) and a second, identical polarizer serving as analyzer; the QWP can be removed for reference measurements. The transmitted light is refocused by an identical objective into a second single-mode fiber, providing confocal spatial-mode filtering, and detected by a photodiode (OE-200-SI-FC, FEMTO) read out on a multimeter (Keysight 34465A). The polarizer and analyzer are mounted on piezoelectric rotation stages (ECR4040/Al/NUM/RT, attocube; angular resolution 0.001°); the QWP is in a precision rotation mount (CRM1PT/M, Thorlabs; Vernier resolution  ≈ 0.083°); and the collecting fiber is on a three-axis piezo/manual translation stage (MDE330TH with PE4, Thorlabs). Extinction optimization is automated in Python by iteratively adjusting the polarizer and analyzer angles with progressively refined step sizes.

### Angle conventions

Unless stated otherwise the QWP is at nominal normal incidence. The polarizer, QWP fast-axis and analyzer angles are denoted *β*, *γ* and *α*, all measured counterclockwise from the laboratory  + *x* axis viewed against the propagation direction. The analyzer detuning is $\delta \alpha \equiv \alpha -{\alpha }_{\max }$, where ${\alpha }_{\max }$ maximizes the extinction ratio for a given polarizer–QWP setting.

### Control measurements

Material, retardance-order and wavelength controls used 850 nm quartz zero-order (WPQ05M-850) and polymer zero-order (WPQ10E-850-SP) QWPs, and 633 nm quartz zero-order (WPQ05M-633) and quartz multi-order (WPMQ05M-633) QWPs from Thorlabs together with an MgF_2_ zero-order QWP (RZM 4.10.0633 RT1.5, Bernhard Halle Nachfl.). The 633 nm measurements used a He–Ne laser (HNL100LB, Thorlabs) and a single-mode fiber (P3-S630-FC, Thorlabs; *ω*_*m*_ ≈ 2.1 *μ*m). For beam-size-dependent measurements the beam radius *ω*_0_ at the QWP was varied with an iris diaphragm placed between the polarizer and the QWP, leaving the optical path, alignment and focusing geometry unchanged across all beam sizes; an independent telescope-based variation of *ω*_0_ reproduces the same scaling behavior (Suppl. Note 4). For each aperture setting the effective Gaussian radius was inferred from the co-polarized confocal data: the fitted envelope radius ${({\omega }_{f}^{2}+{\omega }_{m}^{2})}^{1/2}$ yields the focal-spot radius *ω*_*f*_ and hence *ω*_0_ = *λf*/(*πω*_*f*_). The cross-polarized lobe intensity was normalized to the co-polarized peak measured at the same aperture setting. Further controls are given in Supplementary Note 4, and the fitting procedure and extracted parameters in Supplementary Note 7.

### Error bars

The collecting fiber is scanned with open-loop piezo actuators, and each confocal map is acquired in the forward (trace) and backward (retrace) scan directions. The error bars in Fig. 3b and Supplementary Fig. 7 give the spread between the quantities extracted independently from the two scan directions and indicate the reproducibility of the normalized lobe intensities.

## Supplementary information

Supplementary Information

Transparent Peer Review file

## Data Availability

The datasets generated and analyzed during the current study are publicly available at the following link: https://tudatalib.ulb.tu-darmstadt.de/handle/tudatalib/5543.

## References

[1] Bliokh, K. Y., Rodríguez-Fortuño, F. J., Nori, F. & Zayats, A. V. Spin–orbit interactions of light. *Nat. Photonics* **9**, 796–808 (2015).

[2] Shen, Y. et al. Optical vortices 30 years on: OAM manipulation from topological charge to multiple singularities. *Light.: Sci. Appl.* **8**, 90 (2019). 31645934 10.1038/s41377-019-0194-2 PMC6804826

[3] Berry, M. V. Quantal phase factors accompanying adiabatic changes. *Proc. R. Soc. Lond. A. Math. Phys. Sci.* **392**, 45–57 (1984).

[4] Vinitskiĭ, S. I., Derbov, V. L., Dubovik, V. M., Markovski, B. L. & Stepanovskiĭ, Y. P. Topological phases in quantum mechanics and polarization optics. topologicheskie fazy v kvantovoj mekhanike i polyarizatsionnoj optike. *Sov. Phys. Uspekhi* **33**, 403 (1990).

[5] Bhandari, R. Polarization of light and topological phases. *Phys. Rep.* **281**, 1–64 (1997).

[6] Bliokh, K. Y., Niv, A., Kleiner, V. & Hasman, E. Geometrodynamics of spinning light. *Nat. Photonics* **2**, 748–753 (2008).

[7] Hosten, O. & Kwiat, P. Observation of the spin hall effect of light via weak measurements. *Science* **319**, 787–790 (2008). 18187623 10.1126/science.1152697

[8] Zhao, Y., Edgar, J. S., Jeffries, G. D., McGloin, D. & Chiu, D. T. Spin-to-orbital angular momentum conversion in a strongly focused optical beam. *Phys. Rev. Lett.* **99**, 073901 (2007). 17930896 10.1103/PhysRevLett.99.073901

[9] Bliokh, K. Y. et al. Spin-to-orbital angular momentum conversion in focusing, scattering, and imaging systems. *Opt. Express* **19**, 26132–26149 (2011). 22274201 10.1364/OE.19.026132

[10] Liu, W. et al. Circularly polarized states spawning from bound states in the continuum. *Phys. Rev. Lett.* **123**, 116104 (2019). 31573246 10.1103/PhysRevLett.123.116104

[11] Zeng, Y., Hu, G., Liu, K., Tang, Z. & Qiu, C.-W. Dynamics of topological polarization singularity in momentum space. *Phys. Rev. Lett.* **127**, 176101 (2021). 34739271 10.1103/PhysRevLett.127.176101

[12] Qin, H. et al. Arbitrarily polarized bound states in the continuum with twisted photonic crystal slabs. *Light Sci. Appl.* **12**, 66 (2023). 36878927 10.1038/s41377-023-01090-w PMC9988870

[13] Shen, Y. et al. Optical skyrmions and other topological quasiparticles of light. *Nat. Photonics* **18**, 15–25 (2024).

[14] Hakobyan, V. & Brasselet, E. Q-plates: From optical vortices to optical skyrmions. *Phys. Rev. Lett.* **134**, 083802 (2025). 40085861 10.1103/PhysRevLett.134.083802

[15] Onoda, M., Murakami, S. & Nagaosa, N. Hall effect of light. *Phys. Rev. Lett.* **93**, 083901 (2004). 15447185 10.1103/PhysRevLett.93.083901

[16] Bliokh, K. Y. & Aiello, A. Goos–Hänchen and Imbert–Fedorov beam shifts: an overview. *J. Opt.* **15**, 014001 (2013).

[17] Töppel, F., Ornigotti, M. & Aiello, A. Goos–Hänchen and Imbert–Fedorov shifts from a quantum-mechanical perspective. *New J. Phys.* **15**, 113059 (2013).

[18] Ling, X. et al. Recent advances in the spin Hall effect of light. *Rep. Prog. Phys.* **80**, 066401 (2017). 28357995 10.1088/1361-6633/aa5397

[19] Kim, M. et al. Spin hall effect of light: from fundamentals to recent advancements. *Laser Photonics Rev.* **17**, 2200046 (2023).

[20] Puentes, G. Spin-orbit interactions of light: Fundamentals and emergent applications. *J. Eur. Opt. Soc. -Rapid Publ.* **20**, 16 (2024).

[21] Imbert, C. Calculation and experimental proof of the transverse shift induced by total internal reflection of a circularly polarized light beam. *Phys. Rev. D.* **5**, 787 (1972).

[22] Goos, F. & Hänchen, H. Ein neuer und fundamentaler Versuch zur Totalreflexion. *Ann. der Phys.* **436**, 333–346 (1947).

[23] Bliokh, K. Y. & Bliokh, Y. P. Conservation of angular momentum, transverse shift, and spin hall effect in reflection and refraction of an electromagnetic wave packet. *Phys. Rev. Lett.* **96**, 073903 (2006). 16606091 10.1103/PhysRevLett.96.073903

[24] Aiello, A. & Woerdman, J. P. Role of beam propagation in Goos–Hänchen and Imbert–Fedorov shifts. *Opt. Lett.* **33**, 1437–1439 (2008). 18594657 10.1364/ol.33.001437

[25] Aiello, A. Goos–Hänchen and Imbert–Fedorov shifts: a novel perspective. *N. J. Phys.* **14**, 013058 (2012).

[26] Bliokh, K. Y. et al. Spin-Hall effect and circular birefringence of a uniaxial crystal plate. *Optica* **3**, 1039–1047 (2016).

[27] Takayama, O. & Puentes, G. Enhanced spin Hall effect of light by transmission in a polymer. *Opt. Lett.* **43**, 1343–1346 (2018). 29543287 10.1364/OL.43.001343

[28] Korger, J. et al. Observation of the geometric spin Hall effect of light. *Phys. Rev. Lett.* **112**, 113902 (2014). 24702371 10.1103/PhysRevLett.112.113902

[29] Bliokh, K., Prajapati, C., Samlan, C., Viswanathan, N. K. & Nori, F. Spin-Hall effect of light at a tilted polarizer. *Opt. Lett.* **44**, 4781–4784 (2019). 31568441 10.1364/OL.44.004781

[30] Modak, N., Das, S., Bordoloi, P. & Ghosh, N. Tunable giant two-dimensional optical beam shift from a tilted linear polarizer. *Phys. Rev. A* **105**, 033713 (2022).

[31] Takayama, O., Sukham, J., Malureanu, R., Lavrinenko, A. V. & Puentes, G. Photonic spin hall effect in hyperbolic metamaterials at visible wavelengths. *Opt. Lett.* **43**, 4602–4605 (2018). 30272693 10.1364/OL.43.004602

[32] Ritchie, N., Story, J. G. & Hulet, R. G. Realization of a measurement of a “weak value”. *Phys. Rev. Lett.* **66**, 1107 (1991). 10043997 10.1103/PhysRevLett.66.1107

[33] Dressel, J., Malik, M., Miatto, F. M., Jordan, A. N. & Boyd, R. W. Colloquium: understanding quantum weak values: basics and applications. *Rev. Mod. Phys.* **86**, 307–316 (2014).

[34] Götte, J. B. & Dennis, M. R. Generalized shifts and weak values for polarization components of reflected light beams. *N. J. Phys.* **14**, 073016 (2012).

[35] Choi, J. et al. Experimental observation of spin Hall effect of light using compact weak measurements. *Nanophotonics* **13**, 3877–3882 (2024). 39633730 10.1515/nanoph-2024-0217 PMC11465994

[36] Lee, J. et al. Real-time observation of the spin Hall effect of light using metasurface-enabled single-shot weak measurements. *Nat. Commun.* **16**, 2699 (2025). 40108119 10.1038/s41467-025-56728-7 PMC11923185

[37] Benelajla, M., Kammann, E., Urbaszek, B. & Karrai, K. Physical origins of extreme cross-polarization extinction in confocal microscopy. *Phys. Rev. X* **11**, 021007 (2021).

[38] Steindl, P. et al. Cross-polarization-extinction enhancement and spin-orbit coupling of light for quantum-dot cavity quantum electrodynamics spectroscopy. *Phys. Rev. Appl.* **19**, 064082 (2023).

[39] Tomm, N. et al. A bright and fast source of coherent single photons. *Nat. Nanotechnol.* **16**, 399–403 (2021). 33510454 10.1038/s41565-020-00831-x

[40] Kuhlmann, A. V. et al. A dark-field microscope for background-free detection of resonance fluorescence from single semiconductor quantum dots operating in a set-and-forget mode. *Rev. Sci. Instrum.* **84**, 073905 (2013). 23902082 10.1063/1.4813879

[41] Shree, S., Paradisanos, I., Marie, X., Robert, C. & Urbaszek, B. Guide to optical spectroscopy of layered semiconductors. *Nat. Rev. Phys.* **3**, 39–54 (2021).

[42] Nick Vamivakas, A., Zhao, Y., Lu, C.-Y. & Atatüre, M. Spin-resolved quantum-dot resonance fluorescence. *Nat. Phys.* **5**, 198–202 (2009). 10.1038/nature09359 20844531

[43] Marrucci, L., Manzo, C. & Paparo, D. Optical spin-to-orbital angular momentum conversion in inhomogeneous anisotropic media. *Phys. Rev. Lett.* **96**, 163905 (2006). 16712234 10.1103/PhysRevLett.96.163905

[44] Devlin, R. C., Ambrosio, A., Rubin, N. A., Mueller, J. P. B. & Capasso, F. Arbitrary spin-to-orbital angular momentum conversion of light. *Science* **358**, 896–901 (2017). 29097490 10.1126/science.aao5392

[45] Ferrand, P., Baroni, A., Allain, M. & Chamard, V. Quantitative imaging of anisotropic material properties with vectorial ptychography. *Opt. Lett.* **43**, 763–766 (2018). 29443988 10.1364/OL.43.000763

