# Supplementary material for: Real-space spin-orbit interaction from quarter-wave plate nonuniformity
Source: Nat Commun. 2026 Sep 9;17:9653. doi: 10.1038/s41467-026-77581-2 (PMC13558598; doi:10.1038/s41467-026-77581-2)
Supplement: Supplementary file 1 [file 41467_2026_77581_MOESM1_ESM.pdf]

Supplementary Materials for "*Real-space spin–orbit interaction from quarter-wave plate nonuniformity*"

Wenze Lan‡,[1,*] Anton Lögl‡,[1] Peng Fu‡,[2] Duy Anh Tran,[1] Meryem Benelajla,[3] Clemens Schäfermeier,[4] Khaled Karrai,[4] and Bernhard Urbaszek[1,†]

[1]*Institute for Condensed Matter Physics, Technische Universität Darmstadt, Hochschulstr. 6, 64289 Darmstadt, Germany*
[2]*Laboratoire Photonique Numérique et Nanosciences (LP2N), Université de Bordeaux, Institut d'Optique Graduate School, CNRS, 33400 Talence, France*
[3]*eyeo BV, High Tech Campus 41, 5656 AE Eindhoven, The Netherlands*
[4]*attocube systems GmbH, Eglfinger Weg 2, 85540 Haar, Germany*

CONTENTS

Supplementary Note 1. Plane-wave Jones-matrix analysis for different geometries 3
A. Polarizer–quarter-wave plate–analyzer 3
B. Polarizer–analyzer 3
C. Polarizer–half-wave plate–analyzer 4
D. Effect of Φ_0 on the extinction ratio 5
E. Modal transformation and extinction ratio curve for "polarizer–half-wave plate (HWP)–analyzer" geometry 6

Supplementary Note 2. First-order angular Jones-matrix theory for a finite Gaussian beam 7
A. Geometry, angular spectrum, and QWP convention 7
B. Propagation from the QWP to the collecting objective 8
C. Fourier transform by the objective 9
D. Single-mode-fiber collection 9

Supplementary Note 3. Complete first-order theory including real-space QWP gradients 11
A. First-order Jones expansion of a real QWP 11
B. Conversion of spatial gradients into angular-spectrum terms 11
C. Collected field and scalar cross-polarized amplitude 12
D. Geometry and peak intensity of the collected first-order component 13
E. Experimental consequence: beam-size scaling at fixed fiber mode 13
F. Magnitude estimates 14

Supplementary Note 4. Experimental controls and scaling analyses 15
A. Direct imaging without single-mode-fiber detection 15
B. Comparison between different QWPs and wavelengths 16
C. Beam-size dependence 17
D. Tilt-angle dependence 19
E. Anti-crossing behavior of the mode peak 20
F. Extraction of lobe orientation 21

Supplementary Note 5. Full vectorial non-paraxial calculation for an ideal QWP 22
A. Motivation 22
B. Vectorial mapping by the collimating objective 22
C. Incident field: angular spectrum of the fiber mode 23
D. Analytical evaluation of the transformed pupil field 23
E. Field on the outgoing reference sphere 24
F. Richards–Wolf integral and radial reduction 24
G. Spatially uniform polarizer–QWP–analyzer model 25
H. Comparison with experiment 27
1. No transverse first-order mode is generated 27
2. The longitudinal first-order mode is negligible 27
I. Conclusion 27

Supplementary Note 6. Mach–Zehnder interferometric characterization 28

Supplementary Note 7. Fitting procedure and extracted parameters 30

Supplementary Note 8. Vendor specifications and angle-of-incidence retardance data 32

Supplementary References 33

* wenze.lan@pkm.tu-darmstadt.de
† bernhard.urbaszek@pkm.tu-darmstadt.de

Supplementary Note 1. PLANE-WAVE JONES-MATRIX ANALYSIS FOR DIFFERENT GEOMETRIES

This note establishes the plane-wave polarization baseline used in the rest of the Supplementary Materials. The purpose is to separate ordinary Jones-matrix compensation of polarization leakage from spatial effects that appear only for a finite beam. We first define the optical elements, then compare three geometries: polarizer–quarter-wave plate–analyzer, polarizer–analyzer, and polarizer–half-wave plate–analyzer.

A linear polarizer aligned along the x axis with finite leakage is written as

$$\bar{L}\bar{P}_{p_0} = \begin{pmatrix} a & 0 \\ ib & 0 \end{pmatrix}, \tag{1}$$

where $a^2 + b^2 = 1$, $a, b \in \mathbb{R}$, and $a^2 \gg b^2$. The rotation matrix is

$$\bar{R}(\varphi) = \begin{pmatrix} \cos\varphi & -\sin\varphi \\ \sin\varphi & \cos\varphi \end{pmatrix}. \tag{2}$$

A polarizer rotated by β and an analyzer rotated by α are therefore

$$\bar{P}(\beta) = \bar{R}(\beta)\bar{L}\bar{P}_{p_0}\bar{R}(-\beta), \qquad \bar{A}(\alpha) = \bar{R}(\alpha)\bar{L}\bar{A}_{p_0}\bar{R}(-\alpha), \tag{3}$$

where the analyzer leakage matrix $\bar{L}\bar{A}_{p_0}$ has the same form as $\bar{L}\bar{P}_{p_0}$; for simplicity we assume identical leakage parameters (a, b) for polarizer and analyzer. The zero-order half-wave plate and quarter-wave plate are written as

$$\bar{H} = -i\begin{pmatrix} 1 & 0 \\ 0 & -1 \end{pmatrix}, \qquad \bar{Q} = e^{-i\pi/4}\begin{pmatrix} 1 & 0 \\ 0 & i \end{pmatrix}. \tag{4}$$

Unless otherwise stated, the input Jones vector is

$$\boldsymbol{E}_0 = \begin{pmatrix} 1 \\ 0 \end{pmatrix}. \tag{5}$$

A. Polarizer–quarter-wave plate–analyzer

The input field is rotated by β before entering the polarizer,

$$\boldsymbol{E}(\beta) = \bar{R}(\beta)\boldsymbol{E}_0. \tag{6}$$

After the polarizer, QWP, and analyzer, the output field is

$$\boldsymbol{E} = \bar{A}(\alpha)\bar{Q}\bar{P}(\beta)\bar{R}(\beta)\boldsymbol{E}_0. \tag{7}$$

A direct multiplication gives the detected intensity

$$I \propto \boldsymbol{E}^* \cdot \boldsymbol{E} = \cos^2\beta\,(a\cos\alpha - b\sin\alpha)^2 + \sin^2\beta\,(b\cos\alpha - a\sin\alpha)^2. \tag{8}$$

Equation (8) admits complete cancellation by suitable choices of α and β. For example, fixing $\beta = 0$ gives the extinction condition

$$\boxed{\cos^2\alpha = b^2.} \tag{9}$$

Thus, at the plane-wave level, inserting a QWP gives one additional degree of freedom that can compensate the leakage field.

B. Polarizer–analyzer

Without an additional polarization element,

$$\boldsymbol{E} = \bar{A}(\alpha)\bar{P}(\beta)\bar{R}(\beta)\boldsymbol{E}_0. \tag{10}$$

The detected intensity is

$$I \propto \boldsymbol{E}^* \cdot \boldsymbol{E} = a^2 \cos^2(\alpha - \beta) + b^2 \sin^2(\alpha - \beta). \tag{11}$$

Perfect extinction would require

$$\cos^2(\alpha - \beta) = \frac{-b^2}{a^2 - b^2}, \tag{12}$$

which has no physical solution because the left-hand side is non-negative. Therefore, a polarizer–analyzer pair alone cannot eliminate the leakage field. For $\beta = 0$ and $\alpha = \pi/2$, the residual intensity is

$$I = b^2. \tag{13}$$

C. Polarizer–half-wave plate–analyzer

With a half-wave plate inserted between polarizer and analyzer,

$$\boldsymbol{E} = \bar{A}(\alpha)\bar{H}\bar{P}(\beta)\bar{R}(\beta)\boldsymbol{E}_0. \tag{14}$$

The detected intensity becomes

$$I \propto \boldsymbol{E}^* \cdot \boldsymbol{E} = a^2 \cos^2(\alpha + \beta) + b^2 \sin^2(\alpha + \beta). \tag{15}$$

Again, no choice of α and β makes the intensity vanish. For $\beta = 0$ and $\alpha = \pi/2$, one obtains

$$I = b^2. \tag{16}$$

The plane-wave result therefore identifies the QWP as the element that enables extinction of the average leakage. Note that this model assumes identical leakage parameters (a, b) for polarizer and analyzer; relaxing this assumption makes the HWP residual weakly angle dependent, consistent with the modest improvement over the polarizer–analyzer pair observed experimentally, but it does not create a cancellation condition. The remaining sections address why, for a finite beam and a real wave plate, a spatially structured residual field can remain even when the plane-wave extinction condition is optimized.

D. Effect of Φ_0 on the extinction ratio

In the experiment, the extinction is optimized by adjusting the polarizer and analyzer angles over a finite angular range. To estimate the effect of finite angular optimization, we perform a plane-wave Jones-matrix calculation in which the polarizer and analyzer angles are searched over prescribed numerical tuning windows with a prescribed sampling step.

The resulting optimized extinction ratio is shown in Supplementary Fig. 1. For a representative search window of $\pm 0.2°$ and an angular sampling step of $0.005°$, the optimized extinction ratio falls in the 10^7–10^8 range [Supplementary Fig. 1b], even for a retardance Φ_0 close to the ideal quarter-wave value. A smaller window imposes a stronger numerical limitation [Supplementary Fig. 1a], whereas a larger window gives access to additional cancellation conditions but shows oscillations associated with the discrete angular sampling [Supplementary Fig. 1c]. Retardance detuning, for example from a mismatch between the laser and the QWP design wavelength, further reduces the achievable extinction. These values are not a physical bound: in the exact plane-wave limit the condition $\cos^2\alpha = b^2$ of Sec. Supplementary Note 1 A cancels the mean leakage completely, so the finite extinction obtained here follows from the finite search window and the discrete angular step alone.

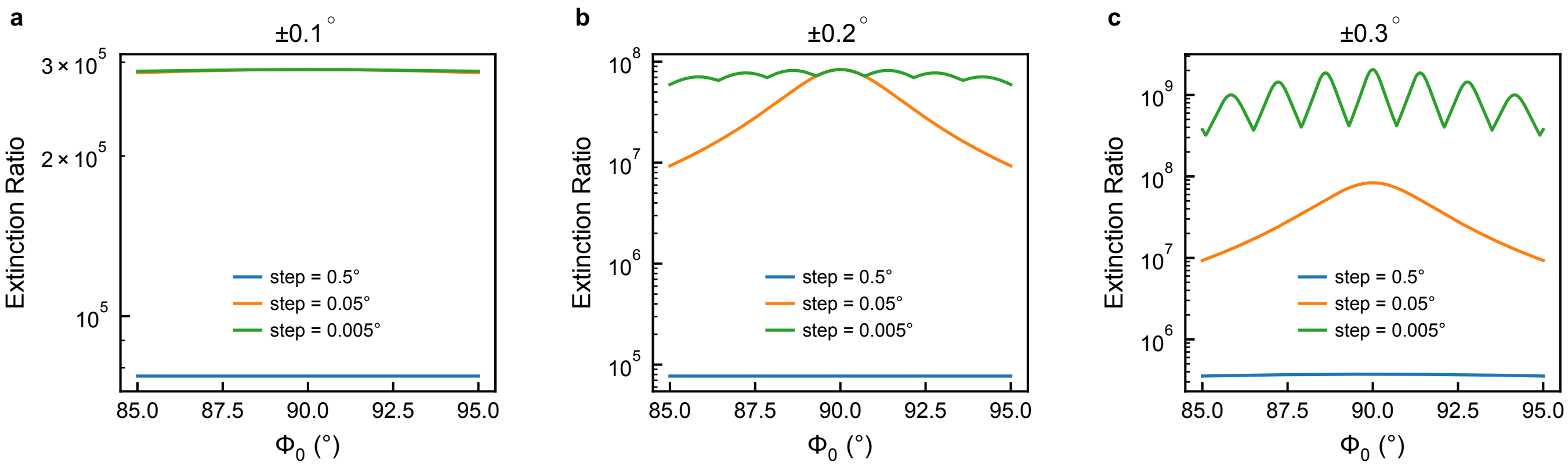

Supplementary Figure 1. **Finite-search Jones-matrix estimate of the optimized extinction ratio.** Optimized extinction ratio calculated as a function of the QWP retardance Φ_0 for prescribed angular search windows of the polarizer and analyzer: **a**, $\pm 0.1°$; **b**, $\pm 0.2°$; and **c**, $\pm 0.3°$. Within each window, the polarizer and analyzer angles are searched on a discrete numerical grid, with angular step size $\Delta\alpha = \Delta\beta$ indicated in the legend. The window and the step size are parameters of the numerical search and are not intended to reproduce the experimental optimization procedure. Finite angular optimization within a restricted search range leads to an extinction scale of 10^7–10^8 for retardances near the quarter-wave condition; the oscillations arise from the discrete angular sampling.

E. Modal transformation and extinction ratio curve for "polarizer–half-wave plate (HWP)–analyzer" geometry

In the *polarizer–half-wave plate (HWP)–analyzer* geometry, we performed the same co- and cross-polarized measurement [Supplementary Fig. 2]. The extinction ratio remains limited to $\sim 10^6$, well below the $\sim 10^7$ reached with the quartz zero-order QWP, and both confocal maps retain a Gaussian-like mode profile, without the first-order derivative-mode splitting observed with a QWP.

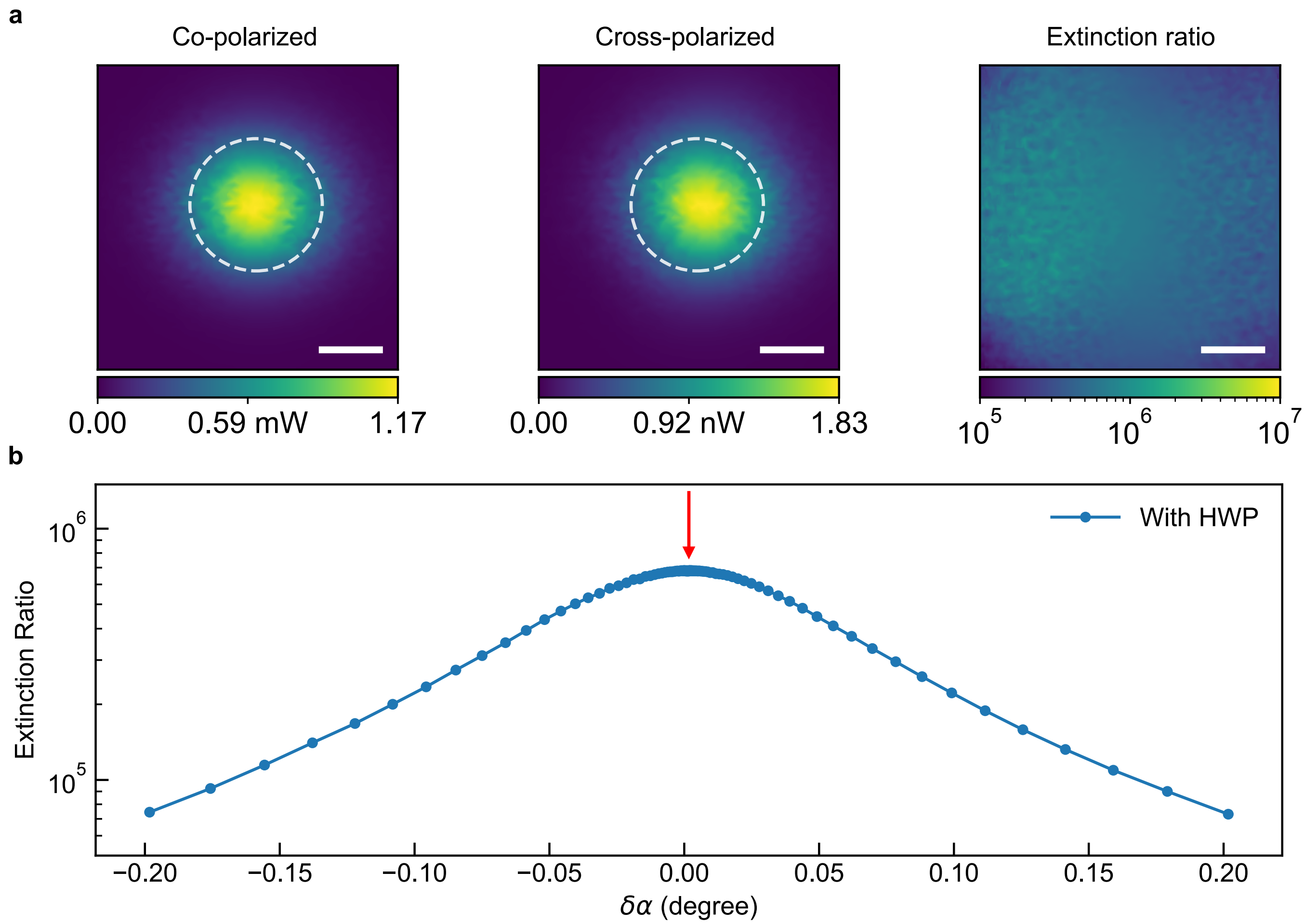

Supplementary Figure 2. **Half-wave-plate control experiment.** **a**, Cross-polarized confocal modal map measured in the polarizer–half-wave-plate–analyzer geometry. **b**, Extinction ratio as a function of analyzer angle for the same configuration. The red arrow marks the analyzer angle used to acquire the modal map in **a**. This control measurement illustrates the different extinction behavior obtained when the QWP is replaced by a zero-order HWP. Scale bar: 2.5 μm.

Supplementary Note 2. FIRST-ORDER ANGULAR JONES-MATRIX THEORY FOR A FINITE GAUSSIAN BEAM

This note derives the field collected by a scanning single-mode fiber (SMF) when a finite Gaussian beam passes through a thin, laterally uniform QWP whose Jones matrix depends on propagation direction. This is the angular-only first-order theory: different plane-wave components in the beam sample slightly different polarization transformations, while the plate itself has no real-space gradients.

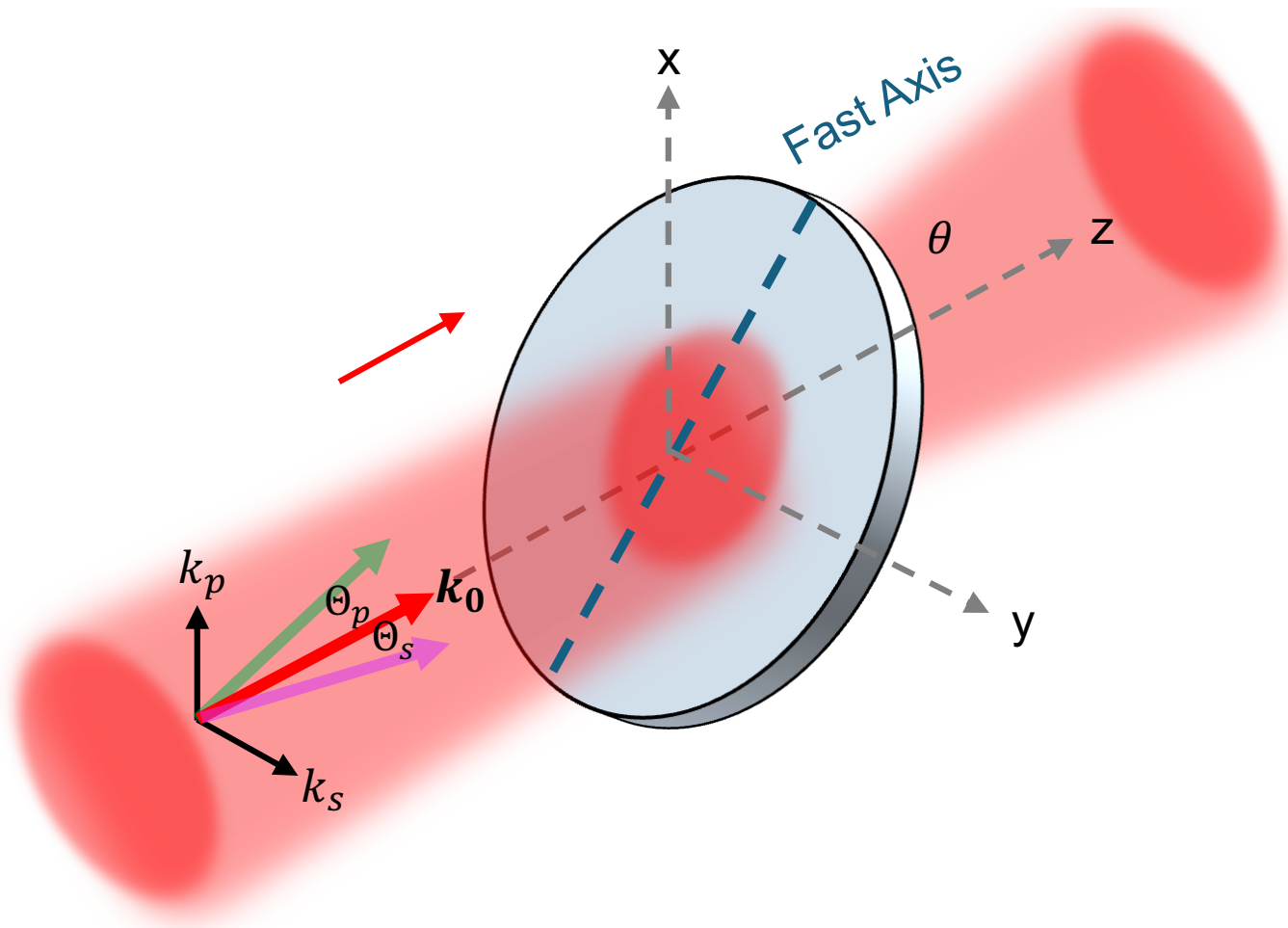

Supplementary Figure 3. **Schematics of a finite Gaussian beam incident on a laterally uniform QWP.** The incident finite beam is decomposed into plane-wave components labelled by the dimensionless transverse angular coordinates $\Theta_p = k_p/k_0$ and $\Theta_s = k_s/k_0$, measured relative to the central propagation direction along the fast (p) and slow (s) axes of the plate. The central component propagates along z with wave number k_0. The angle θ denotes the angle between the optic axis of the plate and the wavevector of the plane-wave component considered; for the central component at exact normal incidence, $\theta = 90^\circ$. This geometry is used to derive the first-order momentum-domain Jones-matrix expansion in Supplementary Note 2.

A. Geometry, angular spectrum, and QWP convention

We use (p, s) for transverse coordinates in the collimated QWP plane and (x, y) for transverse coordinates in the focal plane of the collecting objective. The transverse wave vector is

$$\boldsymbol{k}_\perp = (k_p, k_s), \qquad \Theta_p \equiv \frac{k_p}{k_0}, \qquad \Theta_s \equiv \frac{k_s}{k_0}, \qquad k_0 = \frac{2\pi}{\lambda}. \tag{17}$$

The Gaussian angular spectrum at the QWP plane is taken as

$$\hat{\boldsymbol{\mathcal{E}}}(k_p, k_s, 0) = \frac{\omega_0^2}{4\pi} \exp\left[-\frac{\omega_0^2(k_p^2 + k_s^2)}{4}\right] \boldsymbol{E}_0 = \frac{\omega_0^2}{4\pi} \exp\left[-\frac{\Theta_p^2 + \Theta_s^2}{\theta_0^2}\right] \boldsymbol{E}_0, \tag{18}$$

where ω_0 is the collimated Gaussian radius at the $1/e$ field level (equivalently, the $1/e^2$ intensity radius quoted in the Methods of the main text) and $\theta_0 = 2/(k_0\omega_0) = \lambda/(\pi\omega_0) \approx 1.0 \times 10^{-4}$ rad is the $1/e$ divergence half-angle of the collimated beam. Throughout the Supplementary Materials, (Θ_p, Θ_s) denote dimensionless transverse direction cosines and (p, s) transverse coordinates in the QWP plane.

For the central plane-wave component, the QWP is described by

$$\bar{Q}_0 = \begin{pmatrix} e^{-i\Phi_0/2} & 0 \\ 0 & e^{+i\Phi_0/2} \end{pmatrix}, \qquad \Phi_0 \simeq \frac{\pi}{2}. \tag{19}$$

The symmetric $\pm\Phi_0/2$ convention removes the common phase; only the retardance difference Φ_0 is physically relevant.

A finite beam contains plane-wave components with $(\Theta_p, \Theta_s) \neq (0, 0)$. For a thin positive uniaxial birefringent plate, the first-order angular response can be written following the established momentum-domain Jones-matrix framework

as [2–5]

$$\bar{Q}(\Theta_p, \Theta_s) \simeq \begin{pmatrix} e^{-i\Phi_0/2}(1+\Theta_p\mathcal{X}_e) & e^{-i\Phi_0/2}\Theta_s\mathcal{Y}_e \\ -e^{+i\Phi_0/2}\Theta_s\mathcal{Y}_o & e^{+i\Phi_0/2}(1+\Theta_p\mathcal{X}_o) \end{pmatrix}, \tag{20}$$

with

$$\mathcal{X}_{e,o} = \mp\frac{i}{2}\frac{d\Phi}{d\theta}, \qquad \mathcal{Y}_{e,o} = [1-\exp(\pm i\Phi_0)]\cot\theta, \tag{21}$$

where $\Phi(\theta)$ is the retardance of the plane-wave component whose wavevector makes an angle θ with the optic axis of the plate (see Supplementary Fig. 3), and $\Phi_0 \equiv \Phi(90°) \simeq \pi/2$; for a standard wave plate at exact normal incidence, $\theta = 90°$ for the central component. The symmetry constraint follows directly from these expressions: at exact normal incidence, $\cot\theta = 0$ and $\Phi(\theta)$ is extremal, so that $d\Phi/d\theta = 0$. Consequently $\mathcal{X}_{e,o} = \mathcal{Y}_{e,o} = 0$ and $\bar{Q}_1 = \bar{Q}_2 = 0$: the intrinsic first-order angular spin–orbit response of an ideal QWP vanishes at normal incidence. A nonzero effective first-order angular coefficient therefore requires residual macroscopic symmetry breaking, such as a small plate tilt; finite beam divergence is retained in the angular-spectrum description but does not by itself remove this first-order normal-incidence symmetry. Equivalently,

$$\bar{Q}(\Theta_p, \Theta_s) = \bar{Q}_0 + \Theta_p\bar{Q}_1 + \Theta_s\bar{Q}_2 + O(\Theta_p^2, \Theta_s^2, \Theta_p\Theta_s), \tag{22}$$

where

$$\bar{Q}_1 = \begin{pmatrix} e^{-i\Phi_0/2}\mathcal{X}_e & 0 \\ 0 & e^{+i\Phi_0/2}\mathcal{X}_o \end{pmatrix}, \qquad \bar{Q}_2 = \begin{pmatrix} 0 & e^{-i\Phi_0/2}\mathcal{Y}_e \\ -e^{+i\Phi_0/2}\mathcal{Y}_o & 0 \end{pmatrix}. \tag{23}$$

The matrices $\bar{Q}_1$ and $\bar{Q}_2$ describe the intrinsic angular spin-orbit response of an otherwise uniform QWP.

B. Propagation from the QWP to the collecting objective

Under the paraxial approximation,

$$k_z = k_0\sqrt{1-\frac{k_p^2+k_s^2}{k_0^2}} \simeq k_0 - \frac{k_p^2+k_s^2}{2k_0}. \tag{24}$$

After the QWP, the angular spectrum is

$$\hat{\boldsymbol{\mathcal{E}}}'(k_p, k_s, 0) = \frac{\omega_0^2}{4\pi}\exp\left[-\frac{\omega_0^2(k_p^2+k_s^2)}{4}\right]\left(\bar{Q}_0 + \frac{k_p}{k_0}\bar{Q}_1 + \frac{k_s}{k_0}\bar{Q}_2\right)\boldsymbol{E}_0. \tag{25}$$

Propagation over a distance z multiplies each component by

$$\exp\left[i\left(k_0 - \frac{k_p^2+k_s^2}{2k_0}\right)z\right]. \tag{26}$$

It is useful to define the Rayleigh range and complex beam parameter

$$\ell = \frac{k_0\omega_0^2}{2}, \qquad \omega_z^2 = \omega_0^2\left(1+i\frac{z}{\ell}\right), \qquad \ell_z = \ell + iz. \tag{27}$$

The propagated angular spectrum becomes

$$\hat{\boldsymbol{\mathcal{E}}}'(k_p, k_s, z) = \frac{\omega_0^2}{4\pi}e^{ik_0 z}\exp\left[-\frac{\omega_z^2(k_p^2+k_s^2)}{4}\right]\left(\bar{Q}_0 + \frac{k_p}{k_0}\bar{Q}_1 + \frac{k_s}{k_0}\bar{Q}_2\right)\boldsymbol{E}_0. \tag{28}$$

Taking the inverse Fourier transform gives the spatial field at the lens-input plane:

$$\boxed{\boldsymbol{E}(p, s, z) = \frac{\omega_0^2}{\omega_z^2}e^{ik_0 z - (p^2+s^2)/\omega_z^2}\left(\bar{Q}_0 + \frac{ip}{\ell_z}\bar{Q}_1 + \frac{is}{\ell_z}\bar{Q}_2\right)\boldsymbol{E}_0.} \tag{29}$$

The zeroth-order term remains an even Gaussian mode, whereas the first-order angular terms become odd spatial modes proportional to p and s.

C. Fourier transform by the objective

An ideal thin lens of focal length f performs a Fourier transform from the lens-input plane to the focal plane. If z is measured to the back focal plane of the lens,

$$\boldsymbol{E}(x,y,f) = -\frac{i}{\lambda f}\iint \boldsymbol{E}(p,s,z)\exp\left[-i\frac{k_0}{f}(xp+ys)\right]dp\,ds. \tag{30}$$

Using $\lambda f = \pi\omega_0\omega_f$ and

$$\omega_f = \frac{2f}{k_0\omega_0} = \frac{\lambda f}{\pi\omega_0}, \tag{31}$$

one obtains

$$\boxed{\boldsymbol{E}(x,y,f) = -i\frac{\omega_0}{\omega_f}\exp\left[ik_0z - \left(1+i\frac{z}{\ell}\right)\frac{x^2+y^2}{\omega_f^2}\right]\left(\bar{Q}_0 + \frac{x}{f}\bar{Q}_1 + \frac{y}{f}\bar{Q}_2\right)\boldsymbol{E}_0.} \tag{32}$$

After the polarizer and analyzer are included, Eq. (32) becomes

$$\boldsymbol{E}(x,y,f) = \mathcal{C}_f\exp\left[-\left(1+i\frac{z}{\ell}\right)\frac{x^2+y^2}{\omega_f^2}\right]\bar{A}(\alpha)\left(\bar{Q}_0 + \frac{x}{f}\bar{Q}_1 + \frac{y}{f}\bar{Q}_2\right)\bar{P}(\beta)\boldsymbol{E}_0, \tag{33}$$

where $\mathcal{C}_f$ contains only global phase and normalization factors.

D. Single-mode-fiber collection

The scanning SMF is represented by a Gaussian collection mode with mode-field radius ω_m and center position (x_0, y_0),

$$\mathcal{F}(x-x_0, y-y_0) = \frac{1}{\pi\omega_m^2}\exp\left[-\frac{(x-x_0)^2+(y-y_0)^2}{\omega_m^2}\right]. \tag{34}$$

The field coupled into the SMF is obtained from the spatial overlap

$$\boldsymbol{E}_{\mathrm{SMF}}(x_0,y_0) = \iint \mathcal{F}(x-x_0,y-y_0)\,\boldsymbol{E}(x,y,f)\,dx\,dy. \tag{35}$$

For a focused Gaussian beam with field radius ω_f, and in the limit $z \ll \ell$, the first-order angular expansion before SMF collection can be written as

$$\boldsymbol{E}(x,y,f) = \mathcal{C}_f\exp\left[-\frac{x^2+y^2}{\omega_f^2}\right]\bar{A}(\alpha)\left(\bar{Q}_0 + \frac{x}{f}\bar{Q}_1 + \frac{y}{f}\bar{Q}_2\right)\bar{P}(\beta)\boldsymbol{E}_0. \tag{36}$$

Substitution into the overlap integral gives the general collected field

$$\boxed{\boldsymbol{E}_{\mathrm{SMF}}(x_0,y_0) = \mathcal{C}_{\mathrm{SMF}}\exp\left[-\frac{x_0^2+y_0^2}{\omega_f^2+\omega_m^2}\right]\bar{A}(\alpha)\left[\bar{Q}_0 + \frac{\omega_f^2}{\omega_f^2+\omega_m^2}\left(\frac{x_0}{f}\bar{Q}_1 + \frac{y_0}{f}\bar{Q}_2\right)\right]\bar{P}(\beta)\boldsymbol{E}_0.} \tag{37}$$

Here $\mathcal{C}_{\mathrm{SMF}}$ absorbs all position-independent constants, including the Gaussian-overlap normalization. The exponential factor is the overlap envelope between the focused field and the displaced SMF mode. Correspondingly, the bright-field intensity profile follows

$$I_{\mathrm{BF}}(x_0,y_0) \propto \exp\left[-\frac{2(x_0^2+y_0^2)}{\omega_f^2+\omega_m^2}\right]. \tag{38}$$

Thus, fitting the measured BF map with this expression allows ω_f to be extracted when ω_m is known. For the matched-waist case, $\omega_m = \omega_f$, Eq. (37) reduces to

$$\boxed{\boldsymbol{E}_{\mathrm{SMF}}(x_0, y_0) = \mathcal{C}_{\mathrm{SMF}} \exp\left[-\frac{x_0^2 + y_0^2}{2\omega_f^2}\right] \bar{A}(\alpha) \left(\bar{Q}_0 + \frac{x_0}{2f}\bar{Q}_1 + \frac{y_0}{2f}\bar{Q}_2\right) \bar{P}(\beta)\boldsymbol{E}_0.} \tag{39}$$

Equation (37) is the angular-only finite-beam theory after SMF collection. It predicts an odd transverse contribution with two-lobe symmetry through $\bar{Q}_1$ and $\bar{Q}_2$. For an ideal uniform QWP at exact normal incidence this first-order term vanishes, and an estimate based on residual symmetry breaking remains far below the measurement (Sec. Supplementary Note 3 F). This motivates the complete first-order description of Supplementary Note 3, in which the real-space gradients of the QWP Jones matrix are retained.

Supplementary Note 3. COMPLETE FIRST-ORDER THEORY INCLUDING REAL-SPACE QWP GRADIENTS

This note extends Supplementary Note 2 by allowing the QWP Jones response to vary slowly across the illuminated aperture. Angular dispersion and real-space Jones gradients generate the same first-order spatial sector, but they scale differently with the incident beam radius.

A. First-order Jones expansion of a real QWP

Within the thin-element approximation, the transmitted field is written as

$$\boldsymbol{E}_{\text{out}} = \hat{\mathcal{Q}}\boldsymbol{E}_{\text{in}}, \tag{40}$$

where the Jones operator is expanded about the beam center and the central propagation direction as

$$\boxed{\hat{\mathcal{Q}} = \bar{Q}_0 + \frac{\hat{k}_p}{k_0}\bar{Q}_1 + \frac{\hat{k}_s}{k_0}\bar{Q}_2 + \hat{p}\,\bar{Q}_3 + \hat{s}\,\bar{Q}_4 + \mathcal{O}(2).} \tag{41}$$

Here p and s denote the transverse coordinates along the local fast- and slow-axis directions, and $\hat{k}_p$ and $\hat{k}_s$ are the corresponding transverse-wave-vector operators. The angular coefficients are

$$\bar{Q}_1 = \left.\frac{\partial \bar{Q}}{\partial \Theta_p}\right|_0, \qquad \bar{Q}_2 = \left.\frac{\partial \bar{Q}}{\partial \Theta_s}\right|_0, \qquad \Theta_{p,s} = \frac{k_{p,s}}{k_0}, \tag{42}$$

and the real-space gradients are

$$\bar{Q}_3 = \left.\frac{\partial \bar{Q}}{\partial p}\right|_0, \qquad \bar{Q}_4 = \left.\frac{\partial \bar{Q}}{\partial s}\right|_0. \tag{43}$$

The matrices $\bar{Q}_3$ and $\bar{Q}_4$ have units of inverse length. They may contain contributions from spatial variations of the retardance, fast-axis direction, thickness, stress, or coating response. For example,

$$\bar{Q}_{3,4} = \frac{\partial \bar{Q}}{\partial \Phi}\,\partial_{p,s}\Phi + \frac{\partial \bar{Q}}{\partial \gamma}\,\partial_{p,s}\gamma + \cdots, \tag{44}$$

where Φ and γ are the local retardance and fast-axis angle.

B. Conversion of spatial gradients into angular-spectrum terms

Using the Fourier convention for which multiplication by p becomes $i\partial_{k_p}$, Eq. (41) gives

$$\begin{aligned}\hat{\boldsymbol{E}}_{\text{out}}(k_p,k_s) = &\left(\bar{Q}_0 + \frac{k_p}{k_0}\bar{Q}_1 + \frac{k_s}{k_0}\bar{Q}_2\right)\hat{\boldsymbol{E}}_{\text{in}}(k_p,k_s) \\ &+ i\bar{Q}_3\frac{\partial \hat{\boldsymbol{E}}_{\text{in}}}{\partial k_p} + i\bar{Q}_4\frac{\partial \hat{\boldsymbol{E}}_{\text{in}}}{\partial k_s}.\end{aligned} \tag{45}$$

For the Gaussian spectrum of Eq. (18),

$$i\frac{\partial}{\partial k_p}\exp\left[-\frac{\omega_0^2(k_p^2+k_s^2)}{4}\right] = -\frac{i\omega_0^2 k_p}{2}\exp\left[-\frac{\omega_0^2(k_p^2+k_s^2)}{4}\right], \tag{46}$$

and similarly for k_s. The cross-polarized angular spectrum after the analyzer is therefore

$$\hat{\boldsymbol{E}}_{\times}(k_p,k_s) = \frac{\omega_0^2}{4\pi}\exp\left[-\frac{\omega_0^2(k_p^2+k_s^2)}{4}\right]\bar{A}(\alpha)\left(\bar{N}_0 + \frac{k_p}{k_0}\bar{N}_1 + \frac{k_s}{k_0}\bar{N}_2\right)\bar{P}(\beta)\boldsymbol{E}_0, \tag{47}$$

with

$$\bar{N}_0 = \bar{Q}_0, \tag{48}$$

$$\bar{N}_1 = \bar{Q}_1 - i\frac{k_0\omega_0^2}{2}\bar{Q}_3, \tag{49}$$

$$\bar{N}_2 = \bar{Q}_2 - i\frac{k_0\omega_0^2}{2}\bar{Q}_4. \tag{50}$$

Angular dispersion and real-space gradients therefore contribute to the same first-order angular-spectrum terms. Their distinction lies in the ω_0^2 weighting of the real-space contribution.

C. Collected field and scalar cross-polarized amplitude

Equation (47) has the same form as the angular-spectrum expression derived in Supplementary Note 2, with $\bar{Q}_{1,2}$ replaced by $\bar{N}_{1,2}$. For an SMF mode radius ω_m and a focused-field radius ω_f, the collected Jones field is

$$\boxed{\boldsymbol{E}_{\mathrm{SMF}}(x_0, y_0) = \mathcal{C}_{\mathrm{SMF}} \exp\left[-\frac{x_0^2+y_0^2}{\omega_f^2+\omega_m^2}\right] \bar{A}(\alpha)\left[\bar{N}_0 + \Lambda\left(\frac{x_0}{f}\bar{N}_1 + \frac{y_0}{f}\bar{N}_2\right)\right]\bar{P}(\beta)\boldsymbol{E}_0,} \tag{51}$$

where

$$\Lambda = \frac{\omega_f^2}{\omega_f^2+\omega_m^2} \tag{52}$$

is the centroid factor of the overlap between the focused Gaussian field and the displaced SMF mode. All position-independent factors are included in $\mathcal{C}_{\mathrm{SMF}}$.

Let $\boldsymbol{a}^\dagger$ denote the analyzer projection and define $\boldsymbol{e}_{\mathrm{P}} = \bar{P}(\beta)\boldsymbol{E}_0$. At the focal plane, the scalar cross-polarized field before SMF collection is

$$\boxed{\psi_\times(x, y) = \mathcal{C}_f \exp\left[-\frac{x^2+y^2}{\omega_f^2}\right](\eta_0 + G_p x + G_s y),} \tag{53}$$

where

$$\eta_0 = \boldsymbol{a}^\dagger\bar{Q}_0\boldsymbol{e}_{\mathrm{P}}, \tag{54}$$

$$G_p = \frac{1}{f}\boldsymbol{a}^\dagger\bar{Q}_1\boldsymbol{e}_{\mathrm{P}} - i\frac{k_0\omega_0^2}{2f}\boldsymbol{a}^\dagger\bar{Q}_3\boldsymbol{e}_{\mathrm{P}}, \tag{55}$$

$$G_s = \frac{1}{f}\boldsymbol{a}^\dagger\bar{Q}_2\boldsymbol{e}_{\mathrm{P}} - i\frac{k_0\omega_0^2}{2f}\boldsymbol{a}^\dagger\bar{Q}_4\boldsymbol{e}_{\mathrm{P}}. \tag{56}$$

The coefficients G_p and G_s contain the angular and real-space first-order contributions projected onto the analyzer channel.

Projecting Eq. (53) onto the displaced Gaussian SMF mode gives

$$\boxed{c(x_0, y_0) = \mathcal{C}_{\mathrm{SMF}} \exp\left[-\frac{x_0^2+y_0^2}{\omega_f^2+\omega_m^2}\right][\eta_0 + \Lambda(G_p x_0 + G_s y_0)].} \tag{57}$$

The SMF broadens the Gaussian envelope and multiplies the first-order term by Λ; it does not change the parity of the odd term.

For $\omega_m = \omega_f$, Eq. (51) becomes

$$\boxed{\boldsymbol{E}_{\mathrm{SMF}}(x_0, y_0) = \mathcal{C}_{\mathrm{SMF}} \exp\left[-\frac{x_0^2+y_0^2}{2\omega_f^2}\right] \bar{A}(\alpha)\left(\bar{N}_0 + \frac{x_0}{2f}\bar{N}_1 + \frac{y_0}{2f}\bar{N}_2\right)\bar{P}(\beta)\boldsymbol{E}_0,} \tag{58}$$

and the scalar amplitude reduces to

$$\boxed{c(x_0, y_0) = \mathcal{C}_{\mathrm{SMF}} \exp\left[-\frac{x_0^2 + y_0^2}{2\omega_f^2}\right] \left[\eta_0 + \frac{1}{2}\left(G_p x_0 + G_s y_0\right)\right].} \tag{59}$$

Equation (57) is the collected-field model used in Eq. (6) of the main text. The even coefficient η_0 describes the residual on-axis leakage, whereas G_p and G_s describe the first-order component. In the laboratory frame, (G_p, G_s) is rotated into (G_x, G_y) through the QWP fast-axis angle. The coefficients returned by the confocal-map fit are the collected coefficients $\boldsymbol{G}^{\mathrm{fit}} = \Lambda \boldsymbol{G}$.

D. Geometry and peak intensity of the collected first-order component

The expressions in this subsection refer to the pure first-order component, $\eta_0 = 0$. When the even leakage is retained, the peak positions and lobe asymmetry are obtained from the full field in Eq. (57).

For the generally complex vector $\boldsymbol{G} = (G_p, G_s)$, define

$$\bar{M}_G = \begin{pmatrix} |G_p|^2 & \mathrm{Re}(G_p G_s^*) \\ \mathrm{Re}(G_p G_s^*) & |G_s|^2 \end{pmatrix}, \qquad G_{\mathrm{pk}}^2 = \lambda_{\max}(\bar{M}_G). \tag{60}$$

The principal eigenvector of $\bar{M}_G$ gives the axis of maximum first-order intensity. Along this axis,

$$|c(r)|^2 \propto G_{\mathrm{pk}}^2 r^2 \exp\left[-\frac{2r^2}{\omega_f^2 + \omega_m^2}\right]. \tag{61}$$

The two maxima are located at

$$r_{\max} = \sqrt{\frac{\omega_f^2 + \omega_m^2}{2}}. \tag{62}$$

If G_p and G_s have a common phase, then $G_{\mathrm{pk}}^2 = |G_p|^2 + |G_s|^2$.

Up to position-independent transmission and normalization factors, the peak intensity of the pure first-order component relative to the on-axis co-polarized reference scales as

$$\boxed{R^2 \propto \Lambda^2 G_{\mathrm{pk}}^2 r_{\max}^2 \propto \frac{G_{\mathrm{pk}}^2 \omega_f^4}{\omega_f^2 + \omega_m^2}.} \tag{63}$$

For $\omega_m = \omega_f$,

$$R^2 \propto G_{\mathrm{pk}}^2 \omega_f^2. \tag{64}$$

E. Experimental consequence: beam-size scaling at fixed fiber mode

The angular and real-space terms in Eqs. (55) and (56) have different dependences on the collimated beam radius. For a fixed local Jones gradient,

$$G_{\mathrm{pk}}^{(\Theta)} \propto \omega_0^0, \qquad G_{\mathrm{pk}}^{(r)} \propto \omega_0^2. \tag{65}$$

The ω_0^2 factor follows from the Fourier conversion of a fixed local gradient; it does not require the gradient itself to increase with the sampled area.

In the experiment, ω_m is fixed and

$$\omega_f = \frac{\lambda f}{\pi \omega_0} = \frac{C}{\omega_0}, \qquad C = \frac{\lambda f}{\pi}. \tag{66}$$

Substitution into Eq. (63) gives

$$R_\Theta^2 \propto \frac{1}{\omega_0^2\,(C^2+\omega_m^2\omega_0^2)}, \tag{67}$$

$$R_r^2 \propto \frac{\omega_0^2}{C^2+\omega_m^2\omega_0^2}. \tag{68}$$

The crossover occurs at

$$\omega_0^\star = \frac{C}{\omega_m} = \frac{\lambda f}{\pi\omega_m}, \qquad \omega_f = \omega_m. \tag{69}$$

The limiting behaviors are

Regime	Waist relation	angular R_Θ^2	real-space R_r^2
$\omega_0 \ll \omega_0^\star$	$\omega_f \gg \omega_m$	$\propto \omega_0^{-2}$	$\propto \omega_0^{+2}$
$\omega_0 \gg \omega_0^\star$	$\omega_f \ll \omega_m$	$\propto \omega_0^{-4}$	$\to$ const.

Within the first-order model, R_Θ^2 decreases monotonically with ω_0, whereas R_r^2 is non-decreasing and saturates above the crossover. The sign of the measured beam-size dependence therefore distinguishes these two channels without requiring a fitted power-law exponent.

F. Magnitude estimates

For an ideal laterally uniform QWP at exact normal incidence, the first-order angular coefficients vanish by symmetry. To illustrate the scale introduced by a small residual angle of incidence, we use the vendor retardance data of Supplementary Table 7. Near normal incidence, a quadratic fit gives

$$\Phi(\theta_{\rm AOI}) \simeq \Phi_0 - a\theta_{\rm AOI}^2, \qquad a \simeq 33\ {\rm rad}^{-1} \quad (|\theta_{\rm AOI}| \le 4.5^\circ), \tag{70}$$

with angles expressed in radians. For an illustrative residual tilt $\theta_{\rm AOI} = 1$ mrad,

$$\left|\frac{d\Phi}{d\Theta}\right| \simeq 2a\theta_{\rm AOI} \simeq 6.6\times10^{-2}. \tag{71}$$

Using

$$f = 26\ {\rm mm}, \qquad \lambda = 850\ {\rm nm}, \qquad \omega_0 = 2.8\ {\rm mm}, \qquad \omega_f \simeq 2.5\ \mu{\rm m}, \tag{72}$$

the corresponding retardance-dispersion contribution is

$$R_\Theta^2 \sim \left[\frac{\omega_f}{f}\left|\frac{d\Phi}{d\Theta}\right|\right]^2 \sim 4\times10^{-11}. \tag{73}$$

This value is conditional on the assumed 1 mrad residual tilt and scales as $\theta_{\rm AOI}^2$. It estimates the AOI-dependent retardance contribution and is not a general upper bound on all angular imperfections.

The real-space scale can be obtained directly from the fitted first-order coefficient. Within the real-space-dominated interpretation supported by the beam-size trend,

$$G_{p,s}^{(r)} = -i\frac{\ell}{f}\boldsymbol{a}^\dagger \bar{Q}_{3,4}\boldsymbol{e}_{\rm P}, \qquad \ell = \frac{k_0\omega_0^2}{2}. \tag{74}$$

The fit therefore determines the Jones-projected gradient

$$g_{\rm eff} \equiv \left(\left|\boldsymbol{a}^\dagger\bar{Q}_3\boldsymbol{e}_{\rm P}\right|^2 + \left|\boldsymbol{a}^\dagger\bar{Q}_4\boldsymbol{e}_{\rm P}\right|^2\right)^{1/2} = \frac{f}{\ell}\frac{|\boldsymbol{G}^{\rm fit}|}{\Lambda}. \tag{75}$$

For $\omega_0 = 2.795$ mm, Supplementary Table 3 gives $|\boldsymbol{G}^{\rm fit}| = 0.146\ {\rm mm}^{-1}$. With $\omega_f = 2.52\ \mu$m, $\Lambda = 0.503$, and $\ell/f = 1.11\times10^3$, this yields

$$g_{\rm eff} \simeq 0.26\ {\rm m}^{-1}, \qquad \delta_{\rm eff} \equiv g_{\rm eff}\omega_0 \simeq 7.3\times10^{-4}. \tag{76}$$

Here $\delta_{\rm eff}$ is the dimensionless variation of the analyzer-projected Jones response across one beam radius.

The magnitude estimate and the fitted-coefficient inversion establish the relevant scale. The discrimination between the angular and real-space channels follows from their opposite beam-size dependences.

Supplementary Note 4. EXPERIMENTAL CONTROLS AND SCALING ANALYSES

A. Direct imaging without single-mode-fiber detection

To verify that the two-lobe pattern is present in the optical field itself, rather than generated by the scanning single-mode fiber, we recorded the cross-polarized beam directly with a camera placed after the analyzer and before the collecting objective [Supplementary Fig. 4]. The two-lobe pattern is observed for multiple polarizer–QWP configurations. The scanning single-mode fiber samples this field through its overlap with a displaced Gaussian mode, but does not generate the two-lobe structure.

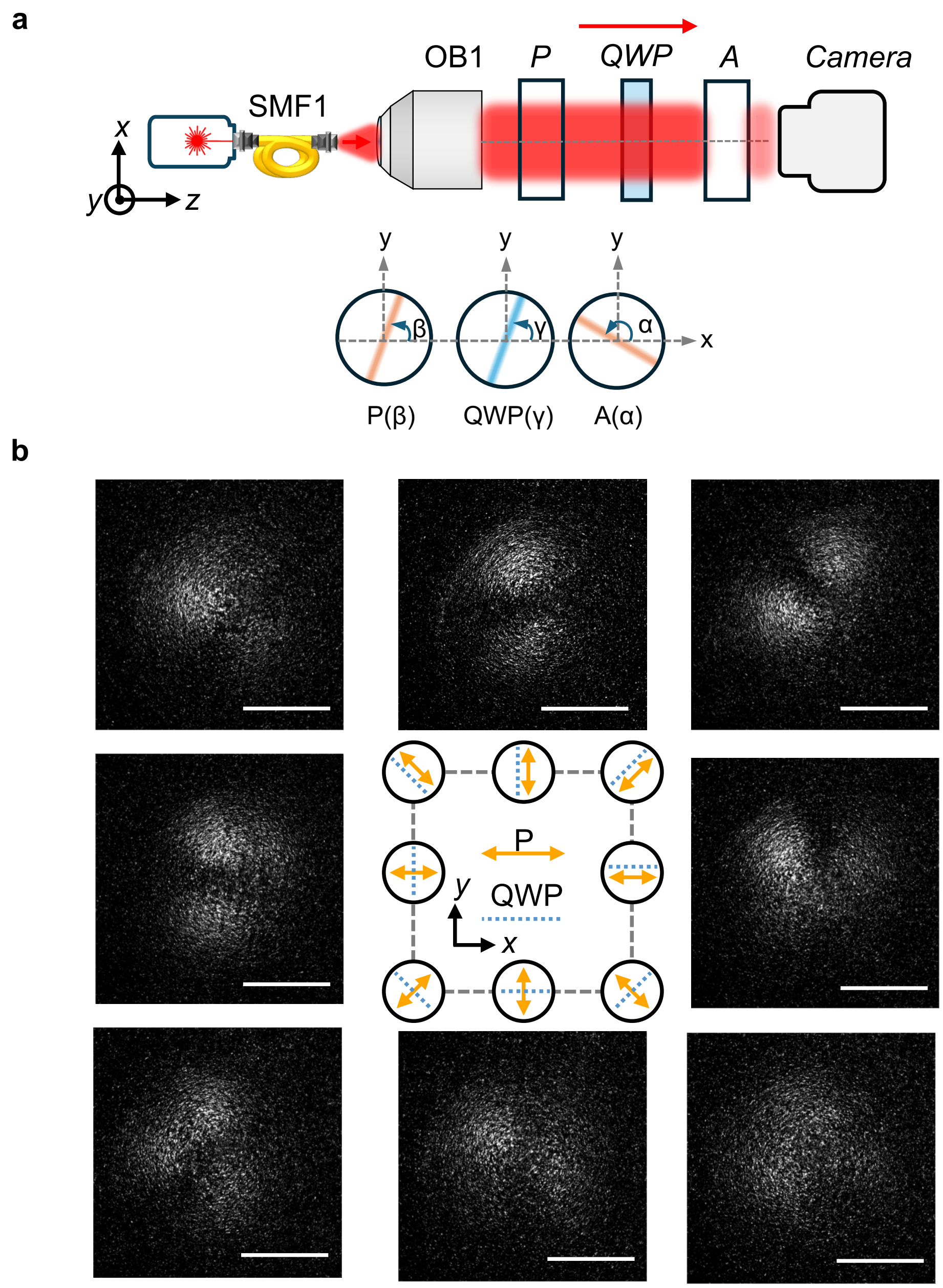

Supplementary Figure 4. **Direct camera imaging of the cross-polarized field before single-mode-fiber collection.** **a**, Schematic of the direct-imaging configuration, in which the cross-polarized field is recorded on a camera instead of being collected by the scanning single-mode fiber. **b**, Camera images measured for different polarizer and QWP orientations. The two-lobe structure is already visible in free space before fiber coupling, confirming that the modal transformation is generated by the optical system before detection and is not an artifact of the scanning-fiber measurement. Scale bar: 2.5 mm.

B. Comparison between different QWPs and wavelengths

We compared QWPs with different construction, material, retardance order, and operating wavelength [Supplementary Fig. 5]. Measurements at 850 nm used a diode laser module (LDT-850-10G, TOPAG Lasertechnik), while all measurements at 633 nm used a He–Ne laser (HNL100LB, Thorlabs). The extinction enhancement and two-lobe cross-polarized maps are reproduced across these elements, while the absolute extinction ratio and lobe intensity depend on the plate. This behavior is expected for a real-QWP first-order response, because the first-order angular and spatial coefficients $\bar{Q}_1$, $\bar{Q}_2$, $\bar{Q}_3$, and $\bar{Q}_4$ introduced in Supplementary Note 2 and Supplementary Note 3 are element dependent. The effect is also observed in polymer and MgF_2 plates, so it is not specific to quartz.

The plate dependence of the absolute extinction is pronounced. Whereas the 850 nm quartz zero-order QWP used in the main experiment reaches $\sim 10^7$, the 850 nm polymer zero-order QWP reaches an extinction ratio beyond 10^9 [Supplementary Fig. 5a]. At that level the measured cross-polarized signal reaches the overall experimental readout noise floor, whose individual contributions (photodetector, electronics, and multimeter) have not been separated; the quoted value is therefore a lower bound on the extinction achievable with this plate rather than a precisely resolved extinction ratio. This behavior is consistent with the plane-wave analysis of Supplementary Note 1, which allows cancellation of the mean leakage.

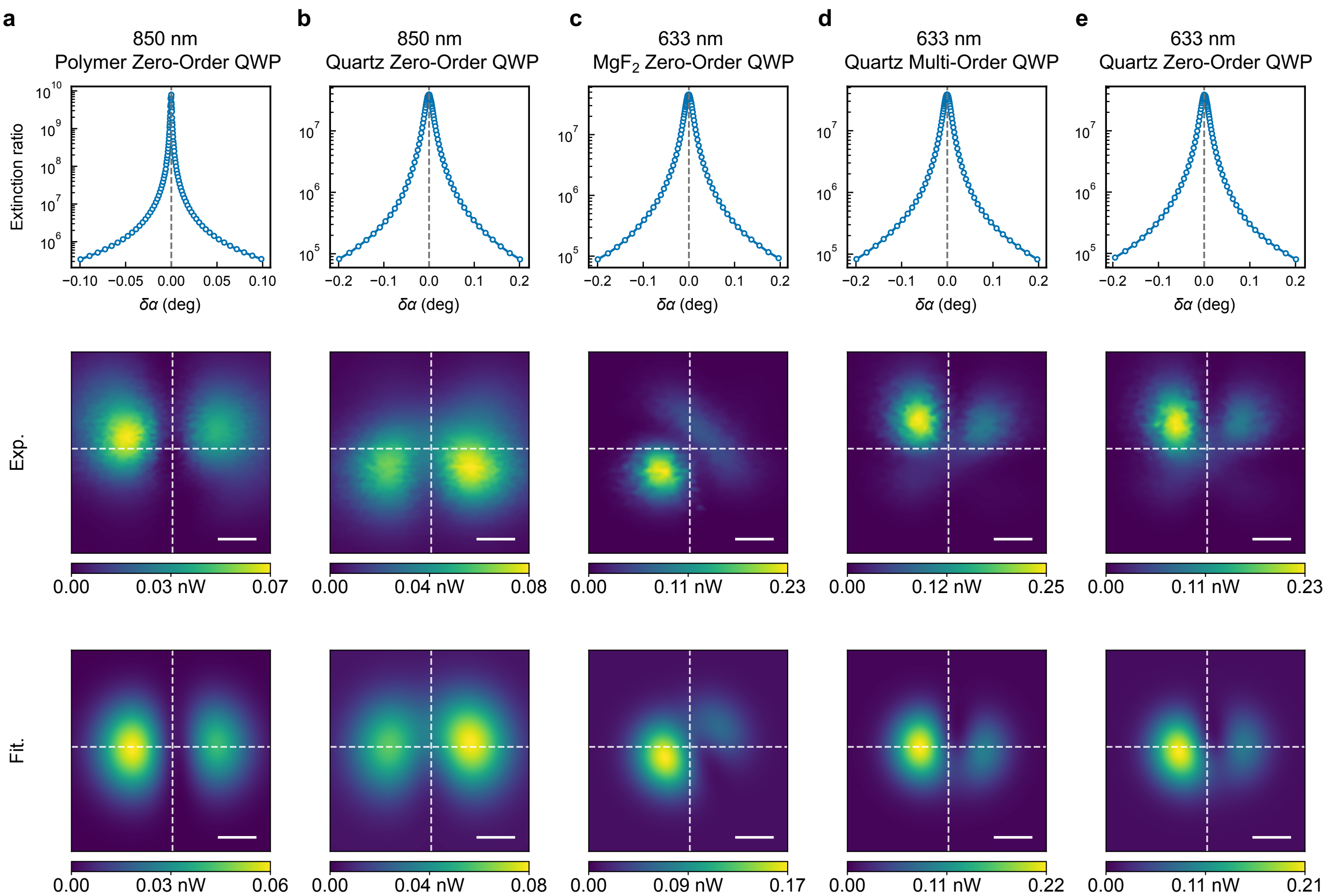

Supplementary Figure 5. **Comparison of extinction enhancement and cross-polarized modal profiles for different QWPs. a–e**, Measurements for an 850 nm polymer zero-order QWP (**a**), an 850 nm quartz zero-order QWP (**b**), a 633 nm MgF_2 zero-order QWP (**c**), a 633 nm quartz multi-order QWP (**d**) and a 633 nm quartz zero-order QWP (**e**). The top row shows the extinction ratio as a function of analyzer detuning $\delta\alpha$, with the dashed vertical line marking the optimized extinction condition. The middle row shows the measured cross-polarized confocal maps at the extinction maximum, and the bottom row shows the corresponding fits using the first-order modal model. The two-lobe cross-polarized mode is observed for different materials, retardance orders, and wavelengths, whereas the absolute extinction level, lobe asymmetry, and fitted modal coefficients are plate dependent.

C. Beam-size dependence

As derived in Sec. Supplementary Note 3 E, the angular and real-space first-order mechanisms predict opposite beam-size trends. The sign of the measured trend therefore distinguishes between these two channels.

The beam size is varied by two independent methods. In the primary (iris) method [Supplementary Fig. 6], an iris diaphragm placed between the polarizer and the QWP sets the beam footprint while the optical path, alignment, and focusing geometry remain unchanged; for each aperture setting the effective Gaussian radius ω_0 is inferred from the co-polarized confocal data. Supplementary Figure 7 shows the resulting scaling for four QWPs of different material, retardance order, and design wavelength: in all cases the normalized lobe intensity increases with ω_0 and follows the spatial prediction.

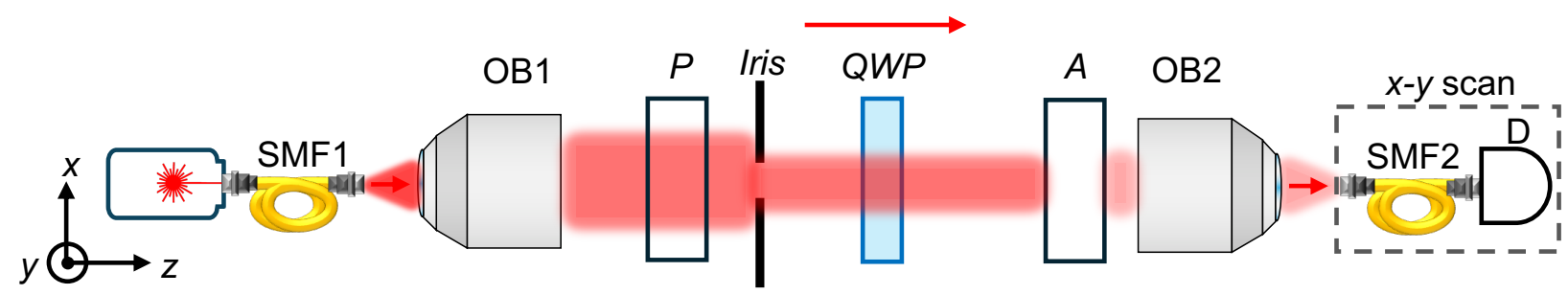

Supplementary Figure 6. **Experimental configuration for beam-size-dependent measurements (iris method).** The beam radius ω_0 at the QWP is varied with an iris diaphragm placed between the polarizer P and the QWP, while the optical path, alignment, and crossed-polarization confocal detection geometry remain unchanged. For each aperture setting, the effective Gaussian radius ω_0 is inferred from the co-polarized confocal data.

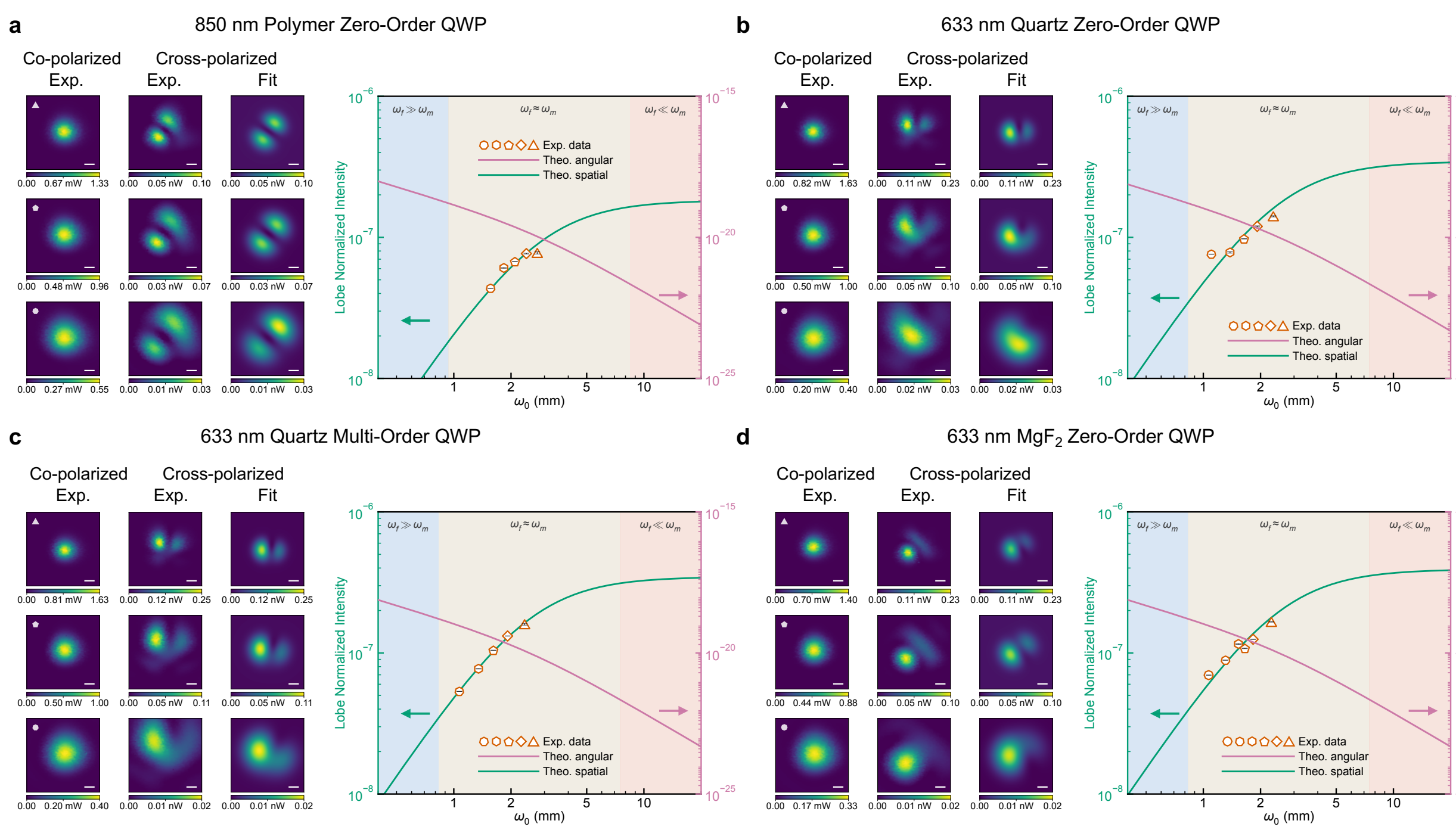

Supplementary Figure 7. **Beam-size scaling for different QWPs (iris method).** **a**–**d**, Confocal maps (co-polarized experiment, cross-polarized experiment, cross-polarized fit; beam radius ω_0 increasing bottom to top) and the corresponding beam-size scaling for an 850 nm polymer zero-order QWP (**a**), a 633 nm quartz zero-order QWP (**b**), a 633 nm quartz multi-order QWP (**c**), and a 633 nm MgF_2 zero-order QWP (**d**). The symbol in the corner of each co-polarized map marks the corresponding point in the scaling plot. Scaling plots show the normalized lobe intensity (peak cross-polarized lobe intensity over peak co-polarized intensity) versus ω_0 (log–log); symbols are measured data (shape encodes ω_0, triangle = largest). Solid curves are the two first-order models on separate axes: the real-space (spatial) prediction (green, left axis) and the angular-spectrum prediction (pink, right axis), with arrows indicating each axis. Shaded bands mark the $\omega_f \gg \omega_m$, $\omega_f \approx \omega_m$, and $\omega_f \ll \omega_m$ regimes (ω_f: focal-spot radius, ω_m: fiber mode radius). Error bars: spread between forward and backward fiber scans. For all QWPs the data track the spatial curve, supporting a dominant real-space QWP nonuniformity contribution. Scale bars: 2.5 μm.

As an independent cross-check, the telescope (lens) method [Supplementary Fig. 8] sets ω_0 by changing the telescope

formed by lenses f_1 and f_2 before the polarizer, avoiding any truncation of the beam at the cost of re-collimation for each beam size. For the two plates measured with both methods—the 850 nm polymer zero-order QWP and the 850 nm quartz zero-order QWP of Fig. 3 of the main text—the trends agree [Supplementary Fig. 9]. The two methods have complementary systematics (aperture truncation and edge diffraction for the iris; re-collimation and alignment for the telescope), so their agreement makes a method-specific artifact an unlikely origin of the positive scaling. Error bars in all scaling plots give the spread between values extracted from forward and backward fiber scans (Methods of the main text).

The positive scaling, reproduced across QWP materials, retardance orders, wavelengths, and beam-size-control methods, provides the principal experimental evidence that the measured lobe intensity is dominated by the real-space Jones-gradient channel rather than the angular channel.

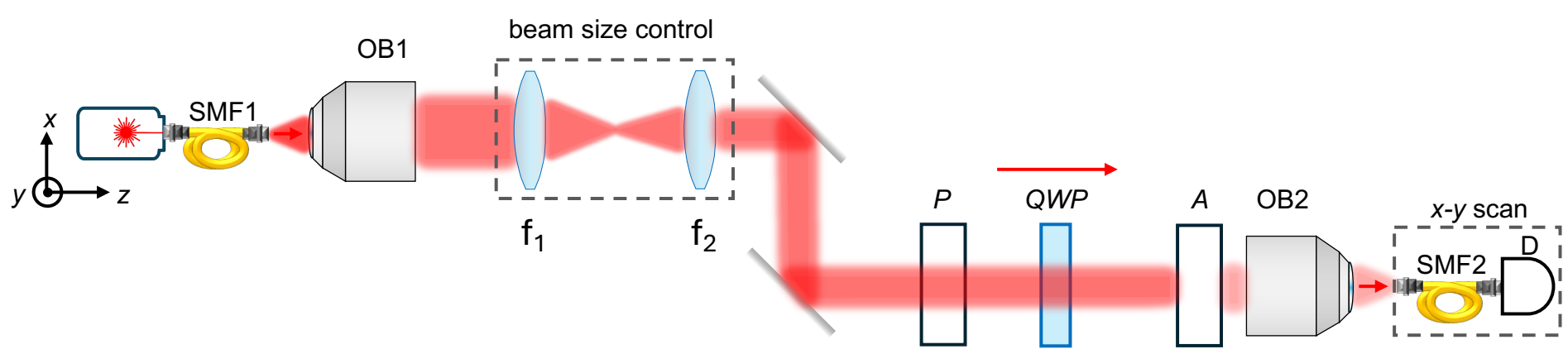

Supplementary Figure 8. **Experimental configuration for the telescope cross-check (lens method).** The collimated beam radius ω_0 at the QWP is set by changing the telescope formed by lenses f_1 and f_2 before the polarizer, while the crossed-polarization confocal detection geometry is kept unchanged. In contrast to the iris method, the beam is not truncated, but the collimation is re-established for each beam size.

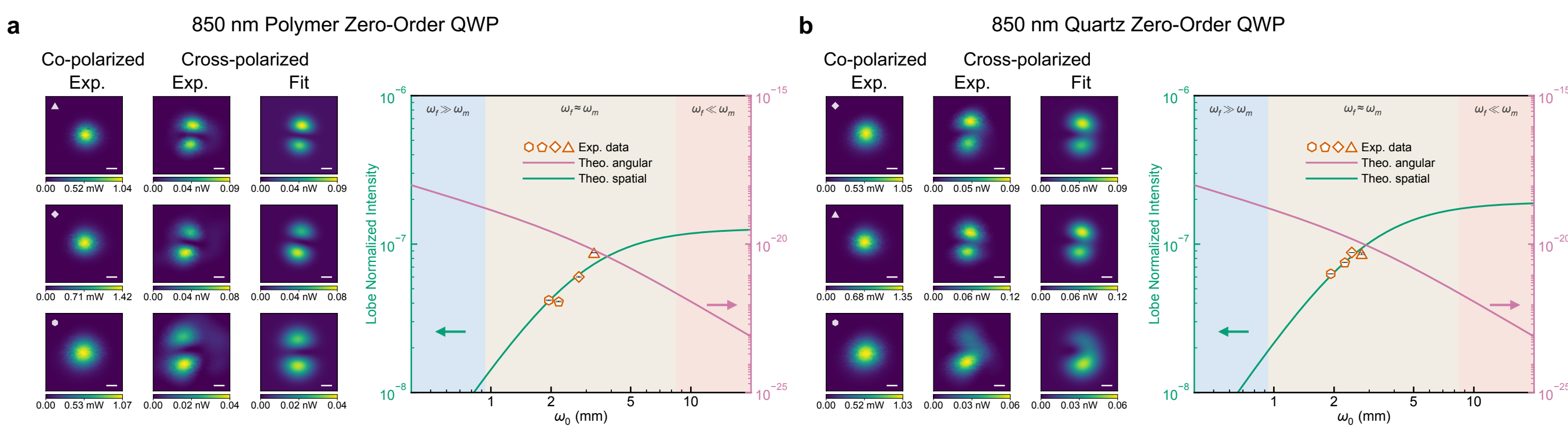

Supplementary Figure 9. **Beam-size scaling obtained with the telescope method.** **a**,**b**, Confocal maps (co-polarized experiment, cross-polarized experiment, cross-polarized fit) and the corresponding beam-size scaling for the 850 nm polymer zero-order QWP (**a**) and the 850 nm quartz zero-order QWP (**b**)—the same plates measured with the iris method in Supplementary Fig. 7a and in Fig. 3 of the main text, respectively. The symbol in the corner of each co-polarized map marks the corresponding point in the scaling plot; scaling plots, axes, and shaded bands as in Supplementary Fig. 7. Error bars: spread between forward and backward fiber scans. For both plates the normalized lobe intensity increases with ω_0 and tracks the spatial prediction, in agreement with the iris method. Scale bars: 2.5 μm.

D. Tilt-angle dependence

Tilting the QWP intentionally increases the angular symmetry breaking and therefore changes the two-lobe orientation and the lobe intensity [Supplementary Fig. 10]. The two-lobe mode is nevertheless already present at nominal normal incidence, and the beam-size measurements above show that the intensity scale of the main experiment is not set by the angular channel.

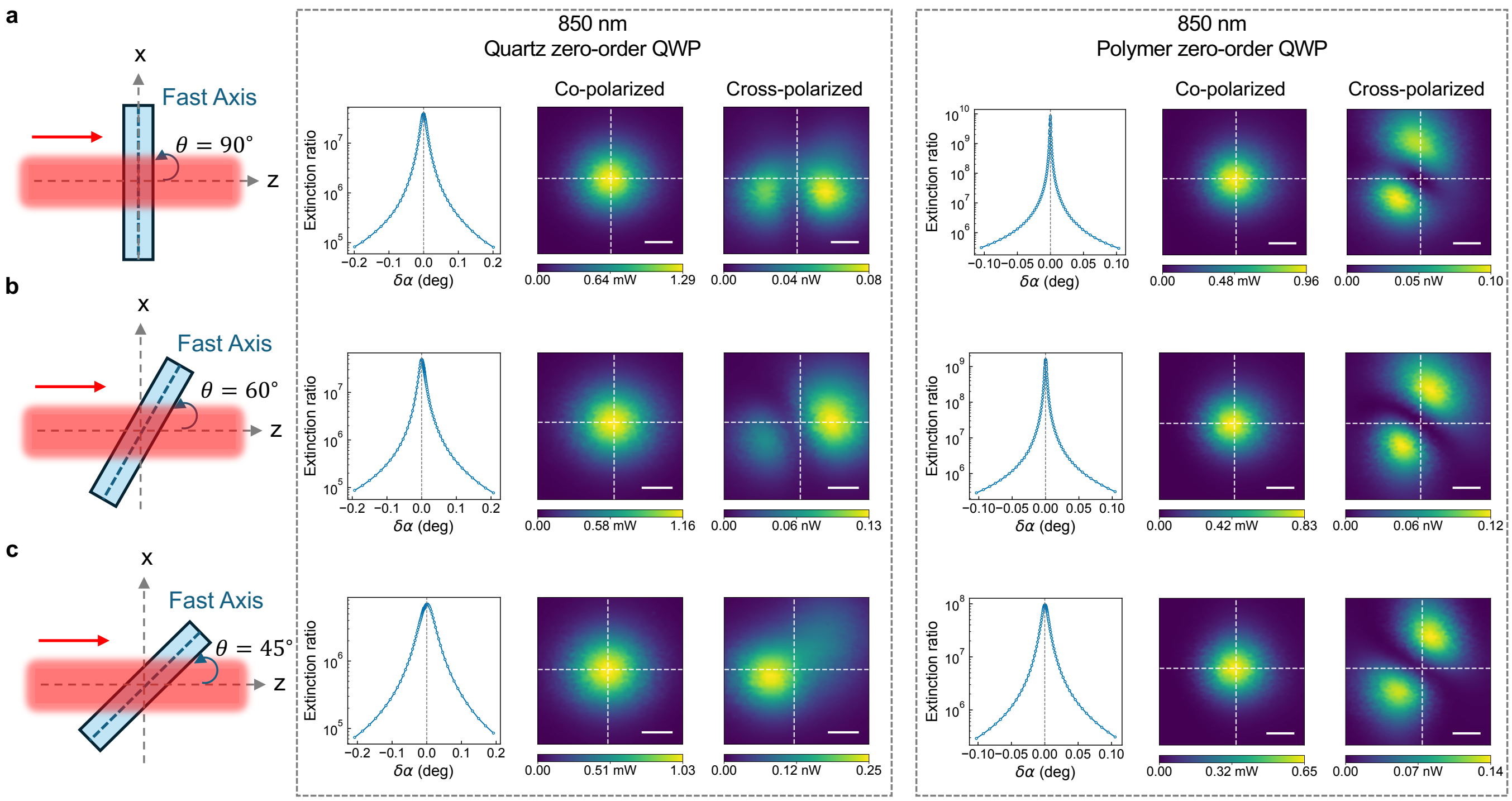

Supplementary Figure 10. **Tilt-angle dependence of the cross-polarized mode for 850 nm zero-order QWPs. a–c**, Measurements for three QWP orientations, defined by the angle θ between the optic (fast) axis of the plate and the optical axis in the tilt plane, i.e. the same θ as in Supplementary Note 2: $\theta = 90^\circ$ (**a**, nominal normal incidence), 60° (**b**), and 45° (**c**). For each angle, the left schematic defines the QWP orientation. The subsequent panels show, for quartz and polymer zero-order QWPs, the extinction ratio as a function of analyzer detuning $\delta\alpha$, followed by the co-polarized and cross-polarized confocal maps at the optimized extinction condition. Tilting the QWP modifies both the extinction curve and the cross-polarized two-lobe pattern. The two-lobe mode is already present at nominal normal incidence, showing that deliberate tilt is not required for the effect observed in the main experiment.

E. Anti-crossing behavior of the mode peak

Here we extract the peak positions as a function of the analyzer offset $\delta\alpha \equiv \alpha - \alpha_{\max}$, where $\alpha_{\max}$ is the analyzer angle at which the maximum extinction ratio is obtained, from the data shown in Fig. 2 of the main text. For each analyzer setting, the positions of the dominant peaks are identified and plotted versus $\delta\alpha$. The two branches approach each other near $\delta\alpha = 0°$ but remain separated, showing an anti-crossing behavior [Supplementary Fig. 11] similar to that reported in Ref. [1].

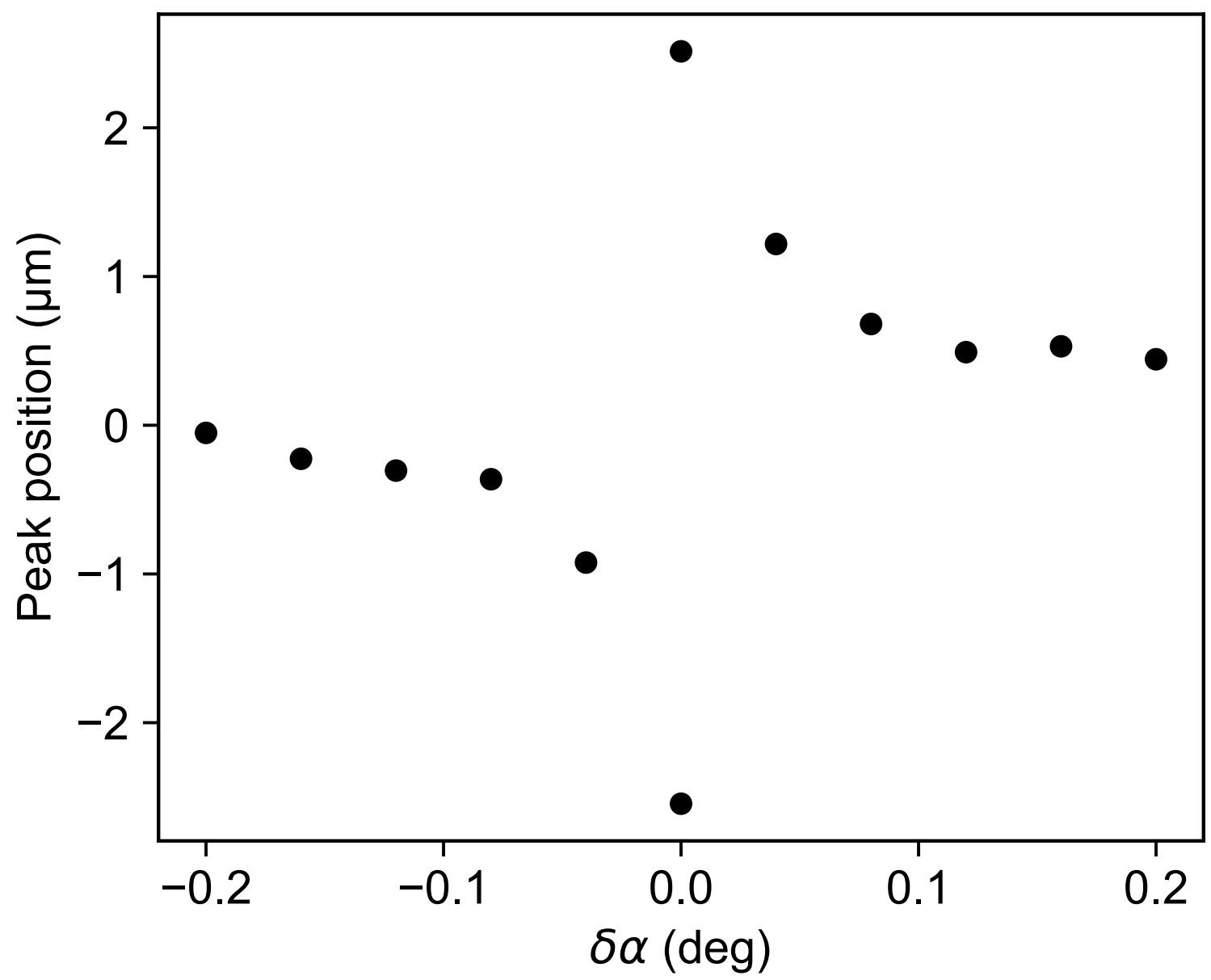

Supplementary Figure 11. **Analyzer-dependent evolution of the extracted lobe peak positions.** Peak positions extracted from the cross-polarized maps in Fig. 2 of the main text are plotted as functions of the analyzer detuning $\delta\alpha$. The two dominant branches approach each other near $\delta\alpha = 0°$ but do not cross, producing an avoided-crossing-like trajectory. This behavior reflects the continuous interference between the residual Gaussian leakage and the first-order derivative mode as the analyzer is tuned through the extinction point.

F. Extraction of lobe orientation

For each cross-polarized map in Fig. 4 of the main text, the fitted beam center is used as the coordinate origin, and the lobe orientation is obtained from the principal axis connecting the two bright lobes [Supplementary Fig. 12]. The lobe orientation varies approximately linearly with the QWP fast-axis angle for both parallel and perpendicular input-polarization configurations.

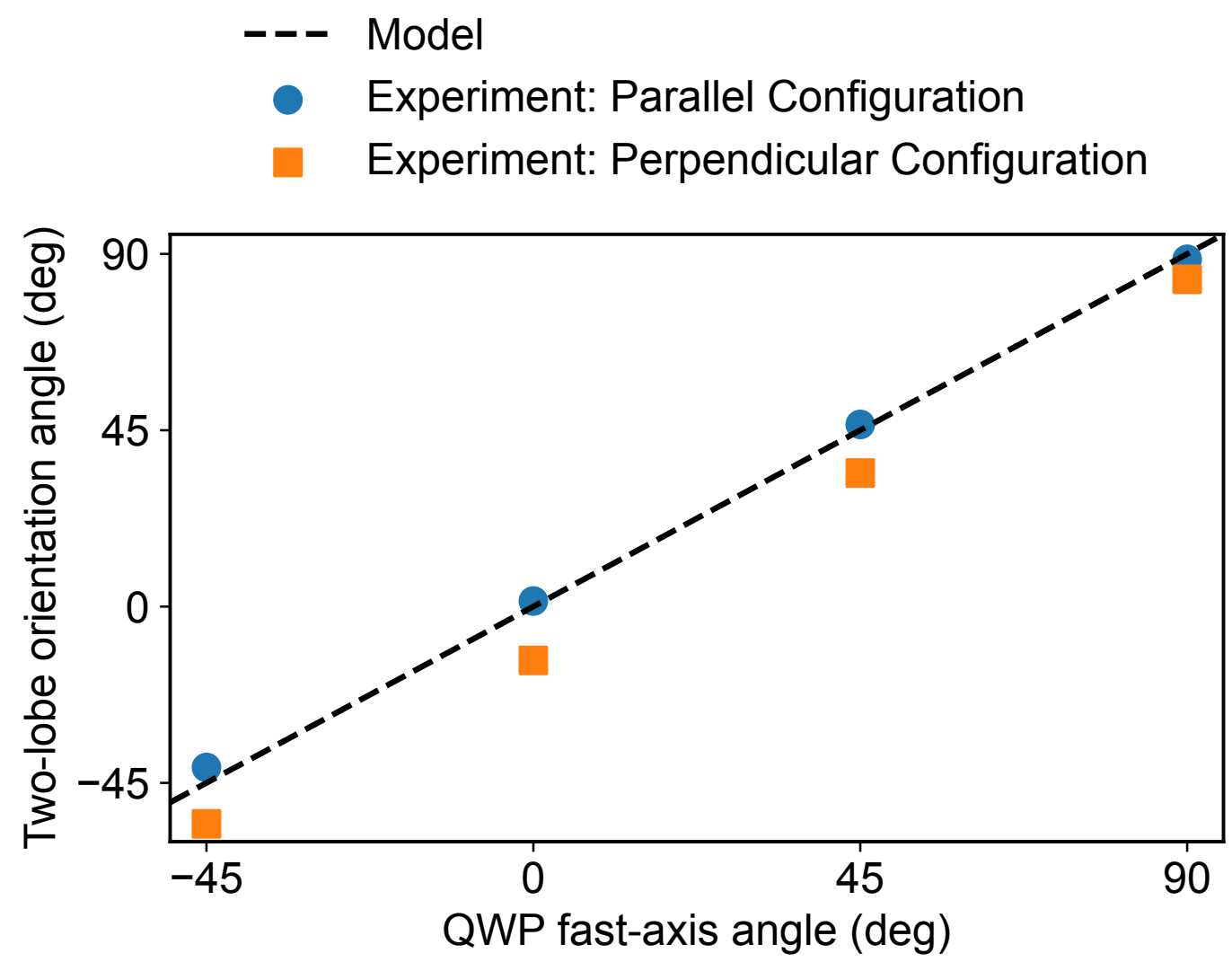

Supplementary Figure 12. **Extraction of the two-lobe orientation.** The measured orientation angle of the cross-polarized two-lobe mode is plotted as a function of the QWP fast-axis angle, for the configurations of Fig. 4 of the main text (there the QWP fast axis is represented by a blue dotted line in each configuration icon). Data are shown for input polarization parallel and perpendicular to the QWP fast axis. The dashed line indicates the orientation expected from the first-order modal model. The agreement supports the interpretation that the lobe orientation is controlled by the QWP fast-axis rotation.

Supplementary Note 5. FULL VECTORIAL NON-PARAXIAL CALCULATION FOR AN IDEAL QWP

A. Motivation

We use the Richards–Wolf formalism [6–9] to determine whether finite-NA collimation can produce the cross-polarized two-lobe mode observed near extinction. The QWP is ideal and laterally uniform. The polarizer and analyzer follow the spatially uniform rank-one leakage model of Supplementary Note 1, with $b = 0.0036$.

The calculation proceeds in two steps. We first propagate the fiber mode through the collimating objective and obtain E_x, E_y, and E_z immediately before the polarization optics [Supplementary Fig. 14a]. We then apply the polarizer–QWP–analyzer sequence [Supplementary Fig. 14b]. The polarizer angle is chosen so that the optimized plane-wave transmission reproduces the measured extinction ratio, $\mathrm{ER} = 4.2 \times 10^7$. This calibration fixes the cross-polarized intensity scale but not its spatial profile.

Finite-NA collimation yields an even four-lobe transverse background. After the spatially uniform polarization chain, the cross-polarized transverse profile remains a node-free Gaussian, while the only first-order two-lobe component is longitudinal and negligible. The model therefore does not reproduce the observed transverse first-order mode.

B. Vectorial mapping by the collimating objective

Supplementary Figure 13 shows the geometry. A single-mode fiber emits into a microscope objective with NA = 0.25. Each ray is labelled by the polar angle θ and azimuth ϕ, with local basis vectors $\boldsymbol{n}_\phi$ and $\boldsymbol{n}_\theta$:

$$\boldsymbol{n}_\phi = -\sin\phi\, \boldsymbol{e}_x + \cos\phi\, \boldsymbol{e}_y, \tag{77}$$

$$\boldsymbol{n}_\theta = \cos\theta\cos\phi\, \boldsymbol{e}_x + \cos\theta\sin\phi\, \boldsymbol{e}_y - \sin\theta\, \boldsymbol{e}_z. \tag{78}$$

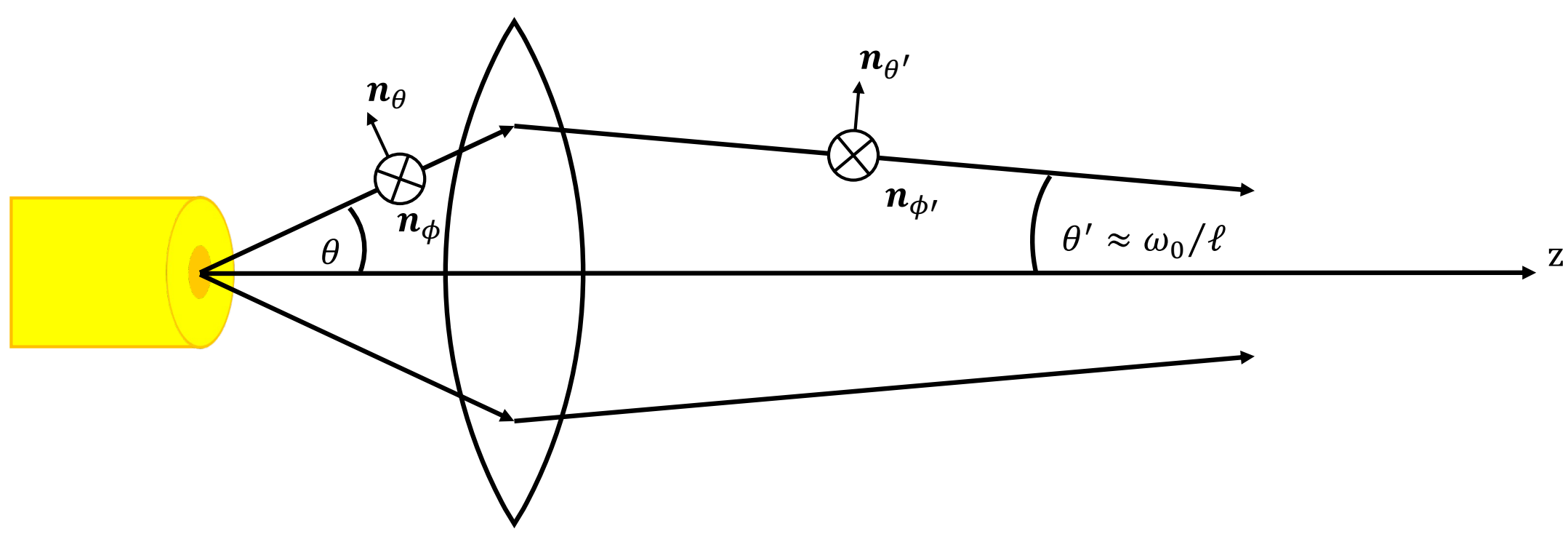

Supplementary Figure 13. **Geometry of the vectorial collimation problem.** A ray emitted at (θ, ϕ) is mapped by the objective to the outgoing angle θ' at the same azimuth. The local basis $(\boldsymbol{n}_\theta, \boldsymbol{n}_\phi)$ is correspondingly rotated to $(\boldsymbol{n}_{\theta'}, \boldsymbol{n}_{\phi'})$ and then expressed in the laboratory Cartesian basis.

After collimation, each plane-wave component retains a small propagation angle θ'. We keep this residual angular spread rather than set $\theta' = 0$:

$$\theta' \approx \frac{\omega_0}{\ell}, \tag{79}$$

where $\omega_0 \approx 2.8$ mm is the beam radius at the QWP and $\ell = k_0\omega_0^2/2 \approx 29$ m is the corresponding Rayleigh length. The characteristic outgoing divergence is $\theta_0 = \omega_0/\ell = 2/(k_0\omega_0)$, using the notation of Supplementary Note 2. For an aplanatic objective, $\omega_0 = f \sin\theta_\mathrm{m} \simeq f\theta_\mathrm{m}$, with $\theta_\mathrm{m} = \lambda/(\pi\omega_\mathrm{m})$, giving $\omega_0 = 2.81$ mm, consistent with experiment. The collimated-beam Rayleigh length ℓ is distinct from the fiber-side Rayleigh range z_R used below.

The two angular scales are widely separated,

$$\theta \lesssim \theta_\mathrm{m} = \frac{\lambda}{\pi\omega_\mathrm{m}} \approx 1.1 \times 10^{-1}\ \mathrm{rad}, \qquad \theta' \lesssim \frac{\omega_0}{\ell} = \theta_0 \approx 1.0 \times 10^{-4}\ \mathrm{rad}, \qquad \frac{\theta}{\theta'} \approx \frac{\ell}{f} \approx 1.1 \times 10^3, \tag{80}$$

so their characteristic angular scales differ by approximately three orders of magnitude.

The objective leaves $\boldsymbol{n}_\phi$ unchanged and maps $\boldsymbol{n}_\theta(\theta)$ to $\boldsymbol{n}_\theta(\theta')$. In the meridional plane this is a rotation by $-\Delta\theta$, where

$$\Delta\theta \equiv \theta - \theta' \ (> 0 \text{ for a collimator}). \tag{81}$$

Resolving the field in the local basis, applying this rotation, and transforming back gives $\boldsymbol{M}(\theta,\theta',\phi) = \boldsymbol{R}\boldsymbol{T}\boldsymbol{R}^{-1}$:

$$\boldsymbol{M} = \begin{pmatrix} \cos\phi & -\sin\phi & 0 \\ \sin\phi & \cos\phi & 0 \\ 0 & 0 & 1 \end{pmatrix} \begin{pmatrix} \cos\Delta\theta & 0 & -\sin\Delta\theta \\ 0 & 1 & 0 \\ \sin\Delta\theta & 0 & \cos\Delta\theta \end{pmatrix} \begin{pmatrix} \cos\phi & \sin\phi & 0 \\ -\sin\phi & \cos\phi & 0 \\ 0 & 0 & 1 \end{pmatrix}. \tag{82}$$

Aplanatic power conservation gives the apodization factor

$$A(\theta,\theta') = \sqrt{\frac{\cos\theta'}{\cos\theta}}. \tag{83}$$

The field immediately after the objective is therefore

$$\boldsymbol{E}_{\text{coll}}(\theta,\phi) = \sqrt{\frac{\cos\theta'}{\cos\theta}}\ \boldsymbol{M}(\theta,\theta',\phi)\ \boldsymbol{E}_{\text{inc}}(\theta,\phi). \tag{84}$$

C. Incident field: angular spectrum of the fiber mode

We begin with the scalar Gaussian field of the fiber mode,

$$E(\rho,z) \approx E_0 \frac{z_R}{z} \exp\left(-\frac{\rho^2}{w(z)^2}\right) \exp\left(-ikz - i\frac{k\rho^2}{2R(z)} + i\psi(z)\right), \tag{85}$$

where ω_{m} is the mode-field radius, $z_R = k_0\omega_{\text{m}}^2/2$, $w(z) = \omega_{\text{m}}\sqrt{1+(z/z_R)^2}$, $R(z) = z[1+(z_R/z)^2]$, and $\psi(z) = \arctan(z/z_R)$. In the far field, $z \gg z_R$, we use $z \simeq r$, $\rho \simeq r\theta$, $R \simeq r$, $\psi \to \pi/2$, and $w(z) \simeq \theta_{\text{m}} r$, with $\theta_{\text{m}} = \lambda/(\pi\omega_{\text{m}})$. The scalar amplitude on a spherical reference surface is then

$$\Psi(\theta) \ = \ E_0 \frac{z_R}{r} \ \exp\left(-\frac{\theta^2}{\theta_{\text{m}}^2}\right) e^{-ikr + i\pi/2}. \tag{86}$$

The fiber mode is polarized along $\hat{\boldsymbol{e}}_x$ at the facet, but each radiated plane-wave component must remain transverse to its local propagation direction,

$$\hat{\boldsymbol{k}}(\theta,\phi) = (\sin\theta\cos\phi,\ \sin\theta\sin\phi,\ \cos\theta), \qquad \hat{\boldsymbol{k}}\cdot\boldsymbol{E}_{\text{inc}} = 0. \tag{87}$$

Because $\hat{\boldsymbol{e}}_x\cdot\hat{\boldsymbol{k}} = \sin\theta\cos\phi$, transversality fixes the longitudinal component of an angular-spectrum field with transverse part $\hat{\boldsymbol{e}}_x\Psi$:

$$\boxed{\boldsymbol{E}_{\text{inc}}(\theta,\phi) = \Psi(\theta)\,[\hat{\boldsymbol{e}}_x - \tan\theta\cos\phi\,\hat{\boldsymbol{e}}_z]} \tag{88}$$

which satisfies $\hat{\boldsymbol{k}}\cdot\boldsymbol{E}_{\text{inc}} = 0$ identically. Its longitudinal amplitude is of order $1/(k\omega_{\text{m}}) \approx 5\times10^{-2}$ at the fiber output.

D. Analytical evaluation of the transformed pupil field

We now evaluate $\boldsymbol{M}\boldsymbol{E}_{\text{inc}}$, using φ for the pupil azimuth and ϕ_r for the observation azimuth. In the local ray frame,

$$\boldsymbol{R}^{-1}\,[\hat{\boldsymbol{e}}_x - \tan\theta\cos\varphi\,\hat{\boldsymbol{e}}_z] = \begin{pmatrix} \cos\varphi \\ -\sin\varphi \\ -\tan\theta\cos\varphi \end{pmatrix}, \tag{89}$$

which is transverse to the incident ray direction $(\sin\theta, 0, \cos\theta)$. Applying $\boldsymbol{T} = \boldsymbol{R}_y(-\Delta\theta)$ gives

$$\boldsymbol{T}\,\boldsymbol{R}^{-1}\boldsymbol{E}_{\text{inc}}/\Psi = \begin{pmatrix} \cos\varphi\,[\cos\Delta\theta + \sin\Delta\theta\tan\theta] \\ -\sin\varphi \\ \cos\varphi\,[\sin\Delta\theta - \cos\Delta\theta\tan\theta] \end{pmatrix} = \begin{pmatrix} \cos\varphi\,\dfrac{\cos\theta'}{\cos\theta} \\ -\sin\varphi \\ -\cos\varphi\,\dfrac{\sin\theta'}{\cos\theta} \end{pmatrix}, \tag{90}$$

where $\cos\Delta\theta\cos\theta+\sin\Delta\theta\sin\theta=\cos\theta'$ and $\sin\Delta\theta\cos\theta-\cos\Delta\theta\sin\theta=-\sin\theta'$. Equation (90) is transverse to the outgoing direction $(\sin\theta',0,\cos\theta')$.

Rotating back to the laboratory frame with $\boldsymbol{R}$ and applying the double-angle identities gives the compact harmonic form

$$\boldsymbol{M}\boldsymbol{E}_{\mathrm{inc}}=\Psi(\theta)\begin{pmatrix}\frac{1}{2}\left(1+\dfrac{\cos\theta'}{\cos\theta}\right)+\frac{1}{2}\left(\dfrac{\cos\theta'}{\cos\theta}-1\right)\cos 2\varphi\\ \frac{1}{2}\left(\dfrac{\cos\theta'}{\cos\theta}-1\right)\sin 2\varphi\\ -\dfrac{\sin\theta'}{\cos\theta}\cos\varphi\end{pmatrix}. \tag{91}$$

Equation (91) contains three azimuthal orders: $n=0$ in the dominant co-polarized E_x field, $n=2$ in E_x and the cross-polarized E_y field, and $n=1$ in the longitudinal E_z field. Their scales differ. The transverse $n=2$ coefficient is

$$\frac{\cos\theta'}{\cos\theta}-1\simeq\frac{\theta^2-\theta'^2}{2}\simeq\frac{\theta^2}{2}, \tag{92}$$

whereas the longitudinal $n=1$ coefficient is $\sin\theta'/\cos\theta\simeq\theta'$. For the present beam, the corresponding pupil amplitudes are of order $\theta_{\mathrm{m}}^2/2\approx 6\times10^{-3}$ and $\theta'\approx10^{-4}$, respectively. After diffraction, these give a transverse cross-polarized amplitude of order 10^{-3} and a much weaker longitudinal field [Eq. (102)].

The longitudinal scale also follows from paraxial transversality. For an x-polarized Gaussian of radius ω_0, $E_z\simeq(i/k)(\partial_x E_x+\partial_y E_y)$ gives

$$\frac{\max|E_z|}{\max|E_x|}\simeq\frac{0.86}{k\omega_0}=4.1\times10^{-5}\qquad\Longrightarrow\qquad\frac{\max|E_z|^2}{\max|E_x|^2}\simeq1.7\times10^{-9}, \tag{93}$$

for $\lambda=850$ nm and $\omega_0=2.81$ mm. The Richards–Wolf result agrees with this estimate. A separate spectral reconstruction of E_z matches the direct calculation and satisfies $\boldsymbol{k}\cdot\boldsymbol{E}=0$.

E. Field on the outgoing reference sphere

The incident and outgoing angular coordinates are related by the aplanatic sine condition, $r=f\sin\theta$, and the far-field relation for the collimated beam, $\sin\theta'\simeq r/\ell$. Eliminating r gives

$$\sin\theta=\frac{\ell}{f}\sin\theta',\qquad\text{i.e.}\qquad\theta(\theta')=\arcsin\left(\frac{\ell}{f}\sin\theta'\right), \tag{94}$$

with $\ell/f\approx1.1\times10^3$. This maps $\exp(-\theta^2/\theta_{\mathrm{m}}^2)$ onto a collimated Gaussian angular spectrum of $1/e$ half-width $\theta_{\mathrm{m}}f/\ell=\omega_0/\ell$.

The electric field on the outgoing reference sphere is

$$\boldsymbol{E}_\Sigma(\theta',\varphi)=E_0'\sqrt{\frac{\cos\theta'}{\cos\theta(\theta')}}\exp\left[-\frac{\theta(\theta')^2}{\theta_{\mathrm{m}}^2}\right]\boldsymbol{\mathcal{H}}\big(\theta(\theta'),\theta',\varphi\big), \tag{95}$$

where $\boldsymbol{\mathcal{H}}$ is the harmonic vector in Eq. (91); radial prefactors and global phases are absorbed into E_0'.

F. Richards–Wolf integral and radial reduction

The field at $\boldsymbol{r}=(\rho\cos\phi_r,\rho\sin\phi_r,z)$ is

$$\boldsymbol{E}(\boldsymbol{r})=-\frac{ik}{2\pi}\int_0^{\theta'_{\max}}\int_0^{2\pi}\boldsymbol{E}_\Sigma(\theta',\varphi')\,e^{i\boldsymbol{k}'\cdot\boldsymbol{r}}\sin\theta'\,d\varphi'\,d\theta',\qquad\boldsymbol{k}'\cdot\boldsymbol{r}=k\rho\sin\theta'\cos(\varphi'-\phi_r)+kz\cos\theta'. \tag{96}$$

With $x'=k\rho\sin\theta'$, the Jacobi–Anger expansion and the identities

$$\int_0^{2\pi}\cos(n\varphi')e^{ix'\cos(\varphi'-\phi_r)}d\varphi'=2\pi i^n J_n(x')\cos(n\phi_r),\qquad\int_0^{2\pi}\sin(n\varphi')e^{ix'\cos(\varphi'-\phi_r)}d\varphi'=2\pi i^n J_n(x')\sin(n\phi_r), \tag{97}$$

reduce the diffraction integral to one-dimensional radial integrals. Since Eq. (91) contains only orders $n = 0, 1, 2$, only J_0, J_1, and J_2 appear.

Introducing the pupil weighting function

$$W(\theta') = \sqrt{\frac{\cos\theta'}{\cos\theta(\theta')}}\,\exp\left[-\frac{\theta(\theta')^2}{\theta_{\mathrm{m}}^2}\right] e^{ikz\cos\theta'}, \qquad c(\theta') \equiv \frac{\cos\theta'}{\cos\theta(\theta')}, \tag{98}$$

the three Cartesian components of the collimated field are

$$E_x(\rho,\phi_r) = -\frac{ik}{2}E_0'\int_0^{\theta'_{\max}} W(\theta')\sin\theta'\Big[\big(1+c\big)J_0(k\rho\sin\theta') - \big(c-1\big)J_2(k\rho\sin\theta')\cos 2\phi_r\Big]d\theta', \tag{99}$$

$$E_y(\rho,\phi_r) = \frac{ik}{2}E_0'\int_0^{\theta'_{\max}} W(\theta')\sin\theta'\,\big(c-1\big)J_2(k\rho\sin\theta')\sin 2\phi_r\,d\theta', \tag{100}$$

$$E_z(\rho,\phi_r) = -\,k\,E_0'\int_0^{\theta'_{\max}} W(\theta')\sin\theta'\,\frac{\sin\theta'}{\cos\theta(\theta')}\,J_1(k\rho\sin\theta')\cos\phi_r\,d\theta'. \tag{101}$$

Equations (99)–(101) give the vectorial field in terms of the outgoing angular spectrum. The incident angle enters only through $\theta = \theta(\theta')$.

For $\lambda = 850$ nm, $\omega_{\mathrm{m}} = 2.5$ μm, NA $= 0.25$, and $f = 26$ mm, the field immediately after collimation contains [Supplementary Fig. 14a]

$$\frac{\max|E_y|^2}{\max|E_x|^2} = 1.18\times10^{-6}\quad(\text{four-lobe},\ \sin 2\phi_r), \qquad \frac{\max|E_z|^2}{\max|E_x|^2} = 1.73\times10^{-9}\quad(\text{two-lobe},\ \cos\phi_r). \tag{102}$$

The longitudinal value agrees with Eq. (93).

G. Spatially uniform polarizer–QWP–analyzer model

We next apply an ideal, laterally uniform QWP and the rank-one polarizer/analyzer model used in the plane-wave calculation. For $j = P, A$,

$$\bar{L}_{0,j} = \begin{pmatrix} a_j & 0 \\ ib_j & 0 \end{pmatrix}, \qquad a_j^2 + b_j^2 = 1, \qquad b_P = b_A = 0.0036. \tag{103}$$

The rotated polarizer and analyzer and the ideal QWP are

$$\bar{P}(\beta) = \bar{R}(\beta)\bar{L}_{0,P}\bar{R}(-\beta), \qquad \bar{A}(\alpha) = \bar{R}(\alpha)\bar{L}_{0,A}\bar{R}(-\alpha), \qquad \bar{Q}(\gamma) = \bar{R}(\gamma)\bar{Q}_0\bar{R}(-\gamma), \tag{104}$$

where

$$\bar{Q}_0 = e^{-i\pi/4}\begin{pmatrix} 1 & 0 \\ 0 & i \end{pmatrix} = \mathrm{diag}(e^{-i\pi/4}, e^{+i\pi/4}). \tag{105}$$

Because these matrices are independent of transverse position and wave vector, they can mix existing transverse components but cannot create a new azimuthal order.

For $\tilde{\boldsymbol{E}}_\perp(\boldsymbol{k}_\perp) = (\tilde{E}_x, \tilde{E}_y)^T$, the transmitted spectrum is

$$\tilde{\boldsymbol{E}}'_\perp(\boldsymbol{k}_\perp) = \bar{A}(\alpha)\,\bar{Q}(\gamma)\,\bar{P}(\beta)\,\tilde{\boldsymbol{E}}_\perp(\boldsymbol{k}_\perp). \tag{106}$$

The longitudinal component is reconstructed after the analyzer from transversality,

$$\boxed{\tilde{E}'_z(\boldsymbol{k}_\perp) = -\frac{k_x\tilde{E}'_x(\boldsymbol{k}_\perp) + k_y\tilde{E}'_y(\boldsymbol{k}_\perp)}{k_z}, \qquad k_z = \sqrt{k^2 - k_\perp^2}} \tag{107}$$

which enforces $\boldsymbol{k}\cdot\tilde{\boldsymbol{E}}' = 0$ for each spectral component. Applied before the Jones elements, Eq. (107) reproduces the directly calculated Richards–Wolf E_z.

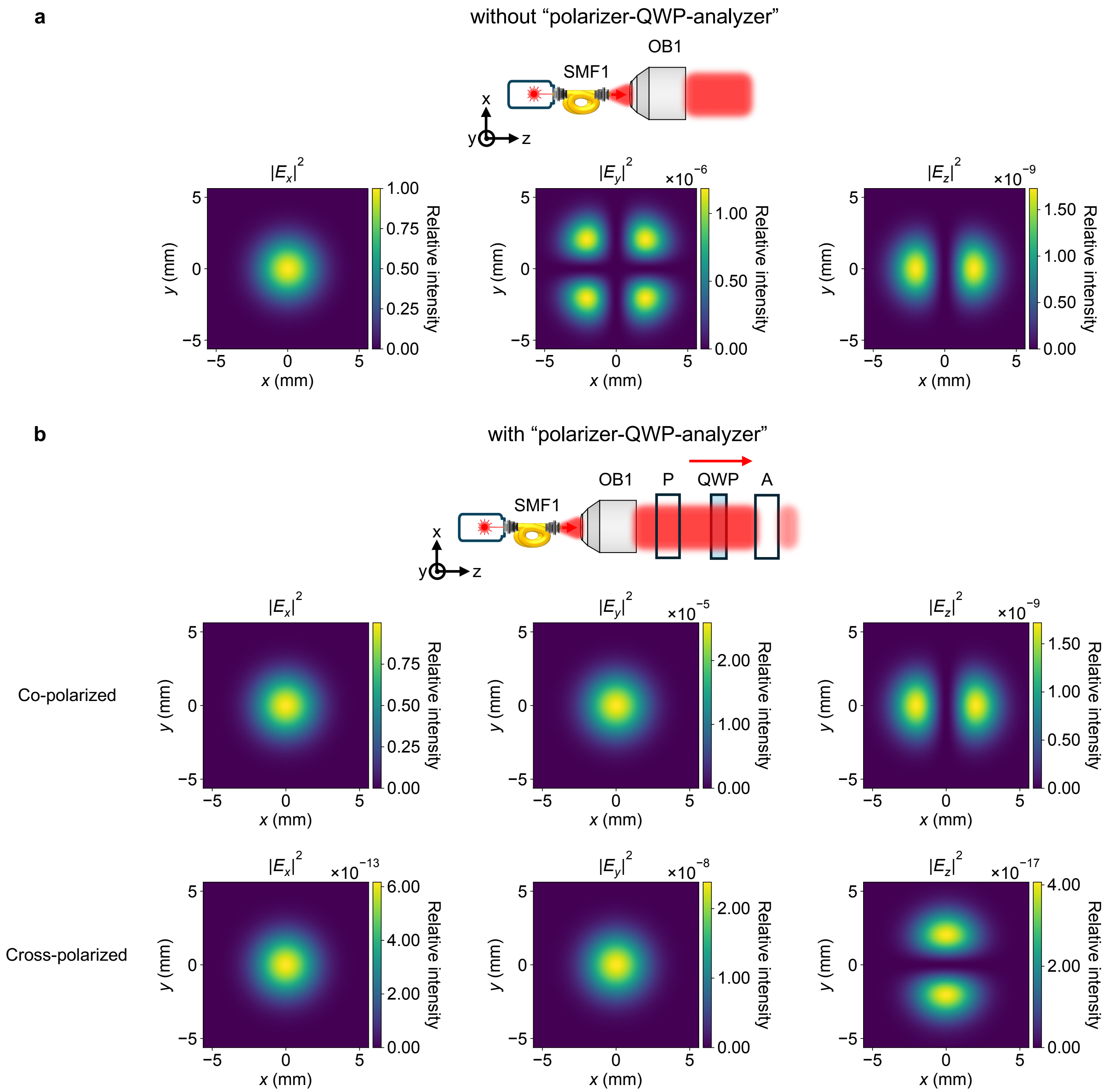

Supplementary Figure 14. **Vectorial Richards–Wolf simulation for a spatially uniform polarization chain. a**, Field after OB1 and before the polarizer–QWP–analyzer sequence. The dominant $|E_x|^2$ field is Gaussian; $|E_y|^2$ is a four-lobe transverse component with peak ratio 1.18×10^{-6}; and $|E_z|^2$ is a two-lobe longitudinal component with peak ratio 1.73×10^{-9}. **b**, Field after the polarizer, ideal QWP, and analyzer. The plane-wave extinction ratio is calibrated to 4.2×10^7. The upper and lower rows show the co- and cross-polarized outputs, normalized to the peak total intensity of the co-polarized output. In the cross-polarized channel, $|E_y|^2$ remains a node-free Gaussian with peak 2.38×10^{-8}, while $|E_x|^2$ and $|E_z|^2$ peak at 6.17×10^{-13} and 4.07×10^{-17}. The model produces no transverse first-order two-lobe mode.

In Supplementary Fig. 14b, the QWP fast axis is $\gamma = 0°$. We choose the effective polarizer angle β so that the optimized plane-wave extinction ratio equals 4.2×10^7, giving

$$\beta = 0.00884°, \qquad \alpha_{\text{co}} = -0.2063°, \qquad \alpha_{\text{cross}} = 89.7937°. \tag{108}$$

The analyzer settings are obtained from the maximum- and minimum-transmission eigenvectors of the plane-wave

coherency matrix. The extinction ratio alone cannot distinguish polarizer mismatch from analyzer offset; Eq. (108) therefore provides only an effective calibration of the residual transmission.

The upper and lower rows of Supplementary Fig. 14b show the co-polarized setting α_{co} and the cross-polarized setting α_{cross}, respectively. All six maps are normalized to the peak total intensity of the co-polarized output.

a. Co-polarized output. The dominant $|E_x|^2$ component is Gaussian and peaks at 0.99997. The weaker $|E_y|^2$ component has the same envelope and peaks at 2.59×10^{-5}. The longitudinal two-lobe component peaks at 1.72×10^{-9}.

b. Cross-polarized output. The dominant transverse intensity is the node-free Gaussian $|E_y|^2$ component, which peaks at 2.38×10^{-8}; $|E_x|^2$ peaks at 6.17×10^{-13}. The cross-to-co-polarized ratio integrated over the transverse plane is

$$\frac{I_{\mathrm{cross}}}{I_{\mathrm{co}}} = 2.38095\times10^{-8} = \frac{1}{4.2\times10^{7}}. \tag{109}$$

This value is fixed by the extinction-ratio calibration. The predicted cross-polarized profile retains the node-free Gaussian envelope of the co-polarized field. The only two-lobe component is longitudinal, with peak 4.07×10^{-17}; its axis rotates by $90°$ as the dominant transverse polarization changes from x to y.

H. Comparison with experiment

The extinction-ratio calibration fixes only the cross-polarized intensity scale; the spatial profile and field decomposition remain predictions.

1. No transverse first-order mode is generated

The measured cross-polarized pattern is a first-order two-lobe mode with a central node, and its orientation follows the QWP fast axis (Fig. 4 of the main text and Supplementary Fig. 12). The calculated transverse cross-polarized field is a node-free Gaussian. Equation (91) contains only $n = 0$ and $n = 2$ transverse orders; the $n = 1$ term is longitudinal. The intrinsic $n = 2$ component generated by OB1 is inversion-even and is strongly suppressed by the polarizer preceding the QWP. A spatially uniform Jones matrix cannot convert these fields into the measured transverse $n = 1$ mode.

2. The longitudinal first-order mode is negligible

For the present Gaussian beam, transversality gives $\max|E_z|^2/\max|E_\perp|^2 \simeq 1.7\times10^{-9}$. In the cross-polarized channel, the longitudinal peak is 4.07×10^{-17} of the co-polarized peak, more than nine orders of magnitude below the measured intensity of order 10^{-7}. Its orientation follows the transmitted polarization and rotates by $90°$ between the co- and cross-polarized settings, unlike the QWP-fast-axis-dependent transverse mode observed experimentally.

I. Conclusion

Finite-NA collimation produces an $n = 2$ transverse component with peak ratio 1.18×10^{-6} and an $n = 1$ longitudinal component with peak ratio 1.73×10^{-9}. After the calibrated spatially uniform polarization chain, the cross-polarized transverse profile remains Gaussian. Its integrated ratio of 2.38×10^{-8} is imposed by the extinction calibration, while the longitudinal two-lobe peak is only 4.07×10^{-17}. Intrinsic vectorial propagation with a spatially uniform QWP therefore cannot explain the observed transverse first-order mode. Such a mode requires a transverse-wave-vector- or real-space-dependent response, as developed in Supplementary Note 2 and Supplementary Note 3.

Supplementary Note 6. MACH–ZEHNDER INTERFEROMETRIC CHARACTERIZATION

We used a Mach–Zehnder interferometer to examine the transmitted-wavefront variations of the quartz and polymer zero-order QWPs at $\lambda = 633$ nm [Supplementary Fig. 15]. A collimated beam transmitted through the tested QWP was interfered with a reference beam, and the relative phase was recovered from the resulting interferogram. The measurement probes the scalar phase of the test-field component coherent with the reference beam.

Representative results are shown in Supplementary Fig. 16. Inserting either QWP modifies the fringe pattern and the extracted phase relative to the measurement without a QWP. Along each selected line cut, the phase is decomposed into a constant offset, a linear ramp, and a residual. The linear ramp is removed, and the remaining residual quantifies departures from a uniform wavefront tilt across the illuminated region. Supplementary Table 1 lists the fitted phase offset ϕ_0, linear phase gradient g_ϕ, and residual RMS σ_{res}. The measured residuals are 0.074 rad for the quartz QWP and 0.140 rad for the polymer QWP, corresponding to wavefront RMS values of approximately $\lambda/85$ and $\lambda/45$, respectively. These values are numerically below the transmitted-wavefront-error limits quoted by the vendors.

The measurements establish that the transmitted fields of both QWPs contain spatial phase variations across the illuminated footprint. Their smooth variation is compatible with the local first-order expansion used in Supplementary Note 3.

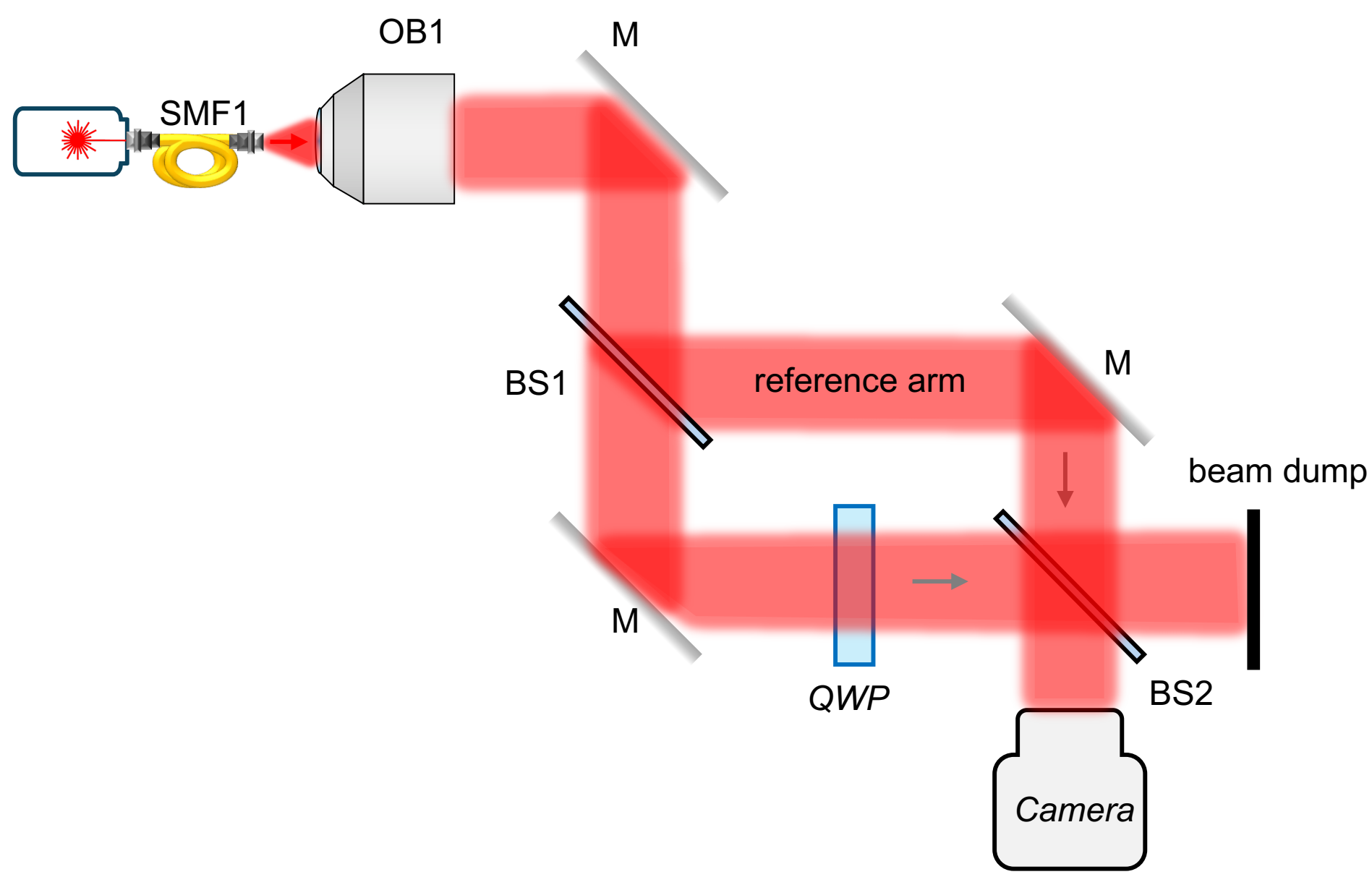

Supplementary Figure 15. **Mach–Zehnder interferometric measurement of the transmitted wavefront.** The tested QWP is placed in one arm of the interferometer. The transmitted test beam is recombined with a reference beam, and the resulting interferogram is recorded on a camera. The fringe phase provides the relative scalar phase across the illuminated region.

Supplementary Table 1. Mach–Zehnder phase metrics obtained from the line-cut analysis. The phase gradient is obtained from a linear fit to the phase relative to the reference interferogram. The residual RMS is evaluated after subtraction of this linear fit. The measurement without a QWP defines the phase reference.

Sample	ϕ_0 (rad)	g_ϕ (rad mm^{-1})	σ_{res} (rad)
Without QWP	–	–	–
Quartz zero-order QWP	-1.208	1.72×10^{-2}	0.074
Polymer zero-order QWP	-2.252	1.30×10^{0}	0.140

ϕ_0 is the wrapped phase intercept relative to the reference interferogram and depends on the chosen phase origin. $g_\phi = \partial_s \Delta\phi$ is the fitted phase gradient along the analyzed line cut. σ_{res} is the RMS phase residual after subtraction of the linear fit.

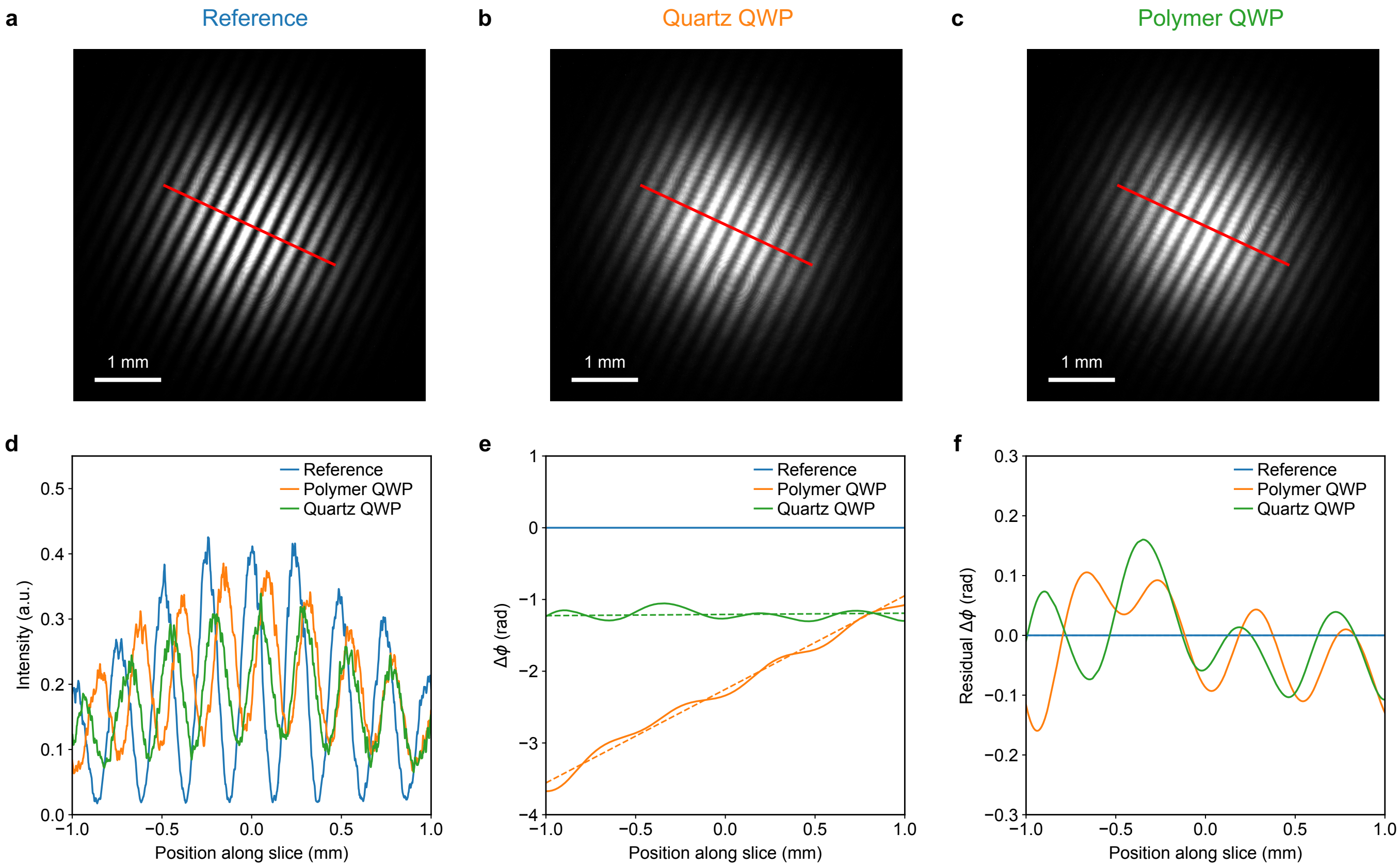

Supplementary Figure 16. **Transmitted-wavefront variations measured by Mach–Zehnder interferometry. a–c**, Representative interferograms measured without a QWP (**a**), with a quartz zero-order QWP (**b**), and with a polymer zero-order QWP (**c**). Red lines indicate the line cuts used for the phase analysis. **d**, Interference intensity along the selected line cuts. **e**, Relative phase $\Delta\phi$ recovered from the interferograms, using the measurement without a QWP as the reference. Dashed lines show the best linear fits. **f**, Residual phase after subtraction of the corresponding linear fit. The nonzero residuals quantify departures from a uniform phase ramp across the transmitted field.

Supplementary Note 7. FITTING PROCEDURE AND EXTRACTED PARAMETERS

The cross-polarized confocal maps are fitted with the first-order collected-field model of Eq. (57), corresponding to Eq. (6) of the main text. The matched-waist form in Eq. (59) is used only as a limiting case. The wavelength and fiber mode-field radius are fixed at $\lambda = 850$ nm and $\omega_{\mathrm{m}} = 2.5$ μm for the 850-nm measurements, and at $\lambda = 633$ nm and $\omega_{\mathrm{m}} = 2.1$ μm for the 633-nm measurements.

For each dataset, the co-polarized map is first fitted with the Gaussian fiber-overlap model to determine the focused-beam radius ω_f and beam center (x_0, y_0). Initial values of η_0, G_x, and G_y are obtained from the central signal and a linearized fit to the square root of the cross-polarized intensity. The full map is then fitted for η_0, G_x, and G_y, together with an additive background and small transverse offsets. The analyzer-dependent maps are fitted jointly with an affine dependence on the analyzer detuning $\delta\alpha$; the beam-size- and fast-axis-dependent maps are fitted independently. The optimization uses square-root-intensity residuals and a robust loss within the selected fit region. The error metrics reported below are evaluated on the linear intensity over all valid pixels. Only $|\eta_0|$, G_x^{fit}, and G_y^{fit} are tabulated. In the notation of Eq. (57), the reported coefficients are

$$G_{x,y}^{\mathrm{fit}} = \Lambda G_{x,y}, \qquad \Lambda = \frac{\omega_f^2}{\omega_f^2 + \omega_{\mathrm{m}}^2}. \tag{110}$$

Let I_i^{exp} and I_i^{fit} denote the measured and fitted intensities at the ith valid pixel, and let N be the number of valid pixels. We report the range-normalized root-mean-square error

$$\mathrm{NRMSE}_{\mathrm{range}} = \frac{\sqrt{N^{-1}\sum_i \left(I_i^{\mathrm{fit}} - I_i^{\mathrm{exp}}\right)^2}}{I_{\mathrm{max}}^{\mathrm{exp}} - I_{\mathrm{min}}^{\mathrm{exp}}}, \tag{111}$$

and the relative L^2 error

$$\mathrm{RelErr}_{L^2} = \sqrt{\frac{\sum_i \left(I_i^{\mathrm{fit}} - I_i^{\mathrm{exp}}\right)^2}{\sum_i \left(I_i^{\mathrm{exp}}\right)^2}}. \tag{112}$$

Across the datasets in Supplementary Tables 2–5, $\mathrm{NRMSE}_{\mathrm{range}}$ ranges from 0.017 to 0.059, while RelErr_{L^2} ranges from 0.066 to 0.210.

At the fitted origin, the first-order terms vanish and the background-subtracted cross-to-co-polarized intensity ratio is $|\eta_0|^2$. The corresponding model extinction ratio is therefore $|\eta_0|^{-2}$. In Supplementary Table 2, $|\eta_0| = 1.54 \times 10^{-4}$ at $\delta\alpha = 0$ gives 4.22×10^7, while the values at $\delta\alpha = -0.16^\circ$ and $+0.16^\circ$ give 1.50×10^5 and 1.43×10^5, respectively. These values are consistent with the independently measured extinction-ratio curve.

For the beam-size series, $|\eta_0|$ lies between 1.67×10^{-4} and 1.94×10^{-4}, whereas

$$|\boldsymbol{G}^{\mathrm{fit}}| = \left(|G_x^{\mathrm{fit}}|^2 + |G_y^{\mathrm{fit}}|^2\right)^{1/2} \tag{113}$$

increases from 0.0508 to 0.146 mm^{-1}. With $\omega_f = \lambda f/(\pi\omega_0)$, the spatial and angular first-order channels predict

$$|\boldsymbol{G}_{(r)}^{\mathrm{fit}}| \propto \Lambda(\omega_0)\omega_0^2, \qquad |\boldsymbol{G}_{(\Theta)}^{\mathrm{fit}}| \propto \Lambda(\omega_0), \tag{114}$$

respectively. After normalization to the smallest-beam point, the measured magnitude increases from 1.00 to 2.87. The spatial prediction increases to 2.82, with a maximum pointwise difference of 11.4%, whereas the angular prediction decreases to 0.61. Within these two first-order channels, the fitted-coefficient trend is consistent with the spatial-gradient scaling and opposite to the angular-only scaling.

Supplementary Table 2. Fitted parameters for the analyzer-dependent measurements in Fig. 2 of the main text.

$\delta\alpha$ (°)	$\lvert\eta_0\rvert$	G_x^{fit} (mm^{-1})	G_y^{fit} (mm^{-1})	$\mathrm{NRMSE}_{\mathrm{range}}$	RelErr_{L^2}
−0.16	2.58×10^{-3}	$0.180 - 0.080\,\mathrm{i}$	$0.073 + 0.358\,\mathrm{i}$	0.017	0.066
−0.08	1.28×10^{-3}	$0.094 - 0.098\,\mathrm{i}$	$0.045 + 0.177\,\mathrm{i}$	0.018	0.067
+0.00	1.54×10^{-4}	$0.008 - 0.116\,\mathrm{i}$	$0.017 - 0.004\,\mathrm{i}$	0.037	0.111
+0.08	1.34×10^{-3}	$-0.078 - 0.133\,\mathrm{i}$	$-0.011 - 0.186\,\mathrm{i}$	0.020	0.076
+0.16	2.64×10^{-3}	$-0.165 - 0.151\,\mathrm{i}$	$-0.039 - 0.367\,\mathrm{i}$	0.020	0.072

Supplementary Table 3. Fitted parameters for the beam-size-dependent measurements in Fig. 3 of the main text.

ω_0 (mm)	$\|\eta_0\|$	G_x^{fit} (mm^{-1})	G_y^{fit} (mm^{-1})	NRMSE$_{\mathrm{range}}$	RelErr$_{L^2}$
1.301	1.67×10^{-4}	$-0.003 + 0.032\,\mathrm{i}$	$-0.018 + 0.035\,\mathrm{i}$	0.039	0.131
1.570	1.75×10^{-4}	$-0.007 + 0.046\,\mathrm{i}$	$-0.021 + 0.050\,\mathrm{i}$	0.031	0.128
1.818	1.67×10^{-4}	$-0.010 + 0.057\,\mathrm{i}$	$-0.035 + 0.065\,\mathrm{i}$	0.025	0.119
2.115	1.69×10^{-4}	$-0.014 + 0.068\,\mathrm{i}$	$-0.045 + 0.080\,\mathrm{i}$	0.023	0.126
2.424	1.76×10^{-4}	$-0.011 + 0.082\,\mathrm{i}$	$-0.050 + 0.097\,\mathrm{i}$	0.025	0.151
2.795	1.94×10^{-4}	$-0.010 + 0.081\,\mathrm{i}$	$-0.071 + 0.098\,\mathrm{i}$	0.028	0.210

Supplementary Table 4. Normalized beam-size dependence of the fitted first-order coefficient magnitude and the two first-order channel predictions.

ω_0 (mm)	$\|\boldsymbol{G}^{\mathrm{fit}}\|/\|\boldsymbol{G}^{\mathrm{fit}}\|_1$	$[\Lambda\omega_0^2]_{\mathrm{norm}}$	Λ_{norm}	relative difference (%)
1.301	1.00	1.00	1.00	0.0
1.570	1.41	1.35	0.93	4.3
1.818	1.85	1.67	0.86	10.4
2.115	2.27	2.05	0.78	10.5
2.424	2.70	2.42	0.70	11.4
2.795	2.87	2.82	0.61	1.9

All columns are normalized to the smallest-beam point. The spatial and angular predictions are $\Lambda\omega_0^2$ and Λ, respectively, with $\Lambda = \omega_f^2/(\omega_f^2 + \omega_{\mathrm{m}}^2)$ and $\omega_f = \lambda f/(\pi\omega_0)$. The last column is the absolute relative difference between the measured normalized magnitude and $[\Lambda\omega_0^2]_{\mathrm{norm}}$.

Supplementary Table 5. Fitted parameters for the fast-axis-dependent measurements in Fig. 4 of the main text.

Polarizer	QWP fast axis	$\|\eta_0\|$	G_x^{fit} (mm^{-1})	G_y^{fit} (mm^{-1})	NRMSE$_{\mathrm{range}}$	RelErr$_{L^2}$
0°	0°	1.54×10^{-4}	$0.008 - 0.116\,\mathrm{i}$	$0.017 - 0.004\,\mathrm{i}$	0.037	0.111
0°	90°	1.60×10^{-4}	$0.030 - 0.011\,\mathrm{i}$	$0.050 - 0.137\,\mathrm{i}$	0.043	0.138
90°	90°	1.51×10^{-4}	$0.016 - 0.001\,\mathrm{i}$	$0.008 + 0.101\,\mathrm{i}$	0.044	0.129
90°	0°	1.18×10^{-4}	$0.055 + 0.136\,\mathrm{i}$	$-0.047 - 0.032\,\mathrm{i}$	0.051	0.170
45°	45°	1.51×10^{-4}	$0.018 - 0.090\,\mathrm{i}$	$-0.013 - 0.100\,\mathrm{i}$	0.045	0.136
45°	-45°	1.37×10^{-4}	$-0.042 + 0.060\,\mathrm{i}$	$-0.001 - 0.111\,\mathrm{i}$	0.059	0.196
-45°	-45°	1.44×10^{-4}	$-0.016 + 0.078\,\mathrm{i}$	$-0.018 - 0.074\,\mathrm{i}$	0.037	0.105
-45°	45°	1.57×10^{-4}	$0.108 + 0.073\,\mathrm{i}$	$0.032 + 0.094\,\mathrm{i}$	0.042	0.135

Supplementary Note 8. VENDOR SPECIFICATIONS AND ANGLE-OF-INCIDENCE RETARDANCE DATA

Supplementary Table 6 summarizes the vendor specifications relevant to the first-order spatial channel [10]; they provide reference scales for Supplementary Note 3 and Supplementary Note 6. The polymer retardance-uniformity specification of < 5 nm RMS corresponds to an RMS retardance variation below 4.96×10^{-2} rad at 633 nm and 3.70×10^{-2} rad at 850 nm.

Supplementary Table 6. Vendor specifications of the tested QWP product families (633 nm products quoted; from Thorlabs [10]). The 850 nm plates used in the main experiment (WPQ05M-850 and WPQ10E-850-SP) belong to the same product families; the wavefront-distortion and retardance-uniformity values are quoted by the vendor at the family level.

	Quartz zero-order (WPQ05M-633)	Polymer zero-order (WPQ10E-633)	Quartz multi-order (WPMQ05M-633)
Retardance accuracy	$< \lambda/300$	$< \lambda/100$	$< \lambda/100$ (typ.)
Retardance uniformity (RMS)	—	< 5 nm	—
Transmitted wavefront error	$\lambda/8$	$< \lambda/4$	$\lambda/10$ (RMS)
Beam deviation	< 10 arcsec	< 5 arcmin	< 10 arcsec
Surface quality (scratch-dig)	20-10	60-40	20-10

To provide an external reference for the angular response of different QWP materials, we summarize vendor-provided retardance data from Thorlabs for a quartz plate (Supplementary Table 7) and a liquid-crystal-polymer plate (Supplementary Table 8). The retardance variation with angle of incidence (AOI) is strongly material- and device-dependent. The quartz data are the source of the parabolic coefficient $a \simeq 33$ rad^{-1} used in Eq. (70); the polymer data show that the same plate family is essentially flat over $\pm 10°$. These values are used as order-of-magnitude references for the angular contribution discussed in Supplementary Note 3 F and in the main text.

Supplementary Table 7. Vendor-provided angle-dependent retardance of a quartz quarter-wave plate from Thorlabs[10]. AOI is given in degrees and retardance in waves.

AOI (degree)	Retardance (waves)	AOI (degree)	Retardance (waves)
-4.5	0.2170	1.5	0.2444
-3.5	0.2286	2.5	0.2374
-2.5	0.2359	3.5	0.2290
-1.5	0.2435	4.5	0.2156
-0.5	0.2500	5.5	0.1974
0.5	0.2498	6.5	0.1757
1.0	0.2473		

Supplementary Table 8. Vendor-provided angle-dependent retardance of a liquid-crystal-polymer quarter-wave plate, WPQ10E-633, from Thorlabs[10]. AOI is given in degrees and retardance in waves.

AOI (degree)	Retardance (waves)	AOI (degree)	Retardance (waves)
-50.0	0.2081	0.5	0.2499
-40.0	0.2238	1.5	0.2495
-30.0	0.2354	2.5	0.2491
-20.0	0.2437	3.5	0.2488
-10.0	0.2483	4.5	0.2485
-5.5	0.2486	5.5	0.2484
-4.5	0.2488	10.0	0.2463
-3.5	0.2491	20.0	0.2414
-2.5	0.2492	30.0	0.2338
-1.5	0.2495	40.0	0.2254
-0.5	0.2498	50.0	0.2164
0.0	0.2500		

Supplementary References

[1] M. Benelajla, E. Kammann, B. Urbaszek, and K. Karrai, "Physical origins of extreme cross-polarization extinction in confocal microscopy," *Physical Review X* **11**, 021007 (2021).

[2] K. Y. Bliokh and A. Aiello, "Goos–Hänchen and Imbert–Fedorov beam shifts: an overview," *Journal of Optics* **15**, 014001 (2013).

[3] K. Y. Bliokh, C. T. Samlan, C. Prajapati, G. Puentes, N. K. Viswanathan, and F. Nori, "Spin-Hall effect and circular birefringence of a uniaxial crystal plate," *Optica* **3**, 1039–1047 (2016).

[4] K. Y. Bliokh, C. Prajapati, C. T. Samlan, N. K. Viswanathan, and F. Nori, "Spin-Hall effect of light at a tilted polarizer," *Optics Letters* **44**, 4781–4784 (2019).

[5] N. Modak, S. Das, P. Bordoloi, and N. Ghosh, "Tunable giant two-dimensional optical beam shift from a tilted linear polarizer," *Physical Review A* **105**, 033713 (2022).

[6] L. Novotny and B. Hecht, *Principles of Nano-Optics*, 2nd ed. (Cambridge University Press, Cambridge, 2012).

[7] M. Born and E. Wolf, *Principles of Optics*, 7th ed. (Cambridge University Press, Cambridge, 1999).

[8] E. Wolf, "Electromagnetic diffraction in optical systems. I. An integral representation of the image field," *Proc. R. Soc. Lond. A* **253**, 349–357 (1959).

[9] B. Richards and E. Wolf, "Electromagnetic diffraction in optical systems. II. Structure of the image field in an aplanatic system," *Proc. R. Soc. Lond. A* **253**, 358–379 (1959).

[10] Thorlabs Inc., "Comparison between the performance of a WPQ10M-633 quartz quarter-wave plate and a WPQ10E-633 liquid crystal polymer quarter-wave plate with respect to angle of incidence," https://www.thorlabs.com/polymer-zero-order-quarter-wave-plates?tabName=Overview.
