# Supplementary material for: Real-space spin-orbit interaction from quarter-wave plate nonuniformity
Source: Nat Commun. 2026 Sep 9;17:9653. doi: 10.1038/s41467-026-77581-2 (PMC13558598; doi:10.1038/s41467-026-77581-2)
Supplement: Supplementary file 2 [file 41467_2026_77581_MOESM2_ESM.pdf]

nature portfolio

Peer Review File

Real-space spin–orbit interaction from quarter-wave plate nonuniformity

Corresponding Author: Professor Bernhard Urbaszek

This file contains all reviewer reports in order by version, followed by all author rebuttals in order by version.

Version 0:

Reviewer comments:

Reviewer #1

(Remarks to the Author)
The manuscript reports that inserting a quarter-wave plate (QWP) in a simple confocal geometry can enhance cross-polarization extinction and transform a Gaussian beam into a first-order Hermite–Gaussian-like two-lobe pattern. The authors attribute this effect to spin–orbit interaction and propose a phenomenological extension of Jones matrix formalism to reproduce the measured spatial profiles. While experimental observation is interesting and may be useful for optimizing polarization rejection in confocal microscopy, I do not find the manuscript suitable for Nature Communications.
My main concern is that the central physical mechanism is not convincingly established. The authors repeatedly acknowledge that their model is phenomenological, that the fitted parameter magnitude is far from the expected theoretical scale, and that the observed lobe intensities are “several orders of magnitude stronger than theory predicts”. This is a serious weakness rather than a minor open question. Without a quantitatively predictive model, it is difficult to support the claim that the observed mode conversion represents a previously overlooked form of spin–orbit-mediated mode control, rather than an artifact arising from waveplate imperfections, slight misalignment, wedge, retardance mismatch, fiber-mode filtering, or residual astigmatism.
The novelty also appears limited for Nature Communications. Spin–orbit effects induced by anisotropic plates, tilted polarizers, and confocal cross-polarization geometries have been studied previously, including the mirror-based confocal extinction work that the authors themselves cite. The present manuscript replaces or supplements such elements with a QWP, but the advance is mainly an experimental observation in a specific optical setup rather than a broadly generalizable principle. The claimed applications in resonant spectroscopy, imaging, quantum optics, and mode engineering are speculative and not demonstrated.
Several technical points require substantial clarification. First, the role of the single-mode fiber is central, but the separation between true beam reshaping and detection-mode filtering is not rigorously quantified. Second, the QWP imperfections are invoked as a possible origin of the effect, yet no systematic comparison between different QWPs, retardance values, wavelengths, or incidence angles are provided. Third, the extinction enhancement is only about, lower than previously reported mirror-based configurations, so the practical advantage is not compelling. Fourth, the fitting procedure uses effective complex parameters without a clear physical origin, making the agreement between experiment and fit insufficient as evidence for the proposed mechanism. Finally, the manuscript would benefit from direct wavefront/phase characterization rather than intensity-only modal maps.
Overall, the work reports a potentially useful laboratory observation, but the physical interpretation remains incomplete and the conceptual advance is not strong enough for Nature Communications. I therefore recommend rejection.

Reviewer #2

(Remarks to the Author)
The authors investigate a seemingly simple confocal setup, where light from one single-mode fiber is collimated, sent through polarizing components, and coupled into the receiving single-mode fiber. They find that introducing a QWP between two polarizers, and optimizing the angles, polarization extiction can be improved by 2 orders of magnitude compared to the case of only two polarizers. By spatial scanning the receiving SMF, as well as recording the collimated field with a camera, they find that a HG10-like structure appears, and since the overlap with the Gaussian fiber mode vanishes, this enhances the cross-polarization contrast.
The authors show a nice derivation of a Fourier-optics model based on previous theoretical studies (in particular in the

supplemental), and find good qualitative agreement to the data using some fit parameters.
I have some comments:

* Definition of angles and sketches:
- fig.1a: the arced arrow indicating angles start at the origins, unclear to me (I guess 0 deg is at the right horizontal axis, isn't it?).
- definition of $\delta\alpha$: did I miss it, can't find in text or supplemental? I only read "$\delta\alpha$ around the symmetrically split mode." - can this be made precise or is this it?

* Fig. S0: I see a QWP being illuminated from the side (i.e., rotated by 90 degrees around the x-axis compared to normal use) - I can't believe that this is intended - also Fig. S4 shows the expected case, or am I missing something?

* The HG10 pattern and its angular orientation, the discussion thereof I find a bit descriptive - because of the clear origin, the spatial derivatives in the formulae, in addition to the retardation changes with tilts, I would say that a more intuitive explanation should be possible! I read only "This behaviour is naturally understood as a polarization-dependent reorientation of the transverse field distribution.", which probably doesn't satisfy many readers!

* About the mystery of high intensity of the cross-polarized HG10 field, mentioned at the very end of the manuscript ("both experimental sets fail to account for the lobe intensities, which are measured to be several orders of magnitude stronger than theory predict"):
- I can't find any data about this - adding details of how much would be nice!
- So, either the model is wrong, or, IMO more likely, some or more assumptions about the experiment are wrong. For nat.commun, I find a few tests are suitable:
- An obvious attempt, which I don't see in the manuscript, is exchanging optical components, in particular the QWP and the light source.
- The laser type is not specified - but even with excellent single-frequency diode lasers, the sidebands often carry 1e-5 of the laser intensity, which might lead to dispersive issues. (HeNe?)
- They QWP is composed of two multi-order plates made from quartz - I think trying a different model, or a low-order simpler QWP, would be not too hard. And despite the plate has anti-reflection coatings (which might be strained), multiple reflections do occur.
- More crazy, but could the (small) optical activity of quartz play a role? It would be easy to test MgF_2 QWPs, they should not show optical activity or circular dichroism.
From personal experience I know that quantitative agreement is pretty hard at the 1e-5 level, but even an indication where the issue is (by observing changes in the intensity) would increase trust in the model.

Reviewer #3

(Remarks to the Author)
In the manuscript “Role of a Quarter-Wave Plate in Confocal Microscopy: Signature of Spin-Orbit Interactions,” Wenze Lan et al. demonstrate a mode transformation in a paraxial beam obtained by collimating a nearly diffraction-limited Gaussian beam (λ = 850 nm and ω_0 =2.5 μm) using a microscope objective (NA = 0.25). The authors present solid experimental evidence of this mode transformation when a quarter-wave plate is placed between two crossed polarizers. This phenomenon is indeed unexpected in paraxial optics, where polarizers and quarter-wave plates are not typically associated with spatial mode transformations.
Although most of the measurements were performed by refocusing and coupling the collimated beam into an optical fiber, the authors also provide, in the supplementary material, images acquired with a camera placed in the collimated portion of the beam. They propose a modification of the Jones matrix of the quarter-wave plate to account for this phenomenon.
I have one comment regarding one of the main assumptions of the paper, namely the assumption that non-paraxial effects are negligible.
1. Since the source is a nearly diffraction-limited Gaussian beam, I would recommend computing the full solution of Maxwell’s equations for such a beam in order to account for the longitudinal field component. One should then model the subsequent collimation of this field by a microscope objective using the Richards–Wolf formalism for a lens of arbitrary numerical aperture.
I would be in favor of publication of this paper as it stands if the authors could provide simulations that retain the standard Jones matrix for the quarter-wave plate while properly accounting for the non-paraxial nature of the nearly diffraction-limited Gaussian beam and its collimation by a 0.25 NA objective, and demonstrate that this model (i.e., a full solution of Maxwell’s equations) is still insufficient to account for the observed mode transformations.
The experimental results are interesting, as they could potentially indicate that the boundary between paraxial and non-paraxial optics is not as well defined as commonly assumed. In some works, non-paraxial effects are considered to become relevant for objectives with NA > 0.5 or NA > 0.6. However, in regimes where intensity differences on the order of 5 to 7 orders of magnitude can be measured and are relevant, as shown in Fig. 1(c) and 1(d), these distinctions may become less clear. Alternatively, these experimental results may demonstrate that it is indeed necessary to consider a modification of the Jones matrix of the quarter-wave plate.

Reviewer #4

(Remarks to the Author)
The paper reports an interesting effect associated with spin orbit interaction (SOI) of light beam in a uniaxial anisotropic

medium (waveplate) in confocal microscopy geometry. The authors have experimentally demonstrated SOI effect of fundamental Gaussian beam of light in confocal detection geometry when a quarter waveplate (QWP) is kept between crossed liner polarizers, leading to reshaping of the transverse field structure of the input Gaussian beam. They also demonstrated enhancement of extinction ratio of the crossed polarizers due to this SOI effect. The results are supported by corresponding theoretical formalism of SOI of light in uniaxial birefringent medium (waveplate). Based on these, the authors claim the novelty of this SOI effect in anisotropic medium observed in confocal geometry. SOI of light beams in various light matter interactions like partial and total internal reflection in dielectric interfaces, in tight focusing, in scattering and in inhomogeneous anisotropic (both birefringent and dichroic) medium, metasurfaces etc. has been widely explored in recent times, which have opened up exciting new avenues for controlled manipulation of light's field distribution using angular momentum degree of freedom of light and for the development of spin and orbital angular momentum-controlled photonic devices and systems. This work on SOI of light in uniaxial birefringent medium in confocal geometry is certainly timely and interesting. My main concern is on the novelty of the observed SOI effect and its fundamental origin in uniaxial anisotropic (birefringent) medium, which has been studied before (and the corresponding theoretical foundation is also well established) as I have noted down below. That said, I do agree observation of SOI in confocal geometry using a simple polarizer-QWP-polarizer combination and the resulting control on the spatial distribution of the field and the significant enhancement extinction ratio through SOI is expected to have useful applications.

The basic findings of the study can be fully explained using previously developed formalisms of SOI of light beam (having transverse wave vectors k_(x and) k_y) in uniaxial anisotropic medium exhibiting either linear diattenuation or dichroism (like polarizer) or linear retardance (waveplate) (OPTICA, 3, 1039, 2016, Optics Letters, 19, 4781, 2019 and Phys Rev A, 105, 033713, 2022). Unlike plane waves, for a light beam the polarization response of the anisotropic medium can be described by momentum domain Jones matrix J(k_(x/y)), which can be expanded around the zeroth order matrix (J(k_(x/y)=0), corresponding to the plane wave situation). This results in both in-plane (k_(x)) and out of plane (k_y) wave vector-dependent additional terms in the diagonal (k_x-dependent) and in the off-diagonal (k_y-dependent) elements of the Jones matrix. The latter k_y-dependent modification of the input field distribution is related to SOI of light as a consequence of the generation of k_y-dependent PB geometric phase of light. Once the symmetry of the uniaxial anisotropic medium is broken (e.g. by introducing slight tilt), it results in the well-known Spin Hall or Imbert-Fedorov (IF) shifts (having eigen polarization states of circular / elliptical polarizations). The k_x-dependent modification of the input field on the other hand leads to analogous Goose-Hanchen (GH) shift. In fact, these formalisms presented in the papers mentioned above have one to one correspondence to the usual transverse IF and longitudinal GH shift matrices (which can be directly derived from J(k_(x/y))) obtained for partial or total internal reflection at dielectric interfaces having very similar origin. Note that Eq. 1-5 presented in the current manuscript can also be directly derived from this formalism. Here the authors have investigated a QWP (having linear retardance and no diattenuation) and in this scenario one is concerned about the k_y-dependent modification of the field and the resulting transverse Spin Hall shift. Now in this scenario when a crossed polarizer is inserted, this will act like a standard weak measurement experiment of Spin Hall shift with exact orthogonal pre-post selection of polarization states. It is well known that in such scenario (of exact orthogonal pre-post selection using crossed-linear polarizer) one obtains Hermite-Gaussian like two lobe field structure, which has been observed by the authors. As has also been demonstrated earlier (Phys Rev A, 105, 033713, 2022), the symmetry here is decided by the orientation axis of the anisotropic medium (here the QWP), and thus the field structure can be rotated by rotating the anisotropy axis. Thus, the results obtained in this study can be completely explained by the already existing formalism of SOI of light in anisotropic medium with broken symmetry. The authors therefore need to justify and provide concrete evidence of the novelty of their work in light of the above discussion. In my understanding the only new findings is observation of the same effect in confocal arrangement, which of course will have practical applications. But whether that alone represent sufficient novelty is debatable.

I also have the following minor comments / suggestions –
The introduction section and also the theory section does not adequately bring out the existing work in the domain of SOI of light in uniaxial anisotropic medium (the works mentioned above and other related work). Specifically, the theory section is misleading and the authors should clearly present the existing models of momentum domain Jones matrix formalism as discussed above. They should also highlight if there is any new theoretical foundations of this effect based on the already existing models in literature.

The SOI effect arises from symmetry breaking in the QWP (like introducing small tilt in previous papers). The authors state that in their experiment symmetry breaking arises from imperfections of the QWP. This appears a little vague. The authors need to provide exact mechanisms of symmetry breaking and a quantitative description of the same (like in previous papers results have always been presented in terms of the tilt angle).

In summary, I have noted my main concerns on the novelty of the foundation of the observed SOI effect of light beam in anisotropic medium, which the authors must address before it can be considered further. Otherwise, the results appear interesting, analysis and the methodology appears to be neat, the interpretations are also based on the underlying physical basis. The paper is clearly written and the results are presented in logical manner.

Version 1:

Reviewer comments:

Reviewer #1

(Remarks to the Author)
The authors have properly addressed all comments raised by me and other reviewers. Generally I am satisfied with the revisions. Therefore, I would recommend its publication.

Reviewer #2

(Remarks to the Author)
The authors have done additional experiments and extended their model and done full vectorial calculations. The authors now were able to find an explanation of the magnitude of the observed effects - spatial variations of the QWP, taken now into account by the real-space variation in Eq. 5 and explained in S3. One might say that it is obvious that spatial variations enter via higher-order spatial modes, but I didn't made this comments before and I find it surprising that they "amplify" the angular effects in the way the authors found now (which could be emphasized/explained more in the manuscript).
The new experiments with varied beam collimation in Fig. 3, which has opposite effects on angular spread and beam size, are quite convincing. As a side-product, the authors found a new method to quantify uniformity of QWPs. The authors also did tests with different waveplates and light sources, making this in my opinion a complete story - with pretty new conclusions: The effect is mainly not due to angular but spatial effects, with very similar manifestations. I wonder how much this new insight changes the scientific charme of the manuscript. Probably, because the story is still conceptually nice and connects angular and spatial effects in one framework, this could still be seen to be similarly compelling as before.
One point that might be interesting could be to analyze related precision experiments, for instance on beam shifts, or if the effects only appear in the particular geometry here.

Reviewer #3

(Remarks to the Author)
The authors have addressed the main comments and improved the overall structure of the paper. They have performed calculations based on a full vectorial model that verify that the mode transformation is not related to non-paraxial effects. They presented their results in Supplementary Note S5. I therefore agree with the publication of the manuscript.

Minor comments:

1. In Fig. S13, the authors define $\theta'=\omega_0/z_r$, while in Eq. S79 they use $\theta'=\omega_0/l$. Please homogenize the notation.

Reviewer #4

(Remarks to the Author)
The authors have done a satisfactory job in addressing several of my concerns on the nature of SOI in tilted waveplate and the resemblance to the already established frameworks on SOI in such scenario and also to the standard weak measurement scenario related to beam shifts with near orthogonal pre-post selection of polarization states. Specifically, they have established the novelty of the present work by providing additional theoretical analysis, experimental results with proper citation to previous work that i had noted in my previous report. I can now recommend the revised manuscript for publication.

Open Access This Peer Review File is licensed under a Creative Commons Attribution 4.0 International License, which permits use, sharing, adaptation, distribution and reproduction in any medium or format, as long as you give appropriate credit to the original author(s) and the source, provide a link to the Creative Commons license, and indicate if changes were made.
In cases where reviewers are anonymous, credit should be given to 'Anonymous Referee' and the source.
The images or other third party material in this Peer Review File are included in the article's Creative Commons license, unless indicated otherwise in a credit line to the material. If material is not included in the article's Creative Commons license and your intended use is not permitted by statutory regulation or exceeds the permitted use, you will need to obtain permission directly from the copyright holder.
To view a copy of this license, visit https://creativecommons.org/licenses/by/4.0/

Point-by-Point Response to the Reviewers

Manuscript NCOMMS-26-028643-T

Revised title: Real-space spin–orbit interaction from quarter-wave plate nonuniformity

Previous title: Role of a Quarter-Wave Plate in Confocal Microscopy: Signature of Spin–Orbit Interactions

Summary of Revisions. We thank all four reviewers for their careful reading and constructive feedback. Their comments led us to identify and resolve the central open questions of the original submission — the unexplained intensity scale of the cross-polarized two-lobe mode — and to substantially strengthen both theoretical and experimental conclusions. The main revisions are:

- A revised first-order model of the quarter wave plate (QWP) response that includes both angular (momentum-domain) and real-space (spatial-gradient) corrections, resolving the previously unexplained intensity discrepancy (revised Eqs. (5)–(9) of the main text; Supplementary Notes S2 and S3).
- New beam-size-dependent control experiments that discriminate between the angular and spatial mechanisms through the *sign* of the trend, and identify the real-space channel as dominant (new Fig. 3 of the main text; Supplementary Figs. S6–S9; Supplementary Table S4).
- A full vectorial Maxwell + Richards–Wolf calculation ruling out non-paraxial propagation with an ideal QWP as the origin of the observed mode transformation (Supplementary Note S5, Figs. S13–S14).
- Explicit acknowledgment and integration of the established momentum-domain Jones-matrix framework (Bliokh *et al.*, Optica 3, 1039 (2016); Opt. Lett. 44, 4781 (2019); Modak *et al.*, Phys. Rev. A 105, 033713 (2022)) in the introduction and theory section.
- Additional control experiments (Supplementary Notes S4, S6 and S8) show the validity of our revised theoretical model:
  - different QWP materials (quartz, polymer, $\mathrm{MgF_2}$)
  - different QWP retardance orders (zero-order, multi-order),
  - two wavelengths and laser sources (850 nm diode, 633 nm HeNe),
  - tilt-angle sweeps,
  - direct camera imaging,
  - and Mach–Zehnder interferometric wavefront measurements.

All changes in the revised manuscript and Supplementary Materials are marked in blue. Below, reviewer comments are reproduced in shaded boxes, followed by our responses. All figure and note numbers refer to the revised version.

Reviewer #1

We thank Reviewer #1 for the thorough and critical assessment. The concerns raised were instrumental in identifying the main open questions of the previous version: the absence of a sufficiently quantitative physical model. As detailed below, the revised manuscript replaces the phenomenological description with a predictive first-order theory, validated by new control experiments.

General comment

> *Reviewer comment: The manuscript reports that inserting a quarter-wave plate (QWP) in a simple confocal geometry can enhance cross-polarization extinction and transform a Gaussian beam into a first-order Hermite–Gaussian-like two-lobe pattern. The authors attribute this effect to spin–orbit interaction and propose a phenomenological extension of Jones matrix formalism to reproduce the measured spatial profiles. While experimental observation is interesting and may be useful for optimizing polarization rejection in confocal microscopy, I do not find the manuscript suitable for Nature Communications.*

Response:

In the revised manuscript, we have substantially reworked both the theoretical framework and the experimental validation. Specifically, we now (i) include first-order spatial (real-space) corrections of the QWP in addition to the angular (momentum-domain) corrections; (ii) present beam-size-dependent measurements across multiple QWP materials that directly discriminate between the two mechanisms; (iii) add Mach–Zehnder interferometric wavefront measurements; and (iv) revise the wording throughout to clearly separate demonstrated results from speculative applications. With these additions, the model accounts for both the two-lobe spatial structure and the measured intensity scale.

Major concern 1 — Physical mechanism not convincingly established

> *Reviewer comment: My main concern is that the central physical mechanism is not convincingly established. The authors repeatedly acknowledge that their model is phenomenological, that the fitted parameter magnitude is far from the expected theoretical scale, and that the observed lobe intensities are "several orders of magnitude stronger than theory predicts". This is a serious weakness rather than a minor open question. Without a quantitatively predictive model, it is difficult to support the claim that the observed mode conversion represents a previously overlooked form of spin–orbit-mediated mode control, rather than an artifact arising from waveplate imperfections, slight misalignment, wedge, retardance mismatch, fiber-mode filtering, or residual astigmatism.*

Response:

We agree that the previous version did not quantitatively explain the observed intensity scale, and this criticism motivated the most important revision.

(i) **Why the angular-only model fails, quantified.** The original model retained only the angular first-order corrections. For an ideal QWP at exact normal incidence, these terms vanish identically by symmetry ($\cot\theta = 0$ and $\mathrm{d}\Phi/\mathrm{d}\theta = 0$ at $\theta = 90^\circ$, hence $\bar{Q}_1 = \bar{Q}_2 = 0$; Supplementary Note S2). Allowing effective angular terms of representative magnitude — as would arise from a residual mount tilt — we obtain an *upper-bound* estimate of the normalized lobe intensity,

$$R_\theta^2 \sim \left(\frac{\omega_f}{f}\left|\frac{\mathrm{d}\Phi}{\mathrm{d}\Theta}\right|\right)^2 \simeq \left(9.7 \times 10^{-5} \times 0.066\right)^2 \sim 4 \times 10^{-11},$$

where the slope $|\mathrm{d}\Phi/\mathrm{d}\Theta| \approx 0.066$ follows from the vendor angle-of-incidence retardance curve, $\Phi(\theta_{\mathrm{AOI}}) \simeq \Phi_0 - a\,\theta_{\mathrm{AOI}}^2$ with $a \approx 33\ \mathrm{rad}^{-1}$, evaluated at a residual plate tilt $\theta_{\mathrm{AOI}} \sim 1$ mrad (Supplementary Notes S3 and S8, Table S7). This is more than three orders of magnitude below the measured normalized lobe intensity of $\sim 10^{-7}$.

(ii) **The missing channel.** The revised theory therefore expands the QWP response to first order in both transverse momentum and position (Eq. (5) of the main text),

$$\hat{Q} \simeq \bar{Q}_0 + \frac{\hat{k}_p}{k_0}\bar{Q}_1 + \frac{\hat{k}_s}{k_0}\bar{Q}_2 + \hat{p}\,\bar{Q}_3 + \hat{s}\,\bar{Q}_4.$$

The real-space gradient matrices $\bar{Q}_3, \bar{Q}_4$ — generated by transverse variations of retardance, fast-axis orientation, wedge, coating or bonding stress — are *allowed at normal incidence*. After propagation through the confocal system, they enter the same odd first-order mode as the angular terms, but weighted by the Rayleigh length $\ell = k_0\omega_0^2/2$ (Eq. (7) of the main text):

$$G_p = \frac{1}{f}\,\mathrm{a}^\dagger\bar{Q}_1\mathrm{e_P} - \mathrm{i}\,\frac{k_0\omega_0^2}{2f}\,\mathrm{a}^\dagger\bar{Q}_3\mathrm{e_P}, \qquad \frac{k_0\omega_0^2}{2f} = \frac{\ell}{f} \approx 1.1 \times 10^3.$$

A retardance nonuniformity of only $\delta_{eff} \sim 7.3 \times 10^{-4}$ rad across the illuminated radius $\omega_0 \approx 2.8$ mm then yields the normalized lobe intensity $\sim 10^{-7}$ (Supplementary Note S3 F).

(iii) **The decisive experimental test.** The two channels have *opposite* beam-size dependence. Holding the fiber mode radius ω_m fixed and scanning the collimated radius ω_0 (so that $\omega_f = C/\omega_0$ with $C = \lambda f/\pi$), the normalized lobe intensities of the two channels follow

$$R_\theta^2 \propto \frac{1}{\omega_0^2\left(C^2 + \omega_m^2\omega_0^2\right)} \ \text{(angular)}, \qquad R_r^2 \propto \frac{\omega_0^2}{C^2 + \omega_m^2\omega_0^2} \ \text{(real-space)}.$$

R_θ^2 decreases monotonically; R_r^2 increases. The measured lobe signal increases with beam size for every plate tested (Fig. 3 of the main text; Supplementary Figs. S7 and S9).

(iv) **Fitted first-order coefficient magnitudes as cross-checks.** The fitted modal coefficients test the same prediction independently of the peak-intensity ratio. Over the measured beam size range, the residual on-axis leakage $|\boldsymbol{\eta_0}|$ stays nearly constant, while the fitted collected magnitude $|\mathbf{G}^{\mathbf{fit}}| = \boldsymbol{\Lambda}|\mathbf{G}|$ grows by a factor 2.9 — against 2.8 predicted by the real-space scaling $\boldsymbol{\Lambda\,\omega_0^2}$, with point-by-point agreement within $\approx \mathbf{12}\%$ — whereas the angular channel, which scales as $\boldsymbol{\Lambda}$ alone, would *decrease* by a factor $\approx \mathbf{1.6}$ (Supplementary Note S7, Table S4).

(v) **On the artifact list.** The revised interpretation does not compete with "waveplate imperfections, misalignment, wedge, or retardance mismatch": it quantifies exactly these first-order departures of a real QWP and shows how they become observable under cross-polarized confocal detection. To exclude trivial single-component or detection artifacts, we provide: direct free-space camera imaging showing the two-lobe field before the collecting objective (Supplementary Fig. S4); comparison of QWPs of different material, construction, and retardance order at two wavelengths (Supplementary Fig. S5); tilt-angle sweeps (Supplementary Fig. S10); rotation of the mode with the QWP fast axis, which excludes a fixed astigmatism of the setup (Supplementary Fig. S12); and Mach–Zehnder interferometric wavefront measurements (Supplementary Note S6).

Major concern 2 — Limited novelty for Nature Communications

> *Reviewer comment: The novelty also appears limited for Nature Communications. Spin–orbit effects induced by anisotropic plates, tilted polarizers, and confocal cross-polarization geometries have been studied previously, including the mirror-based confocal extinction work that the authors themselves cite. The present manuscript replaces or supplements such elements with a QWP, but the advance is mainly an experimental observation in a specific optical setup rather than a broadly generalizable principle. The claimed applications in resonant spectroscopy, imaging, quantum optics, and mode engineering are speculative and not demonstrated.*

Response:

We have sharpened the statement of novelty and removed speculative application language from the Introduction and Discussion. The advances of the revised manuscript are:

- **Physical regime:** prior experimental work on waveplate-induced spin–orbit effects uses a finite, *intentional tilt* as the controlled symmetry-breaking parameter, with a *beam shift* as the main observable. Our experiment operates at nominal normal incidence — the standard configuration of commercial QWPs and of polarization-rejection confocal microscopy — where the established angular terms are symmetry-forbidden, and shows that first-order corrections nevertheless become directly observable in a cross-polarized confocal detection.
- **Mechanism and intensity scale:** angular and spatial first-order corrections produce the same two-lobe symmetry but at different intensity scales and with opposite beam-size scaling. The beam-size experiments show that real-space QWP nonuniformity— a channel

not treated in the prior momentum-domain literature— is the dominant contribution to the observed signal.

- **Practical result:** an extinction ratio of $\sim 10^7$ with a standard commercial quartz QWP, and exceeding 10^9 with a polymer zero-order QWP, with no mirror, custom aperture, or metasurface, providing a directly implementable route to improved polarization rejection in confocal spectroscopy.

Technical point 1 — Beam reshaping vs. detection-mode filtering

> *Reviewer comment: First, the role of the single-mode fiber is central, but the separation between true beam reshaping and detection-mode filtering is not rigorously quantified.*

Response:

We address this point both experimentally and theoretically. Direct free-space camera imaging, recorded after the analyzer and *before* the collecting objective, shows the two-lobe pattern in the optical field itself (Supplementary Note S4 and Fig. S4). In theory, the single-mode fiber is modeled explicitly as a Gaussian mode filter: the detected amplitude is the overlap integral between the focal-plane field and the fiber mode, and the finite fiber mode enters the odd first-order term only through the centroid factor

$$\Lambda = \frac{\omega_f^2}{\omega_f^2 + \omega_m^2},$$

leaving the *parity* of the collected mode unchanged (Supplementary Notes S2 and S3). The fiber therefore samples — but does not generate — the two-lobe structure; its Gaussian mode suppresses the odd first-order component at the beam center, which is precisely what enhances the extinction ratio. This distinction is now stated explicitly in the main text and the Supplementary Materials.

Technical point 2 — Systematic QWP/wavelength/incidence-angle comparison

> *Reviewer comment: Second, the QWP imperfections are invoked as a possible origin of the effect, yet no systematic comparison between different QWPs, retardance values, wavelengths, or incidence angles are provided.*

Response:

We have added the following systematic control measurements:

- At 850 nm: quartz zero-order QWP and polymer zero-order QWP (Supplementary Fig. S5).
- At 633 nm (He–Ne source): quartz zero-order, quartz multi-order, and MgF_2 zero-order QWPs (Supplementary Fig. S5).
- Incidence-angle (tilt) dependence (Supplementary Fig. S10).
- Beam-size dependence for several QWPs, by two independent methods (Fig. 3 of the main text, Supplementary Figs. S6-S9).

The two-lobe mode conversion and the extinction-ratio enhancement are reproduced across all plates and both wavelengths, while the absolute extinction performance is plate-dependent, consistent with element-specific first-order coefficients. The beam-size scaling is positive in every case tested.

Technical point 3 — Extinction enhancement lower than mirror-based work

> *Reviewer comment: Third, the extinction enhancement is only about, lower than previously reported mirror-based configurations, so the practical advantage is not compelling.*

Response:

The practical advantage of the QWP scheme lies in its simplicity, full compatibility and tunability with standard confocal microscopy: no central mirror, custom aperture, or metasurface is required, and the element can be inserted into existing beam paths. Moreover, $\sim 10^7$ is not a fundamental limit of the approach. In the exact plane-wave limit, the polarizer–QWP–analyzer geometry admits *complete* cancellation of the mean leakage (Supplementary Note S1). We demonstrate an extinction ratio exceeding 10^9 with a polymer zero-order QWP (Supplementary Fig. S5).

Technical point 4 — Effective complex parameters lack physical origin

> *Reviewer comment: Fourth, the fitting procedure uses effective complex parameters without a clear physical origin, making the agreement between experiment and fit insufficient as evidence for the proposed mechanism.*

Response:

In the revised theory, the complex coefficients are no longer introduced as phenomenological fitting parameters. They are derived from the first-order Taylor expansion of the QWP Jones matrix in transverse momentum and position, followed by propagation, analyzer projection, and fiber-mode overlap (Supplementary Note S3). The angular and real-space derivative terms carry different phases — the real-space channel acquires the factor $-\mathrm{i}\, k_0 \omega_0^2/2$ relative to the angular one — which is why the effective coefficients G_x, G_y are complex. As a cross-check, the fitted first-order magnitude $\left|\mathrm{G}^{\mathrm{fit}}\right| = \Lambda|\mathrm{G}|$ follows the real-space scaling $\Lambda\omega_0^2$ (growth factor 2.9 measured vs. 2.8 predicted) and is inconsistent with the angular scaling Λ (predicted *decrease* by ≈ 1.6) — Supplementary Table S4.

Technical point 5 — Direct wavefront/phase characterization

> *Reviewer comment: Finally, the manuscript would benefit from direct wavefront/phase characterization rather than intensity-only modal maps.*

Response:

We have added Mach–Zehnder interferometric measurements at 633 nm on the quartz and polymer zero-order plates (Supplementary Note S6, Figs. S15–S16, Table S1). After removal of the best linear phase ramp, the residual phase reveals spatial wavefront nonuniformity at the level of

$$\sigma_{\mathrm{res}} = 0.074 \text{ rad } (\lambda/85, \text{ quartz}), \qquad \sigma_{\mathrm{res}} = 0.140 \text{ rad } (\lambda/45, \text{ polymer}),$$

numerically below the vendor-specified transmitted wavefront errors ($\lambda/8$ and $< \lambda/4$, respectively). This residual nonuniformity is roughly two orders of magnitude above the $\sim 7 \times 10^{-4}$ rad effective nonuniformity implied by the fitted first-order coefficients (Supplementary Note S3), so only a small anisotropic fraction of the observed wavefront structure is required to supply the real-space channel.

Concluding remark

> *Reviewer comment: Overall, the work reports a potentially useful laboratory observation, but the physical interpretation remains incomplete and the conceptual advance is not strong enough for Nature Communications. I therefore recommend rejection.*

Response:

We thank the reviewer for the frank assessment, which directly shaped the revision. The two grounds for rejection — an incomplete physical interpretation and an insufficient conceptual advance — are addressed as follows. *First*, the interpretation is no longer phenomenological: the revised first-order theory quantitatively accounts for the mode structure, its orientation, and its intensity scale, and its distinguishing prediction (opposite beam-size scaling of the angular and spatial channels) is supported by the new beam-size experiments. *Second*, the conceptual advance is now stated precisely: real-space first-order nonuniformity of standard waveplates — a correction channel not considered by the established momentum-domain spin–orbit framework — becomes directly observable in cross-polarized confocal detection at normal incidence, where the established angular terms are symmetry-forbidden, with practical consequences for high-extinction microscopy.

Reviewer #2

We thank Reviewer #2 for the constructive comments and the concrete experimental suggestions. We have implemented essentially all of them in the revised manuscript, which substantially strengthened both the physical interpretation and the presentation.

General comment

> *Reviewer comment: The authors investigate a seemingly simple confocal setup, where light from one single-mode fiber is collimated, sent through polarizing components, and coupled into the receiving single-mode fiber. They find that introducing a QWP between two polarizers, and optimizing the angles, polarization extinction can be improved by 2 orders of magnitude compared to the case of only two polarizers. By spatial scanning the receiving SMF, as well as recording the collimated field with a camera, they find that a HG10-like structure appears, and since the overlap with the Gaussian fiber mode vanishes, this enhances the cross-polarization contrast. The authors show a nice derivation of a Fourier-optics model based on previous theoretical studies (in particular in the supplemental), and find good qualitative agreement to the data using some fit parameters.*

Response:

We thank the reviewer for the accurate summary. The revised manuscript develops the model further to include real-space first-order corrections of the QWP, which account for both the HG_{10}-like two-lobe profiles and the measured intensity scale. New control experiments (different QWPs, wavelengths, laser sources, beam sizes, and interferometric phase measurements) are included, largely following the reviewer's suggestions below.

Comment 1 — Definition of angles in Fig. 1a and definition of $\boldsymbol{\delta\alpha}$

> *Reviewer comment: fig.1a: the arced arrow indicating angles start at the origins, unclear to me (I guess 0 deg is at the right horizontal axis, isn't it?). — definition of δα: did I miss it, can't find in text or supplemental? I only read "δα around the symmetrically split mode." — can this be made precise or is this it?*

Response:

Both points are addressed. Fig. 1a has been revised: a reference line along the $+x$ axis is now drawn, and all arced arrows indicate angles measured counterclockwise from this axis. The caption now states: "All angles β, γ, α are measured counterclockwise from the laboratory $+x$ axis, viewed against the propagation direction; all elements are at nominal normal incidence."

The reviewer is right that $\delta\alpha$ was not properly defined. It is now defined explicitly at its first appearance in the main text and in the Methods ("Angle conventions"): $\delta\alpha \equiv \alpha - \alpha_{\max}$, where $\alpha_{\max}$ is the analyzer angle at which the maximum extinction ratio is obtained.

Comment 2 — Orientation of the QWP in the schematic (former Fig. S0)

> *Reviewer comment: Fig. S0: I see a QWP being illuminated from the side (i.e., rotated by 90 degrees around the x-axis compared to normal use) — I can't believe that this is intended — also Fig. S4 shows the expected case, or am I missing something?*

Response:

We thank the reviewer for spotting this. The schematic was indeed misleading and has been redrawn (now Supplementary Fig. S3). The caption and the accompanying text now also define the angle θ that enters the angular-response coefficients: it is the angle between the optic axis of the plate and the wavevector of the plane-wave component under consideration, equal to $90°$ for the central component at exact normal incidence. The same θ is used consistently in the tilt-dependence figure (Supplementary Fig. S10).

Comment 3 — More intuitive explanation of the $\mathbf{HG_{10}}$ pattern and its rotation

> *Reviewer comment: The HG10 pattern and its angular orientation, the discussion thereof I find a bit descriptive — because of the clear origin, the spatial derivatives in the formulae, in addition to the retardation changes with tilts, I would say that a more intuitive explanation should be possible! I read only "This behaviour is naturally understood as a polarization-dependent reorientation of the transverse field distribution.", which probably doesn't satisfy many readers!*

Response:

We agree and have replaced that sentence with an explicit physical picture (in the main text, section "Orientation control and the intensity of the rejected mode"). The cross-polarized field is the sum of a weak residual Gaussian term (η_0) and first-order transverse-derivative terms of the incident Gaussian, with complex weights G_x, G_y:

$$E_{\perp}(x_0, y_0) \propto \exp\left[-\frac{x_0^2 + y_0^2}{\omega_f^2 + \omega_m^2}\right]\left[\eta_0 + \Lambda\left(G_x x_0 + G_y y_0\right)\right].$$

Because the first derivative of a Gaussian changes sign across the beam center, these terms produce a two-lobe profile with a node at the center. Equivalently, near maximum extinction, the rejected field is a **directional derivative** $\partial_{\hat{u}}$ of the incident Gaussian along an axis $\hat{u}$ set by the QWP fast-axis angle γ; the intensity node lies perpendicular to $\hat{u}$, so rotating the QWP rotates $\hat{u}$ and carries the two-lobe pattern with it. In the scalar model, rotating the QWP redistributes the response between the x- and y-derivative channels (G_x, G_y). Near maximum extinction, the interference between the residual η_0 term and the derivative terms also accounts for the observed peak trajectories, including the avoided-crossing behavior of the peak positions (Supplementary Fig. S11).

Comment 4 — The intensity "mystery": quantification and component-exchange tests

> *Reviewer comment: About the mystery of high intensity of the cross-polarized HG10 field, mentioned at the very end of the manuscript:*
>
> *— I can't find any data about this — adding details of how much would be nice!*

> *— So, either the model is wrong, or, IMO more likely, some or more assumptions about the experiment are wrong. For nat.commun, I find a few tests are suitable:*
>
> *— An obvious attempt, which I don't see in the manuscript, is exchanging optical components, in particular the QWP and the light source.*
>
> *— The laser type is not specified — but even with excellent single-frequency diode lasers, the sidebands often carry 1e-5 of the laser intensity, which might lead to dispersive issues. (HeNe?)*
>
> *— The QWP is composed of two multi-order plates made from quartz — I think trying a different model, or a low-order simpler QWP, would be not too hard. And despite the plate has anti-reflection coatings (which might be strained), multiple reflections do occur.*

Response:

The reviewer's intuition was exactly right: the model, not the experiment, was incomplete — it lacked the real-space first-order response of the plate. We have addressed each point:

- **Quantification of the discrepancy**: The discrepancy is now stated explicitly. The angular-only upper-bound estimate gives a normalized lobe intensity $\sim 4 \times 10^{-11}$, against a measured $\sim 10^{-7}$ — more than three orders of magnitude below the measured value (Supplementary Note S3). This is an *upper bound*, since the ideal normal-incidence angular terms are exactly zero. Including the real-space gradient terms reproduces the measured scale for a retardance nonuniformity consistent with the fitted first-order coefficients (Supplementary Note S3). Figure 2 now compares the experimental maps with both the angular-only and the angular-plus-spatial models; fitted parameters and residuals are tabulated in Supplementary Note S7.
- **Exchanging components — QWP**: we compared quartz zero-order and polymer zero-order plates at 850 nm, and quartz zero-order, quartz multi-order, and MgF_2 zero-order plates at 633 nm (Supplementary Fig. S5). The two-lobe structure and the extinction enhancement are reproduced across all plates; the absolute extinction and lobe intensity are plate-dependent, consistent with element-specific first-order coefficients. The beam-size scaling is positive for every plate (Supplementary Fig. S7).
- **Exchanging components — light source**: the 850 nm source is now specified in the Methods and in Supplementary Note S4 (diode laser module LDT-850-10G, TOPAG Lasertechnik). All 633 nm control measurements were performed with a HeNe laser (HNL100LB, Thorlabs), as the reviewer suggested. The effect persists with the HeNe source, arguing against diode-laser spectral sidebands or source-specific dispersive artifacts as the origin.
- **Multiple reflections and coating strain**: to leading order, coherent multiple reflections between the plate surfaces act as a spatially uniform etalon contribution that renormalizes the effective retardance; they do not by themselves generate an odd first-order transverse mode. Spatially *nonuniform* coating stress, by contrast, is exactly the kind of contribution captured by $\bar{Q}_3, \bar{Q}_4$, and it is now listed among their physical sources. The persistence of the effect across plates of different construction and coating (quartz, polymer, MgF_2) argues against a single plate's coating being responsible.

We agree that quantitative agreement at this level is demanding. The beam-size-dependence experiment (Fig. 3; Supplementary Figs. S6–S9) provides the discriminating test: the lobe signal increases with beam size for every plate tested, identifying the real-space channel as the dominant contribution.

Comment 5 — Optical activity of quartz

> *Reviewer comment: More crazy, but could the (small) optical activity of quartz play a role? It would be easy to test MgF2 QWPs, they should not show optical activity or circular dichroism.*

Response:

We thank the reviewer for this suggestion, which we implemented directly. The extinction-ratio enhancement and comparable two-lobe intensities are also observed with a polymer zero-order QWP and with a $\mathrm{MgF_2}$ zero-order QWP (Supplementary Fig. S5), neither of which exhibits the optical activity of quartz. This rules out quartz optical activity as the dominant origin. A statement to this effect has been added to the main text (section "Beam-size scaling and control experiments").

Reviewer #3

We thank Reviewer #3 for the favorable assessment of the experimental results and for the well-posed suggestion: to check whether the observed mode transformation can be explained by a full solution of Maxwell's equations — i.e., the non-paraxial field of the nearly diffraction-limited source, collimated via the Richards–Wolf formalism — while retaining the standard, spatially uniform Jones matrix for the QWP. We have carried out exactly this calculation (new Supplementary Note S5).

General comment

> *Reviewer comment: In the manuscript, Wenze Lan et al. demonstrate a mode transformation in a paraxial beam obtained by collimating a nearly diffraction-limited Gaussian beam (λ = 850 nm and ω_0 = 2.5 μm) using a microscope objective (NA = 0.25). The authors present solid experimental evidence of this mode transformation when a quarter-wave plate is placed between two crossed polarizers. This phenomenon is indeed unexpected in paraxial optics, where polarizers and quarter-wave plates are not typically associated with spatial mode transformations.*
>
> *Although most of the measurements were performed by refocusing and coupling the collimated beam into an optical fiber, the authors also provide, in the supplementary material, images acquired with a camera placed in the collimated portion of the beam. They propose a modification of the Jones matrix of the quarter-wave plate to account for this phenomenon.*

Response:

We thank the reviewer for the accurate summary. The key physical question raised — whether the observed mode transformation can be attributed to non-paraxial propagation alone, or whether a spatially corrected QWP description is necessary — directly motivated the new calculation described below.

Main comment — Full non-paraxial calculation with the standard QWP Jones matrix

> *Reviewer comment: Since the source is a nearly diffraction-limited Gaussian beam, I would recommend computing the full solution of Maxwell's equations for such a beam in order to account for the longitudinal field component. One should then model the subsequent collimation of this field by a microscope objective using the Richards–Wolf formalism for a lens of arbitrary numerical aperture.*
>
> *I would be in favor of publication of this paper as it stands if the authors could provide simulations that retain the standard Jones matrix for the quarter-wave plate while properly accounting for the non-paraxial nature of the nearly diffraction-limited Gaussian beam and its collimation by a 0.25 NA objective, and demonstrate that this model (i.e., a full solution of Maxwell's equations) is still insufficient to account for the observed mode transformations.*

Response:

The calculation the reviewer requests — a full solution of Maxwell's equations for the fiber field, collimated by the objective via the Richards–Wolf formalism, with the QWP retained as an ideal,

spatially uniform Jones matrix — cannot account for the observed mode transformation. We carried out this calculation (new Supplementary Note S5), using the experimental parameters ($\lambda = 850$ nm, $\omega_m = 2.5\ \mu$m, NA = 0.25, $f = 26$ mm) and retaining the longitudinal field component throughout, and enforcing transversality exactly for every plane-wave component of the angular spectrum. The calculation fails on three independent counts:

(1) **Modal parity.** At the analyzer angle that optimizes the extinction (calibrated to the measured extinction ratio, ER = 4.2×10^7), the transmitted transverse cross-polarized field is a node-free Gaussian: the dominant component, $\left|E_y\right|^2$, relative peak intensity of 2.38×10^{-8} — not a first-order two-lobe mode (Supplementary Fig. S14b). Spatially uniform Jones matrices multiply azimuthal harmonics by scalars; they cannot convert an $n = 0$ Gaussian into an $n = 1$ derivative mode. This is a difference of *parity*, not of magnitude, and no choice of α, β, γ removes it.

(2) **The longitudinal field cannot supply the mode.** The calculation does produce a two-lobe longitudinal component, but transversality bounds it for a specified collimated beam of radius ω_0:

$$\frac{\max|E_z|}{\max|E_x|} = \frac{0.86}{k_0\omega_0} = 4.1 \times 10^{-5} \quad \Rightarrow \quad \frac{\max|E_z|^2}{\max|E_x|^2} = 1.7 \times 10^{-9},$$

In the cross-polarized channel, this gives a longitudinal peak of4.07×10^{-17} relative to the co-polarized peak, more than nine orders of magnitude below the measured cross-polarized intensity of order 10^{-7}. Its orientation is moreover tied to the transmitted polarization axis, rotating by 90° between the co- and cross-polarized settings, rather than continuously tracking the QWP fast-axis angle as observed experimentally (Fig. 4 of the main text). Independently, direct free-space camera imaging shows that the two-lobe transverse pattern is already present in the collimated beam before the focusing objective (Supplementary Fig. S4), confirming that the observed mode is a transverse field structure generated upstream of any focusing-induced longitudinal component.

(3) **The intrinsic non-paraxial background is the wrong mode.** The cross-polarized transverse background of the collimated beam, immediately after the collimating objective and before the polarizer, is a four-lobe, even, $\sin 2\phi_r$ distribution of relative peak intensity $\approx 1.18 \times 10^{-6}$, locked to the frame set by the input polarization and the collimation geometry, and it does not rotate with the QWP. We stress that this background is not negligible at our extinction levels, so we do not dismiss it on magnitude grounds. It is excluded instead because (i) it lies in the polarization component orthogonal to the input and is therefore removed by the polarizer, which precedes the QWP; and (ii) consistent with the coupling rule used throughout our first-order model (Supplementary Notes S2, S3, an even $\mathrm{m} = 2$ azimuthal component couples to the displaced Gaussian fiber mode only at second order in the scan coordinates, whereas the odd $\mathrm{m} = 1$ derivative mode couples linearly through Λ. Consistently, no four-lobe component is resolved in the measured maps at the extinction optimum.

In summary, non-paraxial propagation is real and, at these extinction levels, not negligible — but a full solution of Maxwell's equations with an ideal, spatially uniform QWP reproduces neither

the parity nor the magnitude nor the QWP-controlled orientation of the observed mode. The full derivation and numerical evaluation are given in Supplementary Note S5.

Concluding remark

> *Reviewer comment: The experimental results are interesting, as they could potentially indicate that the boundary between paraxial and non-paraxial optics is not as well defined as commonly assumed. In some works, non-paraxial effects are considered to become relevant for objectives with NA > 0.5 or NA > 0.6. However, in regimes where intensity differences on the order of 5 to 7 orders of magnitude can be measured and are relevant, as shown in Fig. 1(c) and 1(d), these distinctions may become less clear. Alternatively, these experimental results may demonstrate that it is indeed necessary to consider a modification of the Jones matrix of the quarter-wave plate.*

Response:

We agree, and the revised manuscript adopts both elements of this interpretation. First, when polarization extinction is measured over a dynamic range of 10^5–10^9, contributions that are normally negligible become experimentally relevant even at NA = 0.25: the Richards–Wolf calculation gives a finite non-paraxial background at the $\sim 10^{-6}$ level that cannot be ignored at such extinction. Second, this background has the wrong modal parity and no wave-plate dependence, so the dominant observation — the rotatable first-order two-lobe mode — does require a modification of the QWP Jones matrix, which we implement as the first-order expansion in transverse momentum and position.

Reviewer #4

We thank Reviewer #4 for the technically deep reading and for spelling out the connection to the momentum-domain Jones-matrix framework developed by the Bliokh group and by Ghosh and collaborators. The reviewer is correct that the angular part of our analysis rests on foundations established in Bliokh et al., Optica 3, 1039 (2016); Opt. Lett. 44, 4781 (2019); and Modak et al., Phys. Rev. A 105, 033713 (2022), and we apologize for the insufficient acknowledgment in the original submission. The introduction, theory section, and discussion have been rewritten to credit this framework explicitly and to state precisely what the present work adds.

General comment

> *Reviewer comment: The paper reports an interesting effect associated with spin orbit interaction (SOI) of light beam in a uniaxial anisotropic medium (waveplate) in confocal microscopy geometry. The authors have experimentally demonstrated SOI effect of fundamental Gaussian beam of light in confocal detection geometry when a quarter waveplate (QWP) is kept between crossed liner polarizers, leading to reshaping of the transverse field structure of the input Gaussian beam. They also demonstrated enhancement of extinction ratio of the crossed polarizers due to this SOI effect. The results are supported by corresponding theoretical formalism of SOI of light in uniaxial birefringent medium (waveplate). Based on these, the authors claim the novelty of this SOI effect in anisotropic medium observed in confocal geometry. SOI of light beams in various light matter interactions like partial and total internal reflection in dielectric interfaces, in tight focusing, in scattering and in inhomogeneous anisotropic (both birefringent and dichroic) medium, metasurfaces etc. has been widely explored in recent times, which have opened up exciting new avenues for controlled manipulation of light's field distribution using angular momentum degree of freedom of light and for the development of spin and orbital angular momentum-controlled photonic devices and systems. This work on SOI of light in uniaxial birefringent medium in confocal geometry is certainly timely and interesting.*
>
> *My main concern is on the novelty of the observed SOI effect and its fundamental origin in uniaxial anisotropic (birefringent) medium, which has been studied before (and the corresponding theoretical foundation is also well established) as I have noted down below. That said, I do agree observation of SOI in confocal geometry using a simple polarizer-QWP-polarizer combination and the resulting control on the spatial distribution of the field and the significant enhancement extinction ratio through SOI is expected to have useful applications.*

Response:

We thank the reviewer for the positive assessment of the experiment and for the precise identification of the theoretical context. The framing of the revised manuscript has changed accordingly: we no longer present the momentum-domain Jones-matrix expansion as a new theoretical foundation but explicitly identify it as the established framework on which the angular part of our analysis is built. Our contribution is now stated in two parts: (i) a standard QWP in a crossed-polarization confocal geometry converts the rejected field into a first-order spatial mode whose overlap with the collecting single-mode fiber is strongly suppressed, which directly improves the extinction ratio in a practical detection geometry; and (ii) at nominal normal incidence the established angular terms are symmetry-forbidden and fail, by more than three

orders of magnitude, to explain the measured intensity — which requires, and allows us to experimentally validate, first-order real-space corrections of real QWPs.

Main concern — Novelty in light of the momentum-domain Jones-matrix framework

> Reviewer comment: *The basic findings of the study can be fully explained using previously developed formalisms of SOI of light beam (having transverse wave vectors k_(x and) k_y) in uniaxial anisotropic medium exhibiting either linear diattenuation or dichroism (like polarizer) or linear retardance (waveplate) (OPTICA, 3, 1039, 2016, Optics Letters, 19, 4781, 2019 and Phys Rev A, 105, 033713, 2022). Unlike plane waves, for a light beam the polarization response of the anisotropic medium can be described by momentum domain Jones matrix J(k_(x/y)), which can be expanded around the zeroth order matrix (J(k_(x/y)=0), corresponding to the plane wave situation). This results in both in-plane (k_(x)) and out of plane (k_y) wave vector-dependent additional terms in the diagonal (k_x-dependent) and in the off-diagonal (k_y-dependent) elements of the Jones matrix. The latter k_y-dependent modification of the input field distribution is related to SOI of light as a consequence of the generation of k_y-dependent PB geometric phase of light. Once the symmetry of the uniaxial anisotropic medium is broken (e.g. by introducing slight tilt), it results in the well-known Spin Hall or Imbert-Fedorov (IF) shifts (having eigen polarization states of circular / elliptical polarizations). The k_x-dependent modification of the input field on the other hand leads to analogous Goose-Hanchen (GH) shift. In fact, these formalisms presented in the papers mentioned above have one to one correspondence to the usual transverse IF and longitudinal GH shift matrices (which can be directly derived from J(k_(x/y))) obtained for partial or total internal reflection at dielectric interfaces having very similar origin. Note that Eq. 1-5 presented in the current manuscript can also be directly derived from this formalism. Here the authors have investigated a QWP (having linear retardance and no diattenuation) and in this scenario one is concerned about the k_y-dependent modification of the field and the resulting transverse Spin Hall shift. Now in this scenario when a crossed polarizer is inserted, this will act like a standard weak measurement experiment of Spin Hall shift with exact orthogonal pre-post selection of polarization states. It is well known that in such scenario (of exact orthogonal pre-post selection using crossed-linear polarizer) one obtains Hermite-Gaussian like two lobe field structure, which has been observed by the authors. As has also been demonstrated earlier (Phys Rev A, 105, 033713, 2022), the symmetry here is decided by the orientation axis of the anisotropic medium (here the QWP), and thus the field structure can be rotated by rotating the anisotropy axis. Thus, the results obtained in this study can be completely explained by the already existing formalism of SOI of light in anisotropic medium with broken symmetry. The authors therefore need to justify and provide concrete evidence of the novelty of their work in light of the above discussion. In my understanding the only new findings is observation of the same effect in confocal arrangement, which of course will have practical applications. But whether that alone represent sufficient novelty is debatable.*

Response:

The angular part of our model is indeed directly contained in the established momentum-domain formalism: the k_p-dependent terms correspond to Goos–Hänchen-type corrections, the k_s-dependent terms to Imbert–Fedorov/spin-Hall-type corrections, and exactly crossed post-selection converts the weak transverse correction into an HG-like two-lobe distribution, as demonstrated in the cited literature. All of this is now acknowledged explicitly in the Introduction, the theory section and the Discussion.

However — *and this is the decisive point established by the revision* — the momentum-domain mechanism alone is quantitatively insufficient for our measurements. In our nominally normal-incidence geometry, the first-order angular terms are symmetry-forbidden ($\cot\theta = 0$ and $\mathrm{d}\Phi/\mathrm{d}\theta = 0$ at $\theta = 90^\circ$, so $\bar{Q}_1 = \bar{Q}_2 = 0$; Supplementary Note S2). Even allowing effective angular terms of representative magnitude — generated by a residual mount tilt of ~ 1 mrad and bounded with the vendor angle-of-incidence retardance data — the predicted lobe intensity

is $\sim 4 \times 10^{-11}$, against a measured $\sim 10^{-7}$: more than three orders of magnitude too small (Supplementary Note S3).

We therefore extend the expansion of the QWP response to first order in **both** transverse momentum and transverse position (Eq. (5) of the main text):

$$\hat{\boldsymbol{Q}} \simeq \bar{\boldsymbol{Q}}_{\boldsymbol{0}} + \frac{\hat{\boldsymbol{k}}_p}{\boldsymbol{k}_{\boldsymbol{0}}} \bar{\boldsymbol{Q}}_{\boldsymbol{1}} + \frac{\hat{\boldsymbol{k}}_s}{\boldsymbol{k}_{\boldsymbol{0}}} \bar{\boldsymbol{Q}}_{\boldsymbol{2}} + \hat{\boldsymbol{p}}\, \bar{\boldsymbol{Q}}_{\boldsymbol{3}} + \hat{\boldsymbol{s}}\, \bar{\boldsymbol{Q}}_{\boldsymbol{4}},$$

where $\bar{Q}_1, \bar{Q}_2$ reproduce the reviewer's $J_{k_{x,y}}$ expansion, while $\bar{Q}_3, \bar{Q}_4$ are the first real-space Taylor coefficients of the *physical* plate — gradients of retardance and fast-axis orientation, wedge, coating or bonding stress. After cross-polarized projection, both channels feed the same odd first-order mode, but with different physical scaling: the real-space channel is weighted by the Rayleigh length and amplified by $k_0\omega_0^2/2f \approx 1.1 \times 10^3$ (Eq. (7) of the main text), which reproduces the measured lobe intensity for a physically reasonable plate nonuniformity.

The concrete evidence the reviewer requests is the beam-size experiment. The two channels predict *opposite* trends of the normalized lobe intensity with the collimated beam radius (Eqs. (8) and (9) of the main text):

$$R_\Theta^2 \;\propto\; \frac{1}{\omega_0^2(C^2 + \omega_m^2\omega_0^2)} \text{ (angular)}, \qquad R_r^2 \;\propto\; \frac{\omega_0^2}{C^2 + \omega_m^2\omega_0^2} \text{ (real-space)},$$

with $C = \lambda f/\pi$. The measured signal **increases** with beam size for every plate tested, at every measured beam size, and for both independent methods of varying ω_0 (Fig. 3 of the main text; Supplementary Figs. S7 and S9). The fitted first-order coefficients give the same answer in a shape-resolved way: $|\mathrm{G}^{\mathrm{fit}}|$ grows by a factor 2.9, against 2.8 predicted for the real-space channel and a *decrease* by ≈ 1.6 predicted for the angular channel (Supplementary Table S4).

The confocal geometry is thus not merely a new location in which to observe a known beam shift: it changes the observable from a centroid displacement to a mode-overlap problem and thereby exposes a class of real-plate spatial corrections that remain hidden in standard weak-measurement beam-shift experiments.

Comment — Prior work not adequately represented in Introduction and theory section

> *Reviewer comment: The introduction section and also the theory section does not adequately bring out the existing work in the domain of SOI of light in uniaxial anisotropic medium (the works mentioned above and other related work). Specifically, the theory section is misleading and the authors should clearly present the existing models of momentum domain Jones matrix formalism as discussed above. They should also highlight if there is any new theoretical foundations of this effect based on the already existing models in literature.*

Response:

We agree, and the Introduction and theory section have been rewritten. The Introduction now presents the momentum-domain Jones-matrix expansion for tilted birefringent plates and tilted polarizers, the connection to Goos–Hänchen and Imbert–Fedorov shifts, weak-measurement beam-shift experiments, and prior demonstrations of HG-like distributions under near-orthogonal pre/post-selection, with the appropriate citations. The theory section is organized in four steps:

- the established momentum-domain framework, presented as such and with explicit citation of its origins (Eqs. (1)-(4) of the main text; Supplementary Note S2);
- the demonstration that crossed-polarizer projection converts the first-order transverse correction into an HG-like two-lobe field;
- the quantitative statement of *why the angular-only model fails* in our geometry (more than 3 orders of magnitude in intensity, and the wrong sign of the beam-size trend);
- the real-space first-order correction as the minimal extension required for a real QWP in a high-extinction confocal experiment (Eq. (5); Supplementary Note S3).

The text states explicitly that step 4 is an *extension of*, not a replacement for, the established theory, and that the two sets of coefficients cannot be separated by a static modal fit — only by their beam-size scaling.

Comment — Symmetry-breaking mechanism should be specified quantitatively

> *Reviewer comment: The SOI effect arises from symmetry breaking in the QWP (like introducing small tilt in previous papers). The authors state that in their experiment symmetry breaking arises from imperfections of the QWP. This appears a little vague. The authors need to provide exact mechanisms of symmetry breaking and a quantitative description of the same (like in previous papers results have always been presented in terms of the tilt angle).*

Response:

We agree that "QWP imperfections" was too vague and have replaced it by an explicit parametrization. In tilted-plate experiments, the symmetry-breaking parameter is a single controlled macroscopic tilt angle. In our nominally normal-incidence geometry, the symmetry-breaking parameters are the first-order coefficients of the expanded QWP response, in two classes:

(1) Angular coefficients $\bar{Q}_1, \bar{Q}_2$ — residual angle of incidence and any small effective plate tilt. These are the direct analogue of the tilted-plate formalism, and we bound them using the vendor angle-of-incidence retardance data (Supplementary Note S8, Table S7), which give the parabolic law $\Phi(\theta_{\mathrm{AOI}}) \simeq \Phi_0 - a\,\theta_{\mathrm{AOI}}^2$ with $a \approx 33\ \mathrm{rad}^{-1}$, hence

$$\left|\frac{\mathrm{d}\Phi}{\mathrm{d}\theta}\right| = 2a\,\theta_{\mathrm{AOI}} \approx 0.066 \quad \text{for a residual mount tilt } \theta_{\mathrm{AOI}} \sim 1\ \mathrm{mrad}.$$

(2) Spatial coefficients $\bar{Q}_3, \bar{Q}_4$ — first-order transverse gradients of local retardance and fast-axis orientation, together with wedge-related phase and coating/bonding stress gradients across the illuminated aperture:

$$\bar{Q}_{3,4} = \frac{\partial \bar{Q}}{\partial \Phi} \partial_{p,s}\Phi + \frac{\partial \bar{Q}}{\partial \gamma} \partial_{p,s}\gamma + \cdots$$

After analyzer projection, these terms are summarized by the complex vector $\mathrm{G} = (G_x, G_y)$, whose direction and magnitude set the two-lobe orientation and intensity after fiber-mode convolution. G is extracted from the measured maps as a function of analyzer angle, QWP fast-axis angle and beam radius (Supplementary Note S7, Tables S2, S3 and S5), and the extracted lobe orientation follows the QWP fast-axis angle as predicted (Supplementary Fig. S12).

Quantitatively, inverting the fitted first-order coefficient gives the scale of this nonuniformity directly: at the largest beam radius, $|\mathrm{G}^{\mathrm{fit}}| = 0.146\ \mathrm{mm}^{-1}$ with $\Lambda = 0.503$ and $\ell/f = 1.11 \times 10^3$ corresponds to an effective analyzer-projected Jones nonuniformity of

$$\delta_{\mathrm{eff}} = \frac{f}{\ell} \frac{|\mathrm{G}^{\mathrm{fit}}|}{\Lambda} \omega_0 \approx 7.3 \times 10^{-4}$$

across the illuminated radius (Supplementary Note S3). This dimensionless quantity is the fitted-coefficient measure of the symmetry-breaking scale; resolving it into a specific physical parameter (a retardance gradient, a fast-axis gradient, wedge, or coating-stress gradient) would require the spatially resolved vectorial measurement noted in the Discussion, since the confocal fit alone does not separate these individual contributions.

Concluding remark

> *Reviewer comment: In summary, I have noted my main concerns on the novelty of the foundation of the observed SOI effect of light beam in anisotropic medium, which the authors must address before it can be considered further. Otherwise, the results appear interesting, analysis and the methodology appears to be neat, the interpretations are also based on the underlying physical basis. The paper is clearly written and the results are presented in logical manner.*

Response:

We thank the reviewer for the constructive assessment. The revised manuscript identifies the angular/momentum-domain SOI formalism as established prior work and credits it explicitly, and states the specific advances of the present study: (i) a standard QWP in a cross-polarized confocal geometry produces a rejected spatial mode with strongly suppressed fiber-mode overlap, yielding a large and practically useful extinction-ratio enhancement; and (ii) the measured intensity and its beam-size dependence require extending the established angular theory by **real-space first-order QWP corrections**, which we introduce, bound quantitatively, and test experimentally through beam-size scaling, supported by interferometry and by a full vectorial Richards–Wolf calculation. We hope these revisions satisfactorily address the reviewer's concerns.

Point-by-Point Response to the Reviewers

Manuscript # NCOMMS-26-028643A

Title: Real-space spin–orbit interaction from quarter-wave-plate nonuniformity

We thank all four reviewers for their careful reading and positive feedback. All changes in the revised manuscript and Supplementary Materials are marked in blue. Below, reviewer comments are reproduced in shaded boxes, followed by our responses. All figure and note numbers refer to the revised version.

Reviewer #1

General comment

> *Reviewer comment: The authors have properly addressed all comments raised by me and other reviewers. Generally I am satisfied with the revisions. Therefore, I would recommend its publication.*

Response:

We thank the reviewer for the positive assessment and are pleased that our revisions have addressed all comments.

Reviewer #2

General comment

> *Reviewer comment: The authors have done additional experiments and extended their model and done full vectorial calculations. The authors now were able to find an explanation of the magnitude of the observed effects - spatial variations of the QWP, taken now into account by the real-space variation in Eq. 5 and explained in S3. One might say that it is obvious that spatial variations enter via higher-order spatial modes, but I didn't made this comments before and I find it surprising that they "amplify" the angular effects in the way the authors found now (which could be emphasized/explained more in the manuscript).*
>
> *The new experiments with varied beam collimation in Fig. 3, which has opposite effects on angular spread and beam size, are quite convincing. As a side-product, the authors found a new method to quantify uniformity of QWPs. The authors also did tests with different waveplates and light sources, making this in my opinion a complete story - with pretty new conclusions: The effect is mainly not due to angular but spatial effects, with very similar manifestations. I wonder how much this new insight changes the scientific charme of the manuscript. Probably, because the story is still conceptually nice and connects angular and spatial effects in one framework, this could still be seen to be similarly compelling as before.*
>
> *One point that might be interesting could be to analyze related precision experiments, for instance on beam shifts, or if the effects only appear in the particular geometry here.*

Response:

We thank the Reviewer for this thoughtful and positive assessment. We agree that an important outcome of the revision is that real-space and momentum-space variations can produce very similar first-order transverse-mode signatures, although they correspond to physically distinct mechanisms. In particular, the real-space nonuniformity does not simply amplify the angular contribution; rather, both channels can couple the input Gaussian mode to the same lowest-order odd spatial modes, while their opposite beam-size scaling allows them to be distinguished experimentally, as demonstrated in Fig. 3. We have further clarified this point on page 3 in the main text and emphasized that the real-space contribution complements, rather than replaces, the momentum-space spin-orbit picture:

"Importantly, this is not an amplification of the angular SOI itself: the angular and real-space terms are distinct channels that couple to the same first-order odd transverse mode, while the confocal propagation factor enhances the contribution of the real-space gradient."

We also thank the Reviewer for the interesting suggestion concerning related precision and beam-shift experiments. In general, we expect both real-space and momentum-space variations to be relevant, with their relative importance depending on the illumination and detection geometry. For example, when the relevant optical element or interface is sampled by a tightly focused beam, the smaller illuminated area tends to reduce the contribution from lateral nonuniformity, whereas the broader angular spectrum and subsequent propagation favor the manifestation of angular beam shifts; weak-value post-selection can further enhance such angular signatures. In contrast, our present geometry uses a comparatively large collimated beam at the QWP together with mode-selective confocal detection, making the real-space contribution particularly prominent. We have added a brief discussion of this more general perspective to the revised manuscript on page 5:

"This comparison also suggests why the relative weight of the two channels can differ strongly across precision beam-shift experiments. When a laterally nonuniform polarization element is sampled near a focused waist, the smaller illuminated area suppresses the first-order real-space-gradient contribution according to the beam-size scaling above, whereas the broader angular spectrum and subsequent propagation can enhance the manifestation of angular beam shifts [24]. Near-orthogonal post-selection can further amplify such weak angular signatures [7]. Thus, real- and momentum-space variations should in general be considered together, with their relative importance set by the illumination and detection geometry."

Reviewer #3

General comment

> *Reviewer comment: The authors have addressed the main comments and improved the overall structure of the paper. They have performed calculations based on a full vectorial model that verify that the mode transformation is not related to non-paraxial effects. They presented their results in Supplementary Note S5. I therefore agree with the publication of the manuscript.*

Response:

We thank the reviewer for the positive comments and support for publication.

Minor comment

> *Reviewer comment: In Fig. S13, the authors define $\theta'=\omega_0/z_r$, while in Eq. S79 they use $\theta'=\omega_0/l$. Please homogenize the notation.*

Response:

Thank you for catching this. We have corrected the notation in Supplementary Figure 13 to $\theta' \approx \omega_0/\ell$.

Reviewer #4

General comment

> *Reviewer comment: The authors have done a satisfactory job in addressing several of my concerns on the nature of SOI in tilted waveplate and the resemblance to the already established frameworks on SOI in such scenario and also to the standard weak measurement scenario related to beam shifts with near orthogonal pre-post selection of polarization states. Specifically, they have established the novelty of the present work by providing additional theoretical analysis, experimental results with proper citation to previous work that i had noted in my previous report. I can now recommend the revised manuscript for publication.*

Response:
We thank the reviewer for the positive remarks and recommendation for publication.
